# Engineered 3D immuno-glial-neurovascular human miBrain model

**DOI:** 10.1073/pnas.2511596122

**Published:** 2025-10-17

**Authors:** Alice E. Stanton, Adele Bubnys, Emre Agbas, Benjamin James, Dong Shin Park, Alan Jiang, Rebecca L. Pinals, Liwang Liu, Nhat Truong, Anjanet Loon, Colin Staab, Oyku Cerit, Hsin-Lan Wen, David Mankus, Margaret E. Bisher, Abigail K. R. Lytton-Jean, Manolis Kellis, Joel W. Blanchard, Robert Langer, Li-Huei Tsai

**Affiliations:** ^a^Koch Institute, Massachusetts Institute of Technology, Cambridge, MA 02139; ^b^Picower Institute for Learning and Memory, Massachusetts Institute of Technology, Cambridge, MA 02139; ^c^Department of Brain and Cognitive Sciences, Massachusetts Institute of Technology, Cambridge, MA 02139; ^d^Department of Electrical Engineering and Computer Science, Massachusetts Institute of Technology, Cambridge, MA 02139; ^e^Broad Institute of Harvard and Massachusetts Institute of Technology, Cambridge, MA 02139; ^f^Department of Anesthesiology, Boston Children’s Hospital, Boston, MA 02139; ^g^Department of Chemical Engineering, Massachusetts Institute of Technology, Cambridge, MA 02139; ^h^Division of Health Science and Technology, Massachusetts Institute of Technology, Cambridge, MA 02139; ^i^Institute for Medical Engineering and Science, Massachusetts Institute of Technology, Cambridge, MA 02139

**Keywords:** brain organoid, microphysiological system, neurovascular, neuro-immune, biomaterials

## Abstract

Patient-specific, human-based cellular models integrating a biomimetic blood–brain barrier, immune, and myelinated neuron components are critically needed to enable accelerated, translationally relevant discovery of neurological disease mechanisms and interventions. To construct a human cell-based model that includes these features and all six major brain cell types needed to mimic disease and dissect pathological mechanisms, we have constructed, characterized, and utilized a multicellular integrated brain (miBrain) immuno-glial-neurovascular model by engineering a brain-inspired 3D hydrogel and identifying conditions to coculture these six brain cell types, all differentiated from patient induced pluripotent stem cells. miBrains recapitulate in vivo*–*like hallmarks inclusive of neuronal activity, functional connectivity, barrier function, myelin-producing oligodendrocyte engagement with neurons, multicellular interactions, and transcriptomic profiles. We implemented the model to study Alzheimer’s Disease pathologies associated with *APOE4* genetic risk. *APOE4* miBrains differentially exhibit amyloid aggregation, tau phosphorylation, and astrocytic glial fibrillary acidic protein. Unlike the coemergent fate specification of glia and neurons in other organoid approaches, miBrains integrate independently differentiated cell types, a feature we harnessed to identify that *APOE4* in astrocytes promotes neuronal tau pathogenesis and dysregulation through crosstalk with microglia.

## Significance

To address the lack of human cell-based models incorporating all six of the major brain cell types together, which are critically needed to mimic features of brain pathobiology and accelerate mechanistic understanding and therapeutic testing, we have developed, characterized, and harnessed a patient-specific preclinical brain model composed of 3D immuno-glial-neurovascular units. This multicellular system with induced pluripotent stem cell (iPSC)-derived neurons, microglia, oligodendroglia, astrocytes, pericytes, and brain microvascular endothelial cells has potential for opening possibilities for disease modeling, drug discovery and development, and precision medicine-based approaches.

In vitro brain models hold enormous potential for decoding disease mechanisms and establishing enhanced drug screening platforms for the discovery and development of therapeutic interventions (1–6). Neurological disease treatments have been particularly hindered by the lack of human-based models, as genetic differences limit the pace and fidelity of therapeutic translation from rodent models to human (7), many genetic risk variants lie in noncoding regions, and there are marked differences between the human brain and that of other species (8). Organotypic brain slice cultures can be derived from patient biopsies and preserve the three-dimensional (3D) architecture of brain tissue, providing a useful tool (9, 10), though limited by the number of donors, donor diversity, patient heterogeneity in genetics, confounding lifestyle effects, and tunability of composition for mechanistic inquiry. Synergistically, induced pluripotent stem cell (iPSC) technology enables the generation of human patient-specific cells into almost any cell type in the body, including brain cell types (11, 12). More recently, brain organoids, harnessing iPSCs, have enhanced neuronal maturation and model neuronal development, wherein neuronal progenitors differentiate together into a dense coculture of neurons and glia with maintained progenitors (1–3, 13). Their utility has been further broadened by approaches to integrate microglial components by some (14, 15), and vascular-like cells by others (16, 17), though stable integration of all of these components, along with myelinating oligodendroglia has yet to be demonstrated. The dense cellular organization, which often leads to a necrotic core, further limits the homogeneous distribution and biomimicry. At the same time, others have taken directed approaches to construct microphysiological systems with tissue-mimicking 3D architecture in which cells of the desired lineages are combined in a 3D scaffold via cell self-assembly or via patterning (4, 5, 18–22). Complementarily, biomaterials engineering approaches have revealed mechanical and biochemical parameters to enhance the 3D culture of neural progenitor cells (23–25) and bioprinting approaches have been used to construct engineered tissues of neurons and astrocytes (26).

There is great interest in establishing an engineered 3D human cell-based model with all major brain cell types, given the key role of glial, immune, and vascular cells in processes of brain physiology and pathology, with increasing evidence suggesting that non–cell autonomous effects contribute critically to neurodegenerative diseases like Alzheimer’s (27–31). Disease-associated mutations are expressed by the various brain cell types to different extents and lead to disparate changes in cell phenotypes, further modified by multicellular interactions (32, 33). To identify cell-type- and pathway-specific contributions to pathogenesis, a brain model in which each cell type can be independently modified is further desirable. To establish a preclinical brain model inclusive of each of the six brain cell types to model neurodegenerative disease, dissect cell–cell interactions, and identify putative interventions, we have developed a 3D, iPSC-based multicellular integrated brain (miBrain) immuno-glial-neurovascular model incorporating each of six major brain cell types arranged in 3D tissue organization. Incorporating all iPSC-derived cells, miBrains can be constructed with specific genetic modifications and from specific patient cell lines, providing a tool with which the functional consequences of mutations can be probed and the cell–cell signaling pathways interrogated. As conventional 3D microphysiological systems utilize protein-based materials that mimic only the basement membrane (BM), such as Matrigel and collagen, or rely on fibrin, like which forms during blood clots and contains neurotoxic components (34), all of which rapidly degrade, we critically need a hydrogel mimetic of brain tissue beyond the BM, one which can also promote neuronal activity, while providing 3D structure and supporting cellular self-assembly. To this end, we engineered a 3D dextran-based hydrogel, Neuromatrix Hydrogel, incorporating brain extracellular matrix (ECM) proteins and the BM peptide mimic Arginylglycylaspartic acid (RGD) to provide enhanced brain mimicry and promote co-self-assembly of all six major central nervous system (CNS) cell types into an integral engineered tissue. As one proof of principle, we have leveraged the miBrain to investigate the strongest genetic risk factor for sporadic Alzheimer’s Disease (AD), apolipoprotein E4 (*APOE4*) (35). Importantly, we have modeled amyloid and tau pathologies in this integrated brain model and here uncovered that *APOE4* astrocytes are sufficient to induce reactive species and tau phosphorylation via crosstalk with microglia.

## Results

### Multicellular Integrated miBrain Model Coassembling 6 Major CNS Cell Types.

Many human neurological diseases involve a diversity of cell types beyond neurons, but there are currently no models that have both this full cellular diversity and human genetics (36). We endeavored to address this critical void by developing a platform that is inclusive of all six CNS cell types, recapitulates important features of 3D brain tissue structure, and decouples glial and neuronal fate specification, enabling introduction of cell-type-specific perturbations and genetic mutations. To this end, we separately differentiated, optimized, and validated each of the six major brain cell types from patient-specific iPSCs for: brain microvascular endothelial cells (BMECs) (37), pericytes (37, 38), oligodendrocyte precursor cells (OPCs) (39), neurons (40), astrocytes (41), and microglia-like cells (iMG) (42).

We validated that iPSC-derived BMECs express canonical markers via immunohistochemistry (*SI Appendix*, Fig. S1 *A* and *B*), gene expression (*SI Appendix*, Fig. S1 *C* and *D*), and flow cytometry (*SI Appendix*, Fig. S2 *A*, *i*). As evidence suggests that some endothelial cell differentiation protocols from iPSCs can lead to more epithelial-like lineages (43), we performed RNA-sequencing (RNAseq) on the BMECs used in this study and integrated the data with existing literature datasets (43–48). We found that our BMECs clustered with endothelial cells—upregulated endothelial processes (*SI Appendix*, Fig. S1 *C*, *i*) and downregulated epithelial processes (*SI Appendix*, Fig. S1 *C*, *ii*)—with comparable or greater expression of endothelial genes (*SI Appendix*, Fig. S1 *D*, *i*) and lesser gene expression of epithelial genes (*SI Appendix*, Fig. S1 *D*, *ii*) (43). Functionally, barrier resistance values for these BMECs were comparable to reported values for endothelial cells, as opposed to the much higher resistance in epithelial monolayers (43) (*SI Appendix*, Fig. S1*E*).

iPSC-derived pericytes were validated for protein expression of CD140b via flow cytometry (*SI Appendix*, Fig. S2 *A*, *ii*), NG2 and PDGFRβ via immunoreactivity (*SI Appendix*, Fig. S2 *B* and *C*), as well as for gene expression for canonical marker genes, such as *PDGFRβ, CSPG4,* and *VEGFA* (*SI Appendix*, Fig. S3 *A*, *i*) and genes shown to be upregulated in pericytes and downregulated in smooth muscle cells (49), such as *TGFβ1, DCHS1,* and *NUAK1* (*SI Appendix*, Fig. S3 *A*, *ii*). iPSC-derived astrocytes displayed strong protein expression of CD44 via flow cytometry (*SI Appendix*, Fig. S2 *A*, *iii*), S100β, glial fibrillary acidic protein (GFAP), ALDH1L1, and AQP4 via immunoreactivity (*SI Appendix*, Fig. S2 *B* and *D*), a lack of immunoreactivity to neuron progenitor marker SOX2 (*SI Appendix*, Fig. S2*B*), positive gene expression for canonical markers like *VIM* and *GFAP* (*SI Appendix*, Fig. S3*B*), and, functionally, calcium transients (*SI Appendix*, Fig. S4). iPSC-derived OPCs demonstrated PDGFRα protein expression via flow cytometry (*SI Appendix*, Fig. S2 *A*, *iv*), SOX10, Olig2, O4, and PDFRα immunoreactivity (*SI Appendix*, Fig. S2 *B* and *E*), and expression for identity genes *like PAX6, PDGFRα*, *and PLP1* (*SI Appendix*, Fig. S3*C*). iMG were validated for CD45 marker expression via flow cytometry (*SI Appendix*, Fig. S2 *A*, *vi*), immunoreactivity to Iba1, P2RY12, and TMEM119 (*SI Appendix*, Fig. S2 *B* and *G*), and expression for identity genes like *P2RY12, FOS,* and *CSF1R* (*SI Appendix*, Fig. S3*E*). iPSC-derived neurons displayed strong expression of excitatory cortical neuron marker PSA NCAM via flow cytometry (*SI Appendix*, Fig. S2 *A*, *v*), β-Tubulin, SOX2, synapsin, MAP2, and NeuN immunoreactivity, and a lack of immunoreactivity to pluripotency marker Oct4, here included because NGN2-neurons rely on piggyBac-mediated expression of the cassette, without which pluripotent cells could persistent (*SI Appendix*, Fig. S2 *B* and *F*), and canonical genes such as *MAP2, SCN1A,* and *NTRK2* (*SI Appendix*, Fig. S3*D*).

### Engineering a Brain Matrix-Inspired 3D Hydrogel.

**Fig. 1.**
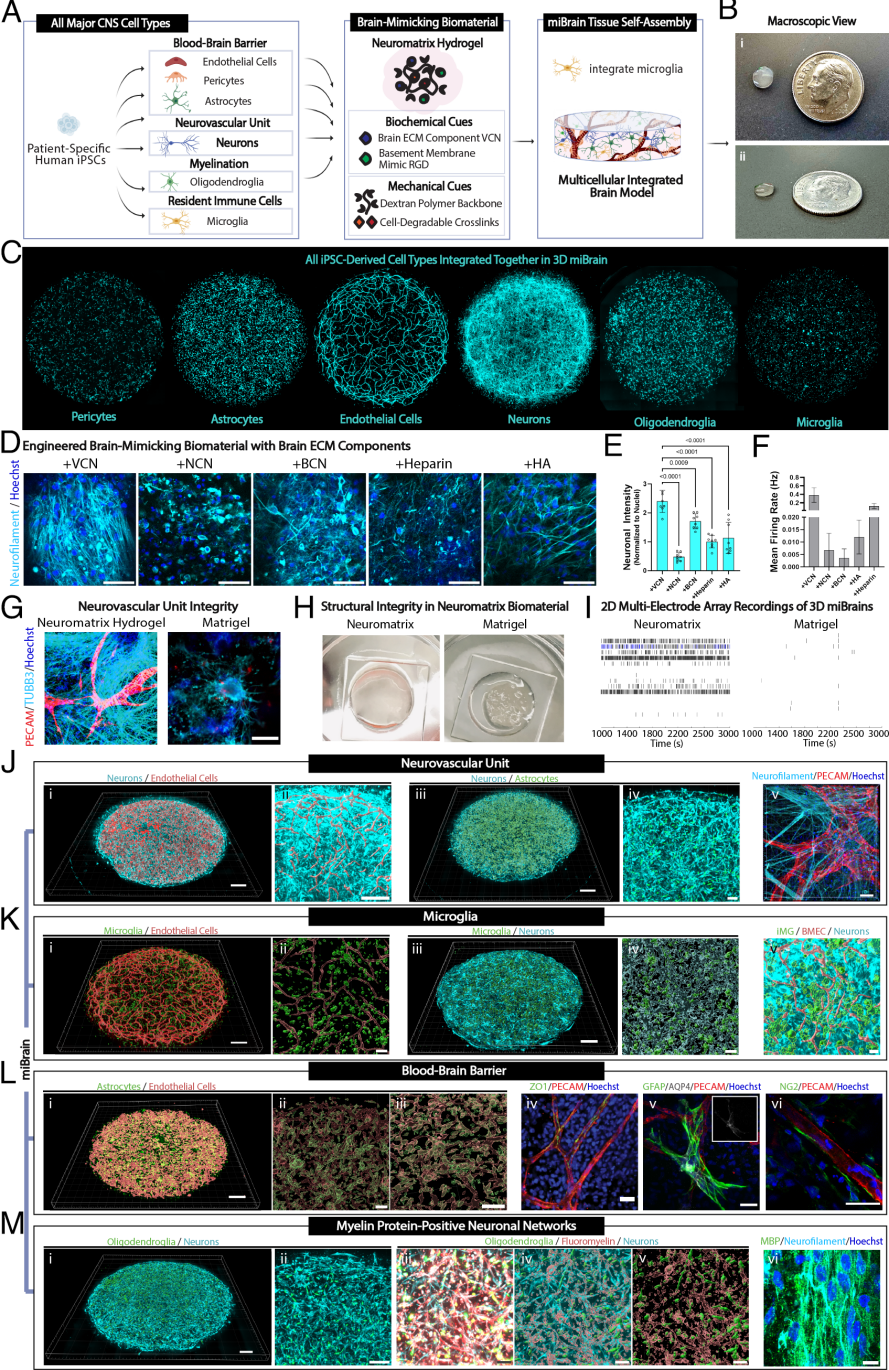
Human integrated 3D-immuno-glial-neurovascular miBrain model. (*A*) Schematic of miBrain formation (*B*) macroscopic view of miBrains from (*i*) *Top* and (*ii*) side view with a dime for reference, (*C*) distribution of (*Left* to *Right*) iPSC-derived pericytes (cyan: mCherry-pericytes), astrocytes (cyan: mCherry-astrocytes), iPSC-derived BMECs (cyan: mCherry-BMECs), neurons (cyan: tubulin), oligodendroglia (cyan: tdTomato pretransfected oligodendroglia), and iPSC-derived microglia (iMG) (cyan: membrane prelabeled iMG), (*D*) neuronal phenotypes in miBrains cultured in dextran-based hydrogels fabricated with various brain ECM proteins (cyan: neurofilament, blue: Hoechst); (Scale bar, 50 µm), (*E*) quantification of neurofilament immunoreactivity (averages of n = 8 samples, n = 3 FOV per sample, ordinary one-way ANOVA statistical test), (*F*) neuronal firing as assessed on an MEA system (n = 3 wells per group, mean, SEM, ordinary one-way ANOVA statistical test), (*G*) persistence of neurovascular unit phenotypes in miBrains cultured in Neuromatrix Hydrogel versus Matrigel after 5 wk in culture [red: PECAM, cyan: tubulin beta III (TUBB3), blue: Hoechst]; (Scale bar, 50 µm), (*H*) macroscopic view of gel structural integrity for miBrains cultured in versican (VCN)-incorporated engineered dextran-based hydrogel named Neuromatrix Hydrogel versus Matrigel after 4 wk, (*I*) example raster plots from MEA recordings of miBrains in Neuromatrix Hydrogel compared to Matrigel; miBrains recapitulate key hallmarks of human brain tissue, inclusive of (*J*) neurovascular units: (*i*) 3D BMEC and neuronal networks (red: mCherry-BMEC-surfaces, cyan: tubulin neuron label); (Scale bar, 500 µm), (*ii*) higher magnification (red: mCherry-BMEC-surfaces, cyan: siR-tubulin); (Scale bar, 500 µm), (*iii*) 3D integrated astrocyte and neuronal networks (green: mCherry-astrocyte-surfaces, cyan: siR-tubulin); (Scale bar, 500 µm), (*iv*) higher magnification (green: mCherry-astrocytes, cyan: tubulin); (Scale bar, 100 µm), and (*v*) miBrain 3D rendering (red: PECAM, cyan: neurofilament, blue: Hoechst); (Scale bar, 50 µm), (*K*) microglia: (*i*) 3D iMG distributed throughout BMEC networks (Imaris surfaces from red: mCherry-BMECs, green: membrane prelabeled iMG); (Scale bar, 500 µm), (*ii*) higher magnification (Imaris surfaces from red: mCherry-BMECs, green: membrane prelabeled iMG); (Scale bar, 100 µm), (*iii*) 3D iMG distributed throughout neuronal networks (cyan: tubulin, green: membrane prelabeled iMG-surfaces); (Scale bar, 500 µm), (*iv*) higher magnification (Imaris reconstruction of cyan: tubulin, green: membrane prelabeled iMG); (Scale bar, 100 µm), and (*v*) distribution of iMG with BMEC and neuronal networks (cyan: tubulin; Imaris surfaces from red: mCherry-BMECs, green: membrane prelabeled iMG); (Scale bar, 100 µm), (*L*) BBB: (*i*) 3D astrocytes distributed throughout BMEC networks (red: Imaris surfaces from ZO1-BMECs, green: mCherry-astrocytes); (Scale bar, 500 µm), (*ii*) higher magnification [Imaris surfaces from red: zona occludens-1 (ZO-1)-BMECs, green: mCherry-astrocytes]; (Scale bar, 100 µm), (*iii*) magnified (Scale bar, 50 µm), (*iv*) ZO-1 tight junctions (green: ZO-1, red: PECAM, blue: Hoechst); (Scale bar, 30 µm), (*v*) astrocytes at the vasculature [green: GFAP, gray: aquaporin-4 (AQP4), red: PECAM, blue: Hoechst; (Scale bar, 30 µm); insert, gray: AQP4], and (*vi*) pericytes at the vasculature [green: neuron-glial antigen 2 (NG2), red: PECAM, blue: Hoechst]; (Scale bar, 30 µm), and (*M*) myelinated neuronal networks: (*i*) 3D oligodendroglia distributed throughout neuronal networks (cyan: tubulin, green: tdTomato pretransfected oligodendroglia-surfaces); (Scale bar, 500 µm), (*ii*) higher magnification (cyan: tubulin, green: tdTomato pretransfected oligodendroglia); (Scale bar, 100 µm), (*iii*) myelin dye-labeled neurons and oligodendroglia (red: FluoroMyelin, cyan: tubulin, green: tdTomato pretransfected oligodendroglia), (Scale bar, 100 µm), (*iv*) visualizing via Imaris reconstructions (cyan: tubulin, Imaris surfaces of red: FluoroMyelin, green: tdTomato pretransfected oligodendroglia); (Scale bar, 100 µm), and (*v*) myelin and oligodendroglia alone (Imaris surfaces of red: FluoroMyelin, green: tdTomato pretransfected oligodendroglia); (Scale bar, 100 µm), and (*vi*) myelination of neuronal projections (green: MBP, cyan: neurofilament, blue: Hoechst); (Scale bar, 10 µm).

Current in vitro models typically coculture a maximum of three different cell types stably. To enable the coculture of all six major CNS cell types into an integral 3D tissue, we again referenced the human brain for inspiration. The brain ECM is predominantly composed of a web of polysaccharides and proteoglycans, enmeshing neuronal and glial cell types in the interstitial tissue, within which reside vascular networks ensheathed in a thin layer of BM proteins (50). These ECM components provide both structural support and important biochemical signaling factors (50, 51). We therefore undertook an approach to combine BMECs, pericytes, astrocytes, neurons, and oligodendroglia, upon validated fate specification, in hydrogel precursor solution of tunable physical and biochemical components, seed the 3D cell suspension into 48-well plates, allow the hydrogel to polymerize and the cells to form interpenetrating networks, and add iMG to the cultures on day 7, forming a 3D scaffold with integral conetworks of these six brain cell types (Fig. 1 *A*–*C*).

We hypothesized that brain-matrix proteins normally expressed in the brain at later stages of development could be harnessed to accelerate maturation. We reasoned that if we could mimic brain tissue by constructing a soft 3D hydrogel with brain-mimetic components, we could provide scaffolding to encapsulate cells at the same time as promoting enhanced brain cell phenotypes. To rapidly assay differential effects of brain-matrix components we encapsulated combinations of hyaluronic acid, heparin, reelin, tenascin-R (TenR), tenascin-C (TenC), brevican (BCN), versican (VCN), neurocan (NCN), and thrombospondin-1 (TSP1) into standard Matrigel with BMECs, pericytes, astrocytes, oligodendrocytes, and neurons (*SI Appendix*, Fig. S5*A*). The number of neuronal projections and presence of myelin basic protein (MBP) was low overall across these Matrigel-encapsulated conditions, with a small number of neuronal projections in the VCN condition (*SI Appendix*, Fig. S5 *A* and *B*). In parallel, we assayed electrical activity in these cultures via a multielectrode array (MEA). Electrical activity was low across these conditions and only occasional spikes of neurons firing were detected even after several weeks in culture, though VCN again promoted a slightly increased firing rate (*SI Appendix*, Fig. S5 *C* and *D*). Activity in Matrigel was very low, with only occasional spikes (*SI Appendix*, Fig. S5 *C* and *D*).

As Matrigel is a heterogeneous admixture of mouse tumor BM proteins with limited mechanical and biochemical tunability (52) and is rapidly degrading, as is common with protein-based biomaterials, we sought a more tunable and biochemically defined scaffolding with which to present the brain-matrix cues. Dextran is a polysaccharide biochemically similar yet distinct from hyaluronan, providing a uniquely brain-biosimilar scaffold, while still presenting a bioorthogonal backbone that can be mechanically tuned independently from biochemical cues (53). Dextran hydrogels can be fabricated at soft stiffnesses and with viscoelastic properties akin to that of brain tissue (54). We cultured miBrains in dextran-based hydrogels across mechanical stiffness conditions by altering the molar amounts of dextran macromer and cell-degradable peptide crosslinker, wherein intermediate molar ratios yielded the most robust vessel networks and neuronal projections (*SI Appendix*, Fig. S6 *A* and *B*). For the top performing formulation, we assessed the effect of degradability by introducing a PEG nondegradable crosslinker at various concentrations. Both the 100% degradable and 90% degradable hydrogel promoted neurovascular unit assembly, with integrated microvascular networks and neuronal projections (*SI Appendix*, Fig. S6 *C* and *D*) and chose to utilize the 100% degradable condition to simplify the technical scale up.

To characterize the viscoelastic properties of these dextran-based hydrogels, rheological measurements were performed on acellular hydrogels. The storage modulus (G’) was characterized in frequency dependent (*SI Appendix*, Fig. S7*A*) and strain dependent (*SI Appendix*, Fig. S7*B*) tests. G’ ranged from ~21.6 Pa for 2 mmol/L reactive groups to ~164.0 Pa for 9 mmol/L reactive groups at the lowest angular frequency. At high frequencies of ~157 rad/s, these moduli increased to ~124.0 Pa and ~398.0 Pa, respectively. A significant frequency dependence was observed, with G’ increasing at high frequencies (*SI Appendix*, Fig. S7*A*). Nonlinear elastic behavior was also observed in strain sweep tests, revealing decreasing G’ at high strain rates (*SI Appendix*, Fig. S7*B*).

With this hydrogel scaffold in place to support miBrain coculture, we revisited the hypothesis that brain matrix-specific components incorporated into a hydrogel, structurally capable of supporting their conjugation and presentation to cells, could be used to promote neuronal maturation. Screening across hydrogels incorporating HA, Heparin, Reelin, TenR, TenC, NCN, BCN, VCN, or TSP1, we first utilized a simplified coculture system of neurons and astrocytes, and monitored neuronal activity on an MEA. After 2 wk of culture, robust neuronal projections were observed across the hydrogel conditions, particularly in hydrogels incorporated with VCN, HA, TenC, and TSP1 (*SI Appendix*, Fig. S8 *A* and *B*). Strikingly, many of these matrix component conditions led to robust neuronal spiking on MEA recordings by week 2 in culture, a short onset time for iPSC-derived neurons. Representative raster plots of conditions demonstrate spiking across electrodes and bursts (*SI Appendix*, Fig. S8*C*). Of the conditions tested, VCN, Reelin, BCN, TenC, NCN, and TSP1 displayed the highest firing rates (*SI Appendix*, Fig. S8*D*).

Given these promising results, we selected a subset of these ECM components that also supported hydrogel mechanical integrity and integrated these various brain-matrix components into our engineered dextran-based hydrogel with all miBrain cell types. Robust cultures and neuronal projections formed throughout the coculture in many conditions, particularly for VCN (Fig. 1*D*). In terms of immunoreactivity of neuronal marker neurofilament, VCN and BCN led to the strongest signal intensity (Fig. 1*E*). Assessing these conditions on the MEA system in parallel, VCN and heparin displayed the highest neuronal firing rate (Fig. 1*F*). Across the many assays and experimental replicates tested, we found VCN to be a consistent top-performer. Our engineered dextran-based hydrogel incorporating VCN protein led to a reproducibly robust hydrogel scaffolding for the miBrain. Robust neurovascular phenotypes persisted in our dextran-based hydrogel through the initial testing time of 5 wk (Fig. 1*G*) and with enhanced structural integrity (Fig. 1*H*) and neuronal activity (Fig. 1*I*) compared to Matrigel. We therefore chose VCN-integrated dextran-based engineered hydrogels with RGD peptides and cell-degradable crosslinkers, terming the formulation Neuromatrix Hydrogel. Without RGD peptides, vessel networks failed to form and neurons also exhibited less spreading (*SI Appendix*, Fig. S9 *A* and *B*). Doses of VCN above that of the 50 μg/mL incorporated did not lead to any further appreciable increase in neurofilament intensity (*SI Appendix*, Fig. S10 *A* and *B*).

### 3D Immuno-Glial-Neurovascular miBrain Platform.

Constructing the miBrain by combining all six major CNS cell types together in our brain-inspired 3D matrix, we sought to characterize platform across cell- and tissue-scale biomimetic phenotypes.

While the ratio between brain cell types has been debated over the last decades, more advanced methodologies suggest the glia-to-neuron ratio is less than 1-to-1 (55), with glia in the cortex estimated to be 45 to 75% for oligodendroglia, 19 to 40% for astrocytes, and 10% or less for microglia (56). Endothelial cells are commonly estimated to comprise one-third of nonneuronal cells (57). In practice, we iteratively screened cell ratios toward supporting vascular network and neuronal network formation within an integral construct (*SI Appendix*, Fig. S11) and found that incorporating cells at ratios of 38% neurons, 11% astrocytes, 6% oligodendroglia, 5% iMG, 38% BMECs, and 2% pericytes into miBrains formed an integral 3D tissue (Fig. 1 *B* and *C*).

High cell viability was observed throughout the cultures, including in neurons (*SI Appendix*, Fig. S12 *A* and *B*). In contrast to the emergent differentiation approach of organoids, in which days in vitro commence at the beginning of the differentiation, miBrains combine already differentiated and validated cells and we refer to their days in vitro beginning with their coencapsulation together. The robust physiological features developed in the platform during the course of 1 to 2 wk are a consequence of combining predifferentiated cells in our optimized manner.

These features gave rise to 3D neurovascular units (Fig. 1*J* and Movie S1), throughout which were iMG resident immune cells (Fig. 1*K* and Movie S1), a biomimetic blood–brain barrier (BBB) (Fig. 1*L* and Movie S2), and myelin protein-positive neuronal networks (Fig. 1*M* and Movie S3), all cointegrated together in the miBrain. BMECs formed tubular networks while neurons formed networks of projections throughout the miBrain (Fig. 1 *J*, *i* and *ii*). Astrocytes throughout the miBrain closely associated with neurons (Fig. 1 *J*, *iii* and *iv*). The close proximity and cell–cell interactions between these cell types were observed at higher magnification, including between BMEC vessels and neuronal projections (Fig. 1 *J*, *v*). iMG were dispersed throughout the miBrain tissue, including in close proximity to BMEC vessels (Fig. 1 *K*, *i* and *ii*) and neurons (Fig. 1 *K*, *iii* and *iv*), together in an integrated way (Fig. 1 *K*, *v*). At the biomimetic BBB, astrocytes were proximal to BMEC microvessels throughout the miBrain (Fig. 1 *L*, *i*–*iii*), with BMECs expressing tight junctions (Fig. 1 *L*, *iv*), astrocytes expressing canonical transporter aquaporin-4 (Fig. 1 *L*, *v*), and pericytes bordering the vessels (Fig. 1 *L*, *vi*). Oligodendroglia closely associated with neurons throughout the miBrain (Fig. 1 *M*, *i* and *ii*) and were surrounded by FluoroMyelin-positive signal, lining the neuronal networks (Fig. 1 *M*, *iii* and *iv*), which were also positive for MBP (Fig. 1 *M*, *vi*).

### Characterization of Neuronal Phenotypes in miBrain.

**Fig. 2.**
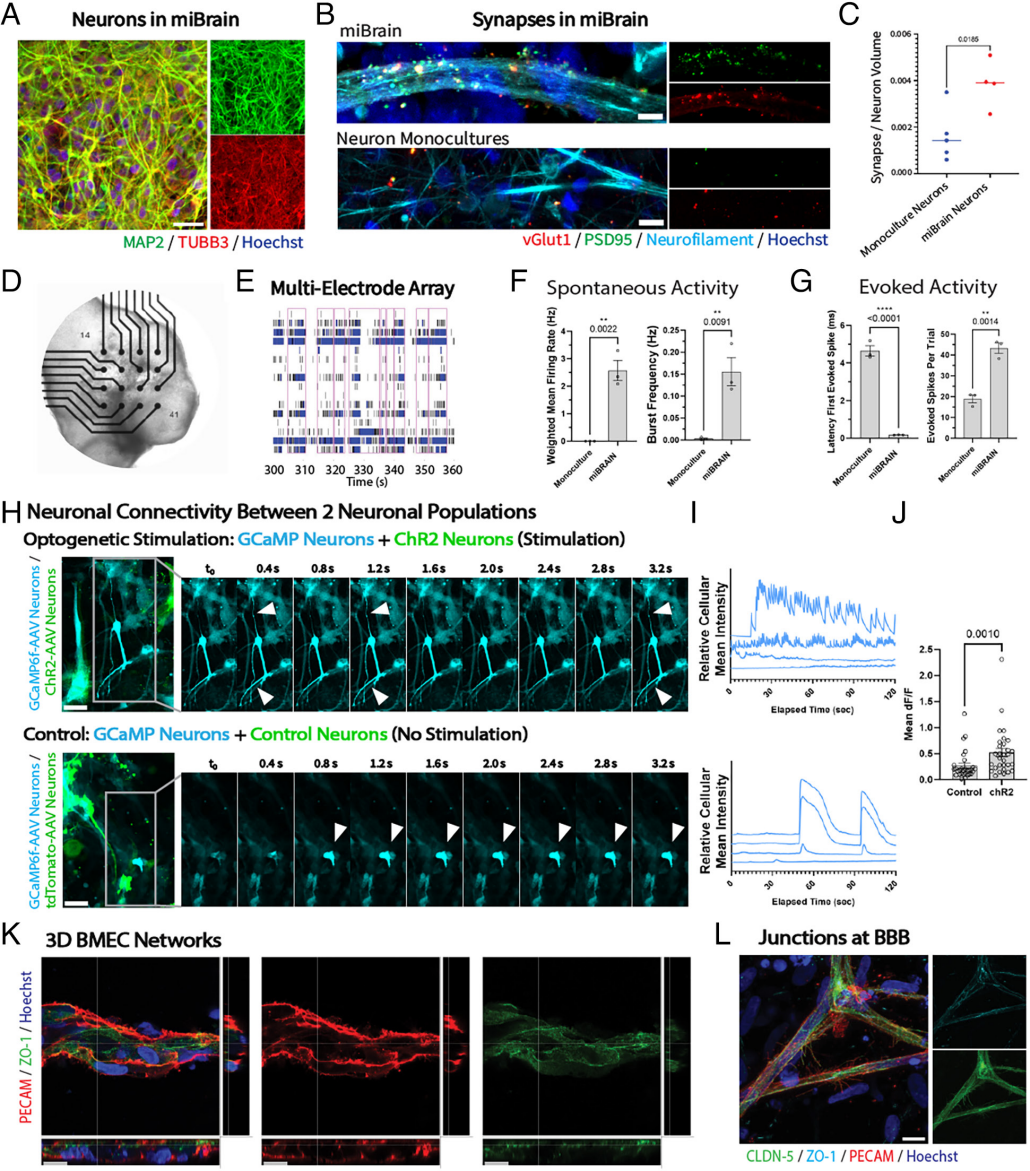
Neuronal and vascular phenotypes enabled by the miBrain. (*A*) Characterization of neurons integrated into miBrain immunohistochemistry [green: microtubule-associated protein 2 (MAP2), red: TUBB3, blue: Hoechst]; (Scale bar, 30 µm), (*B*) synapses in miBrain (*Top*) versus neuronal monocultures (*Bottom*) [red: vesicular glutamate transporter 1 (vGlut1), green: postsynaptic density protein 95 (PSD95), cyan: neurofilament, blue: Hoechst]; (Scale bar, 5 µm), (*C*) quantification of synapses (number of PSD95/synapsin colocalized puncta per volume neurofilament; n = 4, Student’s *t* test *P* = 0.0185), (*D*) macroscopic image of miBrain on MEA, (*E*) example raster plot miBrain at week 10, (*F*) characterization of spontaneous activity: (*Left*) weighted mean firing rate, (*Right*) burst frequency (mean, SEM; statistical analysis as unpaired *t* test), (*G*) evoked activity: (*Left*) latency after stimulation, (*Right*) number of evoked spikes per trial (mean, SEM; statistical analysis as unpaired *t* test), (*H*) assessing neuronal connectivity between two neuronal populations in miBrain for: (*Top*) channelrhodopsin-2 (ChR2)-mCherry for optogenetic stimulation (green: ChR2-mCherry-neurons), (*Bottom*) control tdTomato for the no stimulation condition (green: tdTomato-neurons) and (*Right*) sequential images of calcium transients imaged under blue light (cyan: GCaMP6f-neurons), (Scale bar, 30 µm), (*I*) corresponding example spike traces (*J*) quantification of calcium events (mean dF/F per calcium trace per neuron recorded (n = 3 miBrains/group, repeated in three independent trials; statistical analysis via Student *t* test *P* = 0.0010), (*K*) lumenized 3D BMEC vessels (red: PECAM, green: ZO-1, blue: Hoechst); (Scale bar, 15 µm), and (*L*) claudin-5 (CLDN-5) tight junctions along vessels (green: CLDN-5, cyan: ZO-1, red: PECAM, blue: Hoechst); (Scale bar, 100 µm).

Evaluating neuronal markers via immunohistochemistry, miBrain-neurons were found to be largely MAP2-positive (Fig. 2*A*) and express synaptic proteins, such as the presynaptic vGlut1 and postsynaptic PSD95, and with a greater density than that of monocultured neurons (Fig. 2 *B* and *C*). To assess neuronal activity in the miBrain, we differentiated neurons from an iPSC line stably expressing tdTomato and GCaMP3. GCaMP-neurons integrated into miBrains formed projections and displayed expected neuronal morphologies. Live monitoring calcium transients in GCaMP-neurons, spikes of neuronal activity were recorded over time (*SI Appendix*, Fig. S13*A*). Neurons in miBrains exhibited robust spikes of activity and displayed enhanced calcium transients compared to monocultured neurons (*SI Appendix*, Fig. S13 *B* and *C*). In parallel, given the complementarity of the methods in terms of resolution and sample size, we evaluated neuronal activity on the MEA system (Fig. 2*D*). Though miBrains are a 3D tissue and this method only captures activity toward the bottom surface, we were able to detect robust electrical activity (Fig. 2*E*). miBrains displayed enhanced spontaneous neuronal activity in terms of spike rate and burst rate (Fig. 2*F*) and promoted stimulus responsiveness, decreasing the latency time to spiking upon electrical stimulation and increasing the subsequently evoked spikes (Fig. 2*G*).

To evaluate the connectivity between neurons within the miBrain, we incorporated two populations of neurons, between which activity was assessed with and without optogenetic stimulation. In both sets of miBrains, one population of neurons was pretransfected with GCaMP6f via AAV. In one set of miBrains, a second neuron population was pretransfected with Channelrhodopsin-2 (ChR2)-mCherry, while in the other miBrain set, the second neuron population was instead pretransfected with a control AAV with tdTomato. This approach enables testing the responsiveness of one neuronal population to the heightened activity in another population, while visualizing these distinct neuronal populations. The excitation blue light for recording GCaMP6f-neurons, activates the ChR2-neurons in the stimulation condition and not the control neurons in the no stimulation condition (Fig. 2*H*). Increased spiking was observed in the GCaMP6f-neurons when cocultured with the ChR2-neurons (Fig. 2*I*), with increased overall calcium transients, evidencing the connectivity between neuronal populations (Fig. 2*J*).

Assessing the responsiveness of miBrain-neurons to canonical channel modulators, we tested a panel of pharmacological agents: voltage-gated sodium channel inhibitor TTX, AMPA competitive antagonist NBQX, NMDA receptor antagonist MK801, glutamate, which agonizes metabotropic glutamate receptors, and KCl, which triggers neuronal depolarization. Modulators were applied in increasing doses as neuronal activity was recorded on an MEA system. Doses of 8 nM TTX, 50 µM NBQX, and 50 µM MK801 decreased neuronal activity, assessed via mean firing rate, while 10 mM Glutamate and 1 mM KCl increased neuronal activity (*SI Appendix*, Fig. S13*D*). Characterizing miBrain-neurons via whole-cell recordings, action potentials were recorded via current clamp (*SI Appendix*, Fig. S13*E*) and inward and outward currents were recorded via voltage clamp (*SI Appendix*, Fig. S13*F*). While establishing a patch-clamp within the 3D hydrogel required a nonideal disruption of tissue integrity, we found miBrain-neurons displayed electrophysiological properties across these metrics, as well as for resting membrane potential (*SI Appendix*, Fig. S13*G*) and input resistance (*SI Appendix*, Fig. S13*H*) across samples (*SI Appendix*, Fig. S13*I*).

To evaluate the transcriptomic changes that neurons undergo in the miBrain, we performed RNAseq (*SI Appendix*, Fig. S14 *A*–*C*). Comparing monoculture- and miBrain-neurons, several neuronal identity genes were evidently upregulated in the miBrain condition (such as *SCN1A* and *CACNA1C;* *SI Appendix*, Fig. S14*D*) and genes associated with neuronal precursor cells were downregulated (like *SOX11* and *PAX6;* *SI Appendix*, Fig. S14*E*). These neuronal signatures provide evidence of strong neuronal phenotypes in the miBrain. Among the differentially expressed genes (DEGs), genes associated with modulation of synaptic transmission, axon fasciculation, and astrocyte development are among those most strongly upregulated in miBrain-neurons (*SI Appendix*, Fig. S14 *F* and *G*). Conversely, neuronal stem cell population maintenance and neuroepithelial cell differentiation pathways are among those most strongly downregulated (*SI Appendix*, Fig. S14 *F* and *G*). In an effort to compare these datasets to human in vivo neurons, we correlated sequenced transcriptomes for miBrain- and monoculture-neurons to all the genes of excitatory neurons in the prefrontal cortex from non-AD adult decedents (58). When correlating human in vivo excitatory neurons to miBrain-neurons and to monoculture-neurons, differences were not observed when sampling over all genes (*SI Appendix*, Fig. S14 *H*, *i*), however, miBrain-neurons correlated more strongly than monoculture-neurons across pathways such as neuron differentiation (*SI Appendix*, Fig. S14 *H*, *ii*) and axon ensheathment (*SI Appendix*, Fig. S14 *H*, *iii*). Comparing DEGs upregulated between these datasets, we found that miBrain-neurons resulted in 287 unique DEGs when compared to in vivo neurons, while larger unique differences were identified in monoculture-neurons and in vivo with 328 unique DEGs (*SI Appendix*, Fig. S14 *I*, *i*). For downregulated DEGs, miBrain-neurons had 350 unique DEGs versus in vivo, while monoculture-neurons had 344 (*SI Appendix*, Fig. S14 *I*, *ii*).

Given that positive effects on neuronal maturation can be observed in less complex systems as well, for example, coculture with astrocytes (59), we asked whether the observed changes in signatures were attributable to neuron-astrocyte coculture. We compared our miBrain-neuron and monocultured-neuron RNAseq to a dataset of neuron-astrocyte coculture that utilized the same differentiation protocols for astrocytes and NGN2-induced neurons (59). Comparing neurons from these coculture systems, a weak positive correlation was observed with an RMSE value of 1.084 (*SI Appendix*, Fig. S15 *A*, *i*). Genes like *TGM2, COL14A1,* and *ITGB4* were found to be enriched in both coculture conditions, while genes like *DCX, CNTFR,* and *SYT4* were downregulated in both systems (*SI Appendix*, Fig. S15 *A*, *i*). Probing the primary changes in each of these systems, we found there was not sufficient statistical power to identify DEGs in neurons from neuron-astrocyte cultures (*SI Appendix*, Fig. S15 *B*, *i*), while a multitude were observed in miBrain-neurons (*SI Appendix*, Fig. S15 *C*, *i*). We further examined these expression differences on the basis of neuron relevant gene programs, finding miBrain-neurons display a stronger upregulation of neuronal identity-associated genes *NTRK2* and *NEFL* compared to neurons cocultured with astrocytes, among other differences (*SI Appendix*, Fig. S15*D*). Thus, the miBrain leads to gene expression changes distinct from those of neuron-astrocyte coculture conditions.

### Characterization of BBB Integrated in miBrain.

An ideal brain model would contain an integrated, 3D BBB expressing the physiologically relevant transporters and receptors present in the human BBB, surrounded by the diversity of cell types that play key roles in reinforcing the barrier and contributing to disease pathogenesis. BMECs formed networks fully integrated into the miBrain and extending throughout the engineered tissue mimic (Fig. 1*C*). These microvascular networks formed 3D lumenized tubules expressing platelet endothelial cell adhesion molecule (PECAM) and tight junction markers ZO-1 (Fig. 2*K*) and CLDN-5 (Fig. 2*L*) and adhesion protein VE-CAD (*SI Appendix*, Fig. S16*A*). We further confirmed the presence of tight junctions through transmission electron microscopy (*SI Appendix*, Fig. S16*D*).

To assess neurovascular integration of BMEC vessels and neurons we undertook an analogous approach to that used in assessing neuronal connectivity (Fig. 2 *H*–*J*), in which we constructed miBrains with BMECs pretransfected with GCaMP6f and either ChR2-mCherry-neurons or control tdTomato-neurons (*SI Appendix*, Fig. S16*E*). BMECs in miBrains with control tdTomato-neurons displayed endogenous calcium transients. These BMEC calcium transients were more frequent in miBrains with optogenetically stimulated ChR2-mCherry neurons (*SI Appendix*, Fig. S16 *F* and *G*). This indicates that BMECs in miBrains are responsive to enhanced neuronal firing and provides evidence of active neurovascular units in the miBrain.

**Fig. 3.**
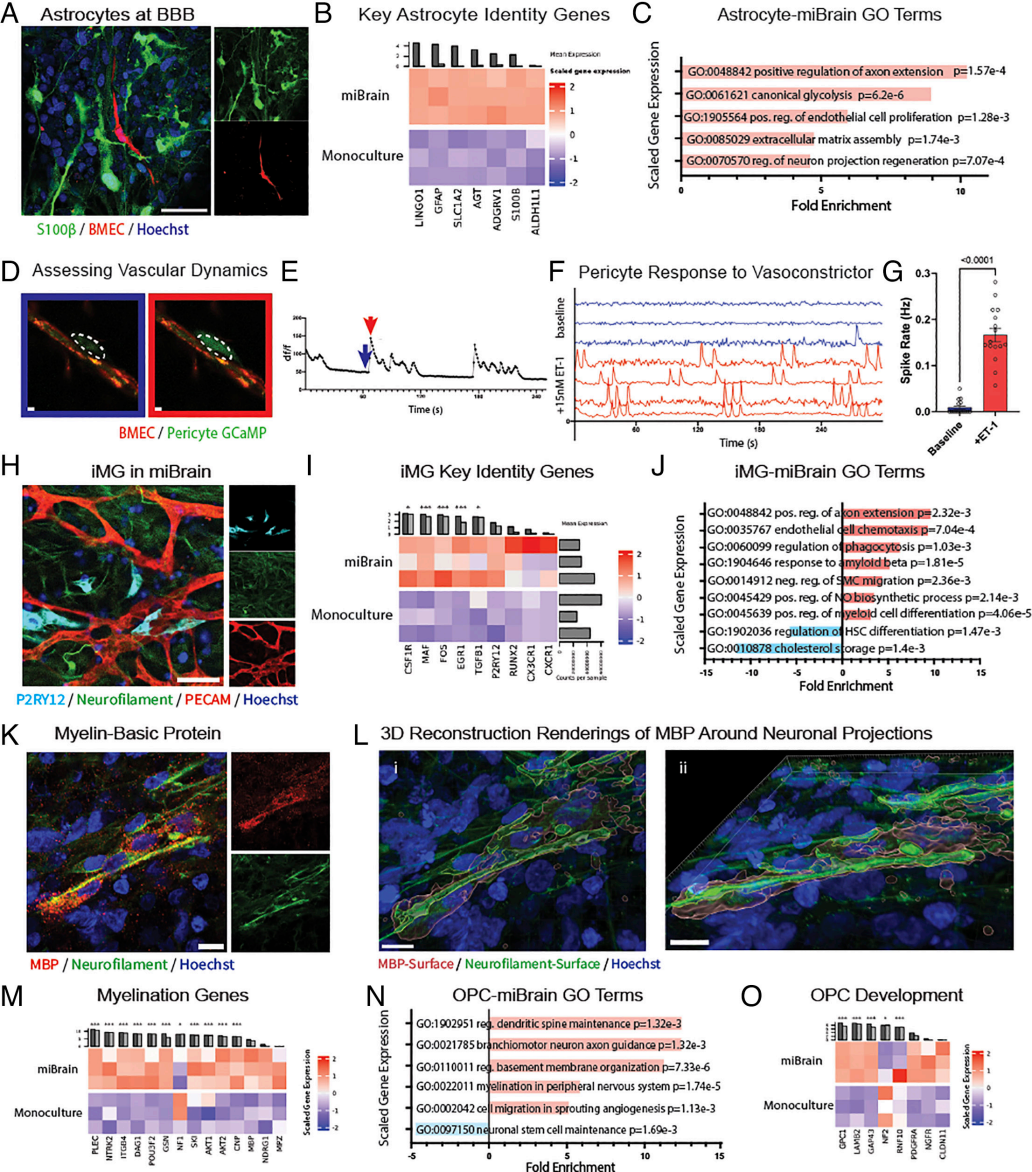
Glial phenotypes enabled by the miBrain. (*A*) immunohistochemistry for astrocyte marker S100 calcium-binding protein B (S100β) (green: S100β, red: mCherry-BMEC, blue: Hoechst); (Scale bar, 50 µm), (*B*) expression of key in vivo genes in RNAseq from astrocytes isolated from miBrains versus monoculture (TMM-normalized, scaled expression), (*C*) gene ontology analysis of biological pathways upregulated in miBrain-cultured astrocytes based on significantly upregulated and downregulated DEGs (FDR *P*-value < 0.05), (*D*) calcium imaging of pericytes (*Left*, blue) before and (*Right*, red) during a signaling event; (Scale bar, 10 µm), (*E*) example traces of calcium transients, (*F*) spike traces (*Top*, blue) with and (*Bottom*, red) without application of vasoconstrictor ET-1, (*G*) quantification of rate of calcium events (mean, SEM; statistical analysis via paired *t* test), (*H*) miBrain-iMG immunoreactivity to purinergic receptor P2RY12 (cyan: P2RY12, green: neurofilament, red: PECAM, blue: Hoechst); (Scale bar, 50 µm), (*I*) expression of key in vivo genes in RNAseq of miBrain-iMG versus monoculture (TMM-normalized, scaled expression; *P < 0.05, **P < 0.01, ***P < 0.001), (*J*) gene ontology analysis for biological pathways significantly altered in miBrain-cultured iMG, (*K*) characterization of myelination via immunohistochemistry (red: MBP, green: neurofilament, blue: Hoechst); (Scale bar, 10 µm), (*L*) 3D reconstructions of MBP-positive regions around neuronal projections with (*i*) *Top* and (*ii*) side views (red: MBP-Surface, green: neurofilament-Surface, blue: Hoechst); (Scale bar, 10 µm), (*M*) expression of key myelination genes in RNAseq of miBrain-oligodendroglia versus monoculture (TMM-normalized, scaled expression), (*N*) gene ontology analysis of biological pathways significantly altered in miBrain-cultured oligodendroglia based on significantly upregulated and downregulated DEGs, and (*O*) expression of key genes associated with OPC development in RNAseq of miBrain-oligodendroglia versus monoculture (TMM-normalized, scaled expression).

Interacting with the brain microvasculature and forming the parenchymal niche of the BBB are astrocytes—important glial cells regulating vascular function as well as neuronal processes (60). iPSC-derived astrocytes, however, often have limited maturity with low expression levels of canonical fate markers. In the miBrain, astrocytes expressed canonical marker GFAP and water channel AQP4 and extended endfeet onto the vasculature (Fig. 1 *L*, *v*). Further, miBrains harbored positive immunoreactivity for gap junction marker connexin-43 (Cx43) around the microvasculature (*SI Appendix*, Fig. S16*B*) and canonical astrocyte marker S100β (Fig. 3*A*).

To characterize miBrain-astrocytes and better understand the effect of the miBrain niche on astrocyte signatures, we sequenced astrocytes (*SI Appendix*, Fig. S17 *A* and *B*). In the miBrain, astrocytes displayed a striking enrichment of identity genes like *S100β, SLC1A2,* and *ALDH1L1* (Fig. 3*B*), enhanced S100β (*SI Appendix*, Fig. S17 *H* and *I*) and GFAP (*SI Appendix*, Fig. S17 *J* and *K*) immunoreactivity, and a strong upregulation of a wide variety of genes involved in synaptic modulation and neuronal signaling (*SI Appendix*, Fig. S17*C*) as well as vascular regulation (*SI Appendix*, Fig. S17*D*). This points to the recapitulation of both barrier (including via *ROBO2* and *SEMA6A*) and neuronal process regulation (including via *NRP1*) in miBrain-astrocytes, among other DEGs (*SI Appendix*, Fig. S17*E*). Consonantly, gene ontology analysis revealed a strong upregulation in biological pathways associated with neuronal processes and endothelial cell processes, as well as in ECM assembly (Fig. 3*C*). When these RNAseq datasets were compared to human in vivo astrocyte via a snRNAseq dataset from prefrontal cortex tissues of non-AD decedents (58), miBrain-astrocytes correlated more strongly than monoculture-astrocytes (*SI Appendix*, Fig. S17*F*). Congruently, miBrain-astrocytes had 272 uniquely upregulated DEGs compared to in vivo, while more unique differences were observed between monoculture-astrocytes and in vivo with 387 DEGs (*SI Appendix*, Fig. S17 *G*, *i*). For downregulated DEGs, miBrain-astrocytes had 271 unique DEGs versus in vivo, whereas monoculture-astrocytes had more with 452 (*SI Appendix*, Fig. S17 *G*, *ii*).

To test whether the changes in gene expression in astrocytes could be explained by the coculture with neurons, we compared our miBrain-astrocyte RNAseq to the dataset of neuron-astrocyte coculture (59) along with the respective monoculture samples. Between these cocultures, only a weak correlation was observed, with an RMSE value of 1.275 (*SI Appendix*, Fig. S15 *A*, *ii*). Genes like *SPARCL1* and *SULF1* were found to be enriched in both coculture conditions, while genes like *ANKRD1, OXTR,* and *F3* were downregulated in both systems (*SI Appendix*, Fig. S15 *A*, *ii*). Probing the primary changes in each of these systems, we found that astrocytes from both the neuron-astrocyte cultures (*SI Appendix*, Fig. S15 *B*, *ii*) and miBrain (*SI Appendix*, Fig. S15 *C*, *ii*) displayed a large number of significant DEGs. The relative change in expression in astrocytes in the miBrain compared to neuron-astrocyte coculture is distinct for many genes associated with regulation of neuronal projection regeneration, like *THY1* and *SPP1,* axon extension, like *CXCL12* and *VEGFA,* canonical glycolysis, synapse organization, and regulation of angiogenesis (*SI Appendix*, Fig. S15*E*).

While endothelial cells are the primary regulators of BBB permeability and selectivity, pericytes in the human brain are coupled to capillaries and exercise key functions in BBB maintenance, transport regulation, and cerebrovascular blood flow (61). Correspondingly, in the miBrain pericytes localized to the outer lumen of the BMEC networks (*SI Appendix*, Fig. S16 *H*–*J*). As pericyte calcium increases in response to voltage-gated calcium channels and promotes contraction (61), we sought to assess their functional coupling to the microvasculature. We constructed miBrains with pericytes stably expressing GCaMP6 and monitored their calcium transients in miBrains (Fig. 3 *D* and *E*). We stimulated vessels with potent vasoconstrictor endothelin-1 (ET-1), known to induce a prolonged increase in blood pressure in vivo, and monitored pericyte calcium signaling as a proxy for pericyte contractile activity. ET-1 stimulation induced network-wide pericyte calcium transients and this elevated calcium activity persisted for minutes (Fig. 3 *F* and *G*), indicating a robust pericyte response to vasomodulation.

BMECs, pericytes, and astrocytes together form the BBB, which is further modulated and influenced by the other cell types in the brain. To assess whether miBrain-BBBs respond to BBB modulators known to promote or reduce barrier function, we tested a panel of pharmacological agents. miBrains treated with thrombin exhibited decreased average vessel area and diminished total vessel area, while those treated with hydrocortisone displayed increased vessel area metrics (*SI Appendix*, Fig. S18 *A*–*C*). Dose curves of LPS and of TNF-α also led to smaller vessel areas and doses of 100 and 1,000 ng/mL TNF-α also led to an overall decrease in total miBrain area (*SI Appendix*, Fig. S18 *A*–*C*). As ZO-1 is an important barrier marker, we assessed its expression across treatment conditions via immunohistochemistry. Consistent with expected results, we found that hydrocortisone enhanced vascular ZO-1 signal intensity while the other modulators decreased ZO-1 (*SI Appendix*, Fig. S18 *D* and *E*). To further assess changes in barrier function, we measured the membrane resistance of a BMEC monolayer with and without miBrain seeding proximal to the BMECs on a transwell system. BMECs with miBrains displayed increased resistance (*SI Appendix*, Fig. S16*C*). Interestingly, comparing miBrains constructed with cells derived from an isogenic iPSC line CRISPR-edited to *APOE4/4* AD risk, BMEC monolayers seeded with these *APOE4/4* miBrains had decreased barrier function compared to *APOE3/3* miBrains as assessed via TEER on a transwell system (*SI Appendix*, Fig. S16*C*).

### Characterization of Glial Phenotypes in miBrain.

Microglia and oligodendrocytes regulate multiple processes in the brain, with key roles in neuronal health and function, while also contributing to vascular regulation and other physiological functions (62, 63).

While neuroinflammatory processes are an important component of neurodegeneration, the culture of microglia in vitro in homeostatic conditions has been challenging (64). Through immunohistochemistry analysis, we observed that iMG incorporated into the miBrain integrated and extended processes in close association with the neurons and vasculature (Fig. 3*H*). miBrain-iMG displayed immunoreactivity to purinergic receptor P2RY12, involved in motility and associated with homeostatic processes (Fig. 3*H*).

Assessing the transcriptional signatures of miBrain-iMGs, RNA isolated from iMG (*SI Appendix*, Fig. S19 *A* and *B*) revealed the miBrain leads to upregulated P2RY12 in iMG (*SI Appendix*, Fig. S19*C*), which is also evident via flow cytometry assessment on the protein level (*SI Appendix*, Fig. S19*D*). Between miBrain- and monoculture-iMG, a number of DEGs were identified (*SI Appendix*, Fig. S19 *E* and *F*). Importantly, key microglia identity genes were upregulated in the miBrain-iMG, including *CX3CR1, EGR1,* and *CSF1R* (Fig. 3*I* and *SI Appendix*, Fig. S19*E*). Further, miBrain-iMG upregulated genes associated with neuronal signaling and endothelial cell processes (Fig. 3*J*). Comparing miBrain- and monoculture-iMG to a human in vivo dataset of adult non-AD prefrontal cortex tissue (58), we found that miBrain-iMG correlated more strongly with human microglia (*SI Appendix*, Fig. S19*G*). To better understand what pathways could contribute to this enrichment of biomimetic signatures, we further compared miBrain- and monoculture-iMG to human in vivo microglial states our laboratory has identified (65). miBrain-iMG correlated more strongly to the homeostatic state than monoculture-iMG (*SI Appendix*, Fig. S19 *H*, *i*). Across the various states (*SI Appendix*, Fig. S19*H*), the homeostatic state was the only one found to exhibit a statistically significant difference, though the neuronal surveillance state signature had the next highest *P*-value, increased in miBrain-iMG (*P* = 0.06; *SI Appendix*, Fig. S19 *H*, *i*). These results indicate that the miBrain niche promotes enhanced microglia signatures and provides a system in which their multifaceted interactions with vascular and neuronal components can be further probed in high-resolution and high throughput. This is significant, given the importance of establishing a homeostatic baseline for inflammatory assays. Comparing downregulated DEGs across datasets, miBrain-iMG had 328 unique DEGs versus in vivo, while monoculture-iMG differences were greater with 461 (*SI Appendix*, Fig. S19 *I*, *i*). For upregulated DEGs, miBrain-iMG had 404 unique DEGs comparing to in vivo, whereas monoculture-iMG yielded 405 (*SI Appendix*, Fig. S19 *I*, *ii*).

Given the important role of microglia in a variety of processes, including immune surveillance, BBB function (66), and neuronal activity modulation (67, 68), we sought to functionally monitor microglia dynamics in the miBrain in real-time. We observed motile iMG migrating through the miBrain, including in close proximity to the microvasculature (*SI Appendix*, Fig. S19*J*). Dynamic iMG were also observed closely associating with neurons (*SI Appendix*, Fig. S19 *K*, *i*) and physically interacting with neuronal projections (*SI Appendix*, Fig. S19 *K*, *ii*).

Oligodendrocytes in vivo are the myelinating brain cell type that engage with neurons. Assessing oligodendroglia phenotypes in the miBrain, we detected positive MBP immunoreactivity (Fig. 3*K*) around neuronal projections (Fig. 3*L* and *SI Appendix*, Fig. S20*A*). Analyzing the transcriptional signatures of oligodendroglia (*SI Appendix*, Fig. S20 *B*–*E*), we found miBrain-oligodendroglia displayed an upregulation of genes associated with myelination (Fig. 3*M*), biological pathways associated with dendritic spine maintenance and neuron axon guidance (Fig. 3*N*), including genes suggestive of neuronal interactions (such as *NDRG4, KIF1A,* and *SNAP25*; *SI Appendix*, Fig. S20*F*) and of vascular interactions (such as *VEGFA, KDR, FLT4,* and *NRP1*; *SI Appendix*, Fig. S20*G*). Concomitantly, pathways associated with maintenance of neuronal stem cells (Fig. 3*N*) were strongly downregulated while genes associated with maturation of OPCs were promoted (including *GPC1* and *PDGFRα*; Fig. 3*O*). miBrain-oligodendroglia displayed additional DEGs compared to monocultured oligodendroglia (*SI Appendix*, Fig. S20 *E* and *H*), including 179 uniquely downregulated (*SI Appendix*, Fig. S20 *I*, *i*) and 195 uniquely upregulated DEGs (*SI Appendix*, Fig. S20 *I*, *ii*). Comparing these datasets further to human in vivo oligodendroglia, miBrain-oligodendroglia yielded 277 unique DEGs, while monoculture-oligodendroglia had 281 (*SI Appendix*, Fig. S20 *I*, *i*). As for upregulated DEGs, miBrain-oligodendroglia had 288 unique DEGs compared to in vivo, whereas monoculture-oligodendroglia had 367 (*SI Appendix*, Fig. S20 *I*, *ii*).

Overall, miBrain-oligodendroglia correlated more strongly to human in vivo oligodendroglia signatures identified via the scRNAseq of prefrontal cortex, non-AD decedent brain tissue (58) (*SI Appendix*, Fig. S20*J*). Electron microscopy on miBrains further revealed ringed structures indicative of oligodendrocytes wrapping around neuronal axons (*SI Appendix*, Fig. S20*K*).

### Harnessing miBrain to Investigate Functional Consequences of *APOE4* Astrocytes.

The *E4* allele of the gene encoding lipid transport protein apolipoprotein E (APOE) strongly increases risk for developing late-onset AD and decreases age of disease onset compared to the *E3* allele (69). Astrocytes are a primary producer of APOE, though the extent of *APOE4* astrocytes’ role in AD pathogenesis remains poorly understood (70–72). We therefore sought to investigate the effect of *APOE4* astrocytes harnessing the miBrain.

**Fig. 4.**
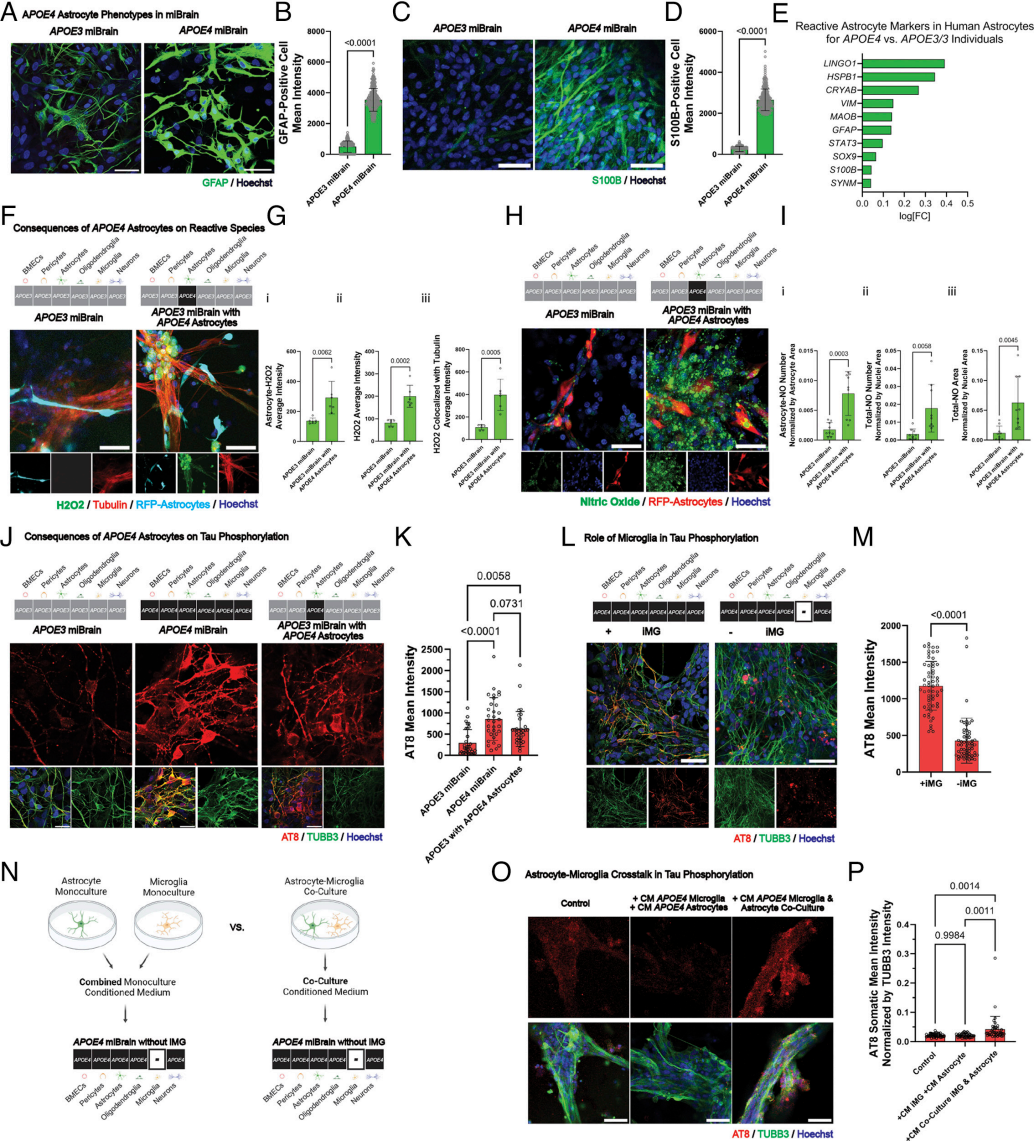
miBrain reveals *APOE4* astrocytes promote tau phosphorylation via crosstalk with microglia. (*A*) GFAP immunoreactivity in *APOE3* (*Left*) versus *APOE4* (*Right*) miBrains (green: GFAP, blue: Hoechst); (Scale bar, 50 µm), (*B*) quantification of GFAP (mean intensity normalized by nuclei area; n = 3 samples, images analyzed from n = 6 max projections; statistical analysis by the *t* test), (*C*) S100β immunoreactivity in *APOE3* (*Left*) versus *APOE4* (*Right*) miBrains (green: S100b, blue: Hoechst); (Scale bar, 50 µm), (*D*) quantification of S100β (mean intensity normalized by nuclei area; n = 3 samples, images analyzed from n = 6 max projections; statistical analysis by the *t* test), (*E*) DEGs in human astrocytes from snRNAseq dataset (22) of *APOE3/4* and *APOE4/4* versus *APOE3/3* individuals (positive log[FC] is upregulated in *APOE4*) for reactive astrocyte genes, (*F*) hydrogen peroxide (H2O2) in *APOE3* (*Left*) versus *APOE4* (*Right*) astrocytes incorporated into otherwise-*APOE3* miBrains (green: H2O2, red: tubulin, cyan: RFP-astrocytes, blue: Hoechst); (Scale bar, 50 µm), (*G*) quantification of (*i*) mean H2O2 intensity colocalized with astrocytes, (*ii*) mean H2O2 intensity in positive regions, and (*iii*) mean H2O2 intensity colocalized with tubulin-positive regions (n = 3 replicates, images from n = 6 max projections; statistical analysis via *t* test; conducted in three independent trials), (*H*) NO in miBrains with *APOE3* (*Left*) versus *APOE4* (*Right*) astrocytes incorporated into otherwise-*APOE3* miBrains (green: NO, red: RFP-astrocytes, blue: Hoechst); (Scale bar, 50 µm), (*I*) quantification of: (*i*) NO droplets colocalized with astrocytes normalized by astrocyte area, (*ii*) number of NO droplets normalized by nuclei area, and (*iii*) total area of NO segmented particles normalized by nuclei area (n = 3 replicates, images from n = 9 max projections; statistical analysis via *t* test; conducted in three independent trials), (*J*) tau phosphorylation in *APOE3* (*Left*), *APOE4* (*Middle*), and *APOE3* miBrains with *APOE4* astrocytes (*Right*) treated with 20 nM exogenous Aβ 1-42 (red: AT8, green: TUBB3, blue: Hoechst); (Scale bar, 30 µm), (*K*) quantification of somatic tau phosphorylation in TUBB3-positive neurons (mean AT8 intensity per cell; n = 3 replicates, images from n = 12 fields of view; statistical analysis via ANOVA; repeated for three independent trials), (*L*) tau phosphorylation in *APOE4* miBrains with iMG (*Left*) versus without iMG (*Right*) treated with exogenous Aβ 1-42 (red: AT8, green: TUBB3, blue: Hoechst); (Scale bar, 50 µm), (*M*) quantification of somatic tau phosphorylation in TUBB3-positive neurons (mean AT8 intensity per cell; n = 3 replicates, images from n = 12 fields of view; statistical analysis via *t* test; repeated for three independent trials), (*N*) schematic illustrating experiments testing the effect of *APOE4* microglia-astrocyte crosstalk by applying CM from astrocyte monocultures and microglia monocultures versus microglia-astrocyte coculture, (*O*) tau phosphorylation in *APOE4* miBrains treated with (*Left*) media only, (*Middle*) monoculture CM, (*Right*) coculture CM, coadministered with exogenous Aβ 1-42 (red: AT8, green: TUBB3, blue: Hoechst); (Scale bar, 50 µm), and (*P*) quantification of somatic tau phosphorylation in TUBB3-positive neurons (mean AT8 intensity normalized by mean TUBB3 intensity per cell; n = 3 replicates, images from n = 12 fields of view; statistical analysis via ANOVA).

We constructed miBrains from isogenic sets of *APOE3/3* and *APOE4/4* iPSCs (32) and evaluated GFAP signal, given the increasing utilization of GFAP as a biomarker for AD (73, 74). We found that *APOE4* miBrains displayed increased GFAP intensity (Fig. 4 *A* and *B*), along with that of other reactive astrocyte markers, STAT3 (*SI Appendix*, Fig. S21 *A* and *B*) and C3 (*SI Appendix*, Fig. S21 *C* and *D*). Concomitantly, higher immunoreactivity to S100β, associated with neurodegenerative phenotypes (75, 76), was observed in *APOE4* (Fig. 4 *C* and *D*). These differences were not appreciable in *APOE4* versus *APOE3* astrocytes in monoculture (32) (*SI Appendix*, Fig. S22*A*), but only captured in the multicellular environment of the miBrain. miBrain-astrocytes are also responsive to insult, assessed via treatment with LPS, in which S100β (*SI Appendix*, Fig. S22 *B* and *C*) and GFAP (*SI Appendix*, Fig. S22 *D* and *E*) are further elevated compared to no-treatment controls. To evaluate whether the results mirror expression of astrocytes in human brains, we analyzed a single-nucleus transcriptomic dataset for reactive astrocyte genes for *APOE4/4* and *APOE3/4* individuals versus *APOE3/3* individuals that our laboratory recently generated (22). We found a broadly increased expression of reactive astrocyte genes in *APOE4* carriers compared to noncarriers, including for *GFAP, S100β,* and *VIM* (Fig. 4*E*). Among the panel of reactive astrocyte genes evaluated, the most strongly upregulated were *LINGO1, HSPB1,* and *CRYAB* (Fig. 4*E*). We therefore sought to assess the functional consequences of the increased reactive phenotype of *APOE4* astrocytes.

While astrocyte genotype cannot be decoupled from that of the other cell types in organoids due to their inherent coemergent differentiation, in the miBrain platform it can. Therefore, to assess astrocyte-specific contributions to *APOE4* pathogenesis we harnessed this advantageous miBrain capability to generate miBrains with *APOE3* or *APOE4* astrocytes.

Given that astrocytes play critical roles in regulating oxidative stress (77) and that reactive astrocytes can aggravate neuronal damage (70) and AD-associated pathological hallmarks, including via hydrogen peroxide production (71), we examined reactive species in the miBrain. We detected higher levels of hydrogen peroxide colocalizing with astrocytes in the miBrains with *APOE4* astrocytes compared to the all-*APOE3* condition (Fig. 4 *F* and *G*, *i*). Interestingly, increased hydrogen peroxide was also observed overall in the miBrains with *APOE4* astrocytes (Fig. 4 *F* and *G*, *ii*), including in neuron-colocalizing regions (Fig. 4 *G*, *iii*). Strikingly, intercellular droplets of nitric oxide (NO) were observed in miBrains with *APOE4* astrocytes (Fig. 4*H*). Between *APOE4-* versus *APOE3-*astrocyte miBrain conditions, more NO was observed colocalizing with *APOE4* astrocytes (Fig. 4 *H* and *I*, *i*) and in the overall for the *APOE4* astrocyte-containing miBrain (Fig. 4 *H* and *I*, *ii* and *iii*). We similarly evaluated *APOE3* miBrains with *APOE4* versus *APOE3* astrocytes with a live indicator for peroxynitrite (*SI Appendix*, Fig. S22 *F*, *i*), where a significant increase in peroxynitrite species was observed not in astrocytes, but overall and colocalizing with neurons (*SI Appendix*, Fig. S22 *F*, *ii*–*iv*). Together, these results provide evidence that not only do *APOE4* astrocytes harbor an increased proclivity to form hydrogen peroxide and NO species, but that they promote the accumulation of these species in neurons and other cells, which could contribute to *APOE4* neuropathologies. As *APOE4* miBrains with *APOE3* astrocytes instead of *APOE4* still exhibit reactive species, it appears that astrocytes are not the only *APOE4* cell type to contribute to this reactive phenotype (*SI Appendix*, Fig. S23), however our data suggest that they have a significant role in promoting it.

Imbalanced oxidative stress and free radicals contribute to lysosomal dysfunction, which can exacerbate the cellular damage implicated in AD and other neurodegenerative diseases (72, 78). Evaluating lysosomal processes in our snRNAseq dataset of *APOE4* carriers and noncarriers (22), we found differential gene expression in *APOE4* for genes associated with regulation of lysosomal catabolism, including *LRP1, LDLR,* and *ATP13A2* (*SI Appendix*, Fig. S22*G*). Many related genes participate in regulation of oxidative stress-induced cell death, a pathway displaying upregulation of genes like *HSPB1, NFE2L2,* and *SOD1* and downregulation of *PRKN, PDE8A,* and *NME5* in astrocytes of *APOE4* carriers compared to noncarriers (*SI Appendix*, Fig. S22*H*). Interestingly, miBrains with *APOE4* astrocytes compared to all-*APOE3* miBrains displayed a clear increase in lysosomal particle area colocalizing with astrocytes and overall (*SI Appendix*, Fig. S22 *I* and *J* *i*–*iii*). These data suggest that *APOE4* astrocytes both harbor lysosomal dysfunction and promote it in other cells, with this dysfunction correlating with the concomitant increase in reactive species observed in miBrains with *APOE4* astrocytes (Fig. 4 *F*–*I*).

### miBrain Reveals *APOE4* Astrocyte Crosstalk with Microglia Promotes Neuronal Tau Phosphorylation.

*APOE4* has been shown to accelerate amyloid aggregation (79) and tau-mediated neurodegeneration (80), and evidence suggests that removal of astrocytic APOE4 is protective against tau pathology (81). Endogenously, *APOE4* miBrains display increased amyloid aggregation (*SI Appendix*, Fig. S24 *A* and *B*) and tau phosphorylation compared to *APOE3* (*SI Appendix*, Fig. S24 *C*–*F*) (72). Notably, this includes the development of 4R tau pathology and at increased levels in *APOE4* miBrains compared to *APOE3* (*SI Appendix*, Fig. S24 *G* and *H*). To test whether *APOE4* astrocytes could directly promote tau phosphorylation, we compared *APOE3* miBrains with *APOE4* astrocytes to miBrains composed of cells from all *APOE3* or *APOE4* backgrounds. Here, to accelerate pathological progression, we administered a low 20 nM dose of exogenous amyloid beta peptide 1-42 (Aβ-42) resuspended at neutral pH. After 72 h of treatment, increased tau phosphorylation was observed in *APOE4* miBrains via immunoreactivity to phosphorylated tau AT8, especially somatically (Fig. 4*J*). Interestingly, *APOE3* miBrains with *APOE4* astrocytes also displayed substantial tau phosphorylation and increased somatic AT8 intensity compared to *APOE3* miBrains (Fig. 4 *J* and *K*).

As canonical reactive A1 astrocytes are induced through microglial cytokines (82) and microglia-astrocyte crosstalk is thought to mediate immune response to many neurological conditions (83), we asked whether microglia signaling modulates the *APOE4* astrocyte-associated tau phosphorylation we observed. We cultured miBrains with and without iMG. Strikingly, in the absence of microglia, tau phosphorylation was substantially diminished in *APOE4* miBrains (Fig. 4 *L* and *M*). While some aggregates displayed strong immunoreactivity to phosphorylated tau, interneuronal tau colocalizing with tubulin marker TUBB3 was dramatically reduced (Fig. 4 *L* and *M*). This suggests that microglia or microglia-derived cues are necessary to promote the observed tau phosphorylation in the context of *APOE4*.

To assess whether secreted factors resultant from microglia-astrocyte crosstalk contribute to the increased tau phosphorylation, we collected conditioned medium (CM) from *APOE4* microglia-astrocyte coculture, treated with exogenous Aβ-42, and applied it to *APOE4* miBrains (Fig. 4*N*). In parallel, we collected and pooled CM from monocultured *APOE4* astrocytes and from monocultured *APOE4* iMG, each similarly treated with Aβ-42 (Fig. 4*N*). Interestingly, the cocultured microglia-astrocyte CM increased tau phosphorylation when applied to miBrains, while the combined monoculture CM did not (Fig. 4 *O* and *P*). While the extent of tau phosphorylation observed with CM is less than that of *APOE4* miBrains with iMG (Fig. 4 *L* and *M*), which could be due to the dose of soluble cues applied or suggest that other iMG-dependent processes contribute to the observed tau phosphorylation, these data provide evidence that secreted factors from microglia-astrocyte signaling are sufficient to increase tau phosphorylation. This further suggests that crosstalk between astrocytes and microglia produces factors distinct from their monocultured counterparts that promote neurotoxic phenotypes. These results are potentially in line with those of a recent study that reported that conditioned media from *APOE4* iMG with high lipid droplet burden, indicative of a pathological state, and not *APOE4* iMG with low levels of lipid droplets nor *APOE3* iMG, promoted tau phosphorylation in primary neuron culture (84). In fact, examining lipid droplets in miBrains with *APOE3* versus *APOE4* astrocytes, we found increased lipid droplets in the *APOE4* condition, even in otherwise *APOE4* miBrain cell types (*SI Appendix*, Fig. S22 *K* and *L*). This evidences that *APOE4* astrocytes promote a pathological state in iMG that precipitates neurotoxic phenotypes.

## Discussion

We here describe a multicellular coculture inclusive of all six major CNS cell types in an integral tissue mimic, to provide a model in which the contributions from each of these cell types can be queried. We engineered culture parameters to reconstruct important cell phenotypes, structural organization, and functional features of human brain tissue. We have established this miBrain platform mimicking the brain niche inclusive of a biomimetic BBB, neuronal networks, myelin-associated phenotypes, and immune cell components, with brain-mimetic architecture, hallmarks of brain physiology and pathology, and transcriptomic signatures. This system could be uniquely valuable for probing neuroinflammatory processes, neuro-immune and neuro-glial and neurovascular interactions, and cerebrovascular mechanisms. The Neuromatrix Hydrogel could be useful for other brain-modeling applications and the biomaterial design strategy underlying it could also potentially inform the construction of vascularized tissues for other tissue types. While brain organoids have advanced with the fusion of vascular organoids (16, 17) or the introduction of microglia (14, 15), as well as the differentiation of various neuronal subtypes with enhanced maturity and progenitor clusters (1–3, 13), we here provide a modular approach with targeted cell type composition of decoupled cell genotypes and 3D tissue architecture.

While this platform could provide substantial advantages in mechanistic understanding and disease modeling, it also has several limitations. We here use static culture and assess barrier function with junctional proteins as proxies, though future work can be aimed at introducing flow through the vessels within the miBrain via microfluidic approaches that could further enhance the platform biomimicry. Examining BBB properties in *APOE3* versus *APOE4* miBrains is also a valuable future direction. Further, we have not performed immune-gold EM to characterize paired helical filaments. Future work profiling neurons may be better performed using scRNAseq approaches that could isolate and capture neurons with the gentle droplet barcoding method. The viral transduction method used for neurons could also be less cell-type-specific than the nonviral methods, which we were able to harness for isolating other miBrain cell types, in that other cell types like iMG could potentially phagocytose labeled cell debris, contaminating the virally labeled population. While we here compared transcriptomic data to that of monocultured cell counterparts given the greater standardization and broad usage of monocultures, future work can compare miBrain signatures to the various brain organoid protocols and other culture systems. Of note, while we here incorporated cells from the best iPSC differentiation protocols available, future iterations of the miBrain can integrate any advancements in the iPSC field, including in additional cell subtypes. Additionally, future work could be undertaken to study culture conditions longitudinally through later timepoints and to profile changes in cell type ratios and signatures with greater sensitivity.

Harnessing this platform we have found that the miBrain niche can enhance iPSC-derived astrocyte signatures. We found that *APOE4* astrocytes could have an important role in mediating tau pathological progression, thus revealing pathological phenotypes conferred by non–cell autonomous interactions. This work has utilized the unique utility of our miBrain platform to recapitulate multicellular interactions in the CNS and assess cell-type-specific phenotypes with decoupled genotypes. Advancing brain-mimicry in vitro, the miBrain platform could be a vehicle for accelerated mechanistic inquiry and therapeutic development for AD, including extending the model to familial AD patient lines, as well as other neurological indications. Scaling up the platform and miniaturizing the miBrain tissues, we have been able to robustly assess drug responses reproducibly in 48- and 96-well plate formats, potentially positioning the technology for translation. This multicellular mimic inclusive of all six major brain cell types could provide possible advantages in dissecting disease mechanisms, modeling neuropathologies, and evaluating neurotherapeutics across indications.

## Materials and Methods

Detailed information for all experimental methods and materials is provided in *SI Appendix*.

In brief, a total of 7 cell lines were used in this study. All iPSC were maintained in mTeSR1 medium (Stem Cell Technologies) in feeder-free conditions. iPSCs were differentiated into BMECs, pericytes, astrocytes, neurons, oligodendroglia, and iMG. To establish miBrains, cells were dissociated with accutase, counted using an automated cell counter (Countess), encapsulated in a hydrogel precursor solution, and allowed to gelate at 37 °C before being fed with cell culture medium. Please see *SI Appendix* for details.

## Supplementary Material

Appendix 01 (PDF)

Movie S1. Visualization of neurovascular units and microglial integration throughout the miBrain with 3D reconstructions (cyan: neurons via tubulin label, red: BMECs, green: iMG via membrane label).

Movie S2. Visualization of astrocytes integrated with neurons and with the blood-brain barrier throughout the miBrain with 3D reconstructions (green: mCherry-astrocytes, cyan: neurons via tubulin label, blue: Hoechst, red: ZO1-BMECs).

Movie S3. Visualization of oligodendroglia integrated with neurons and Fluoromyelin throughout the miBrain neurovascular units and microglial integration with 3D reconstructions (green: mCherry-oligodendroglia, red: FluoroMyelin, cyan: neurons via tubulin label).

## Data Availability

RNA sequencing data have been deposited in GEO with accession number GSE308670 (85).
