# Supplementary material for: Engineered 3D immuno-glial-neurovascular human miBrain model
Source: Proc Natl Acad Sci U S A. 2025 Oct 17;122(42):e2511596122. doi: 10.1073/pnas.2511596122 (PMC12557797; doi:10.1073/pnas.2511596122)
Supplement: Supplementary file 1 — Appendix 01 (PDF) [file pnas.2511596122.sapp.pdf]

PNAS

Supporting Information for
Engineered 3D Immuno-Glial-Neurovascular Human miBrain Model

Alice E. Stanton[1*†], Adele Bubnys[2,3†], Emre Agbas[2,3], Benjamin James[4], Dong Shin Park[2,3], Alan Jiang[2,3], Rebecca L. Pinals[2,3], Liwang Liu[2,3], Nhat Truong[2,3], Anjanet Loon[2,3], Colin Staab[2,3], Oyku Cerit[2,3], Hsin-Lan Wen[2,3], David Mankus[1], Margaret E. Bisher[1], Abigail K. R. Lytton-Jean[1], Manolis Kellis[3,5,7], Joel W. Blanchard[2,3,10*], Robert Langer[1,6,7,8,9*], Li-Huei Tsai[2,3,5*].

[1]Koch Institute, Massachusetts Institute of Technology; Cambridge, USA.
[2]Picower Institute for Learning and Memory, Massachusetts Institute of Technology; Cambridge, USA.
[3]Department of Brain and Cognitive Sciences, Massachusetts Institute of Technology; Cambridge, USA.
[4]Department of Electrical Engineering and Computer Science, Massachusetts Institute of Technology; Cambridge, USA.
[5]Broad Institute of Harvard and Massachusetts Institute of Technology; Cambridge, USA.
[6]Department of Chemical Engineering, Massachusetts Institute of Technology; Cambridge, USA.
[7]Department of Anesthesiology, Boston Children's Hospital; Cambridge, USA.
[8]Division of Health Science and Technology, Massachusetts Institute of Technology; Cambridge, USA.
[9]Institute for Medical Engineering and Science, Massachusetts Institute of Technology; Cambridge, USA.
[10]Current address: Department of Neuroscience, Black Family Stem Cell Institute, Ronald M. Loeb Center for Alzheimer's Disease, Icahn School of Medicine at Mt. Sinai, New York, NY 10029

*Co-corresponding authors. Li-Huei Tsai, Robert Langer, Joel W. Blanchard, Alice E. Stanton.
Email: lhtsai@mit.edu, rlanger@mit.edu, joel.blanchard@mssm.edu, aestanton@mgh.harvard.edu.

†These authors contributed equally to this work.

This PDF file includes:

Supplementary Methods
Figs. S1 to S24
Legends for Movies S1 to S3
SI References

Other Supplementary Materials for this manuscript include the following:

Movies S1 to S3

Supplementary Methods

Cell lines. iPSC lines were previously generated by the Picower Institute for Learning and Memory iPSC Facility. iPSCs were passaged at 60-80% confluence using 0.5 mM EDTA for 5 minutes and reseeding 1:6 onto Matrigel-coated plates. Previously reported and characterized *APOE3/3*-parental iPSC line and its CRISPR-dCas9-edited isogenic *APOE4/4* line were used in this study[1], along with corresponding NGN2-iPSCs[2]. The *APOE3/3* cell line was derived from a cognitively normal individual and edited to *APOE4/4* homozygous, while an *APOE4/4* line from an AD patient was corrected to *APOE3/3.* Results for *APOE4* astrocytes were corroborated with *APOE4/4*-parental and CRISPR-dCas9-edited isogenic *APOE3/3* line[2]. A ZO1-eGFP line (Allen Institute) was used for tracking experiments, as was a constitutive mCherry-expressing iPSC cell line, which was also used for RNAseq comparisons. For calcium imaging experiments comparing miBrain- and monocultured-neurons, tdTomato and GCaMP3 (addgene plasmid #62814) were introduced via the piggyBac transposon system into NGN2-iPSCs and successfully edited cells were purified based on tdTomato expression using flow cytometry to generate a stable line.

Cell culture. BMECs were differentiated following a protocol similar to previously published protocols[3], [4], with modifications. In brief, on day 0, iPSCs were dissociated to a single-cell suspension and seeded onto basement membrane-grade Matrigel-coated plates at a density of 10,000 cells/cm^2 in mTeSR1 supplemented with 10 μM ROCK inhibitor Y-27632. On day 1, medium was replaced with priming medium (DMEM/F12 media with GlutaMAX, 1% v/v MEM non-essential amino acids, and 1% v/v penicillin:streptomycin) supplemented with 6 μM CHIR-99021 and 10 ng/mL BMP4, feeding daily. On day 4, medium was replaced with endothelial induction medium (hECSR media, 2% v/v B27 supplement, 1% v/v MEM non-essential amino acids, and 1% v/v penicillin:streptomycin) supplemented with 50 ng/mL VEGF-A and 2 μM forskolin, feeding daily. On day 6, cells were passaged onto collagen-coated plates (50 μg/mL collagen type IV diluted in DPBS, coated for a minimum of 30 min at room temperature, then washed once with DPBS prior to use) at a split ratio of 1:3 in endothelial induction medium supplemented with 50 ng/mL VEGF-A and 2 μM forskolin, fed daily. On day 8, cells were passaged by incubating with StemPro Accutase for only ~3-4 min to selectively dissociate endothelial cells, and replated onto collagen-coated plates at a split ratio of 1:5 in endothelial maintenance medium (hECSR media, 2% v/v B27 supplement, 1% v/v MEM non-essential amino acids, 1% v/v penicillin:streptomycin, 10 ng/mL VEGF-A). Cells were fed daily with endothelial maintenance medium and passaged as needed prior to reaching approximately 80% confluency to maintain endothelial cell character. After day 10, expression of endothelial cell-specific marker PECAM was assessed using flow cytometry prior to incorporation into the miBrain (**Fig. S2Ai**). Positive staining for canonical markers PECAM, vWF, CLDN-5, VE-CAD, and ZO-1 was confirmed via immunohistochemistry (**Fig. S1A**). Absence of pericyte marker PDGFRb and epithelial cell marker EPCAM, in addition to presence of microvascular marker CD34, were further confirmed by flow cytometry.

iPSCs were differentiated into pericytes based on a previously described protocol[3]. In brief, iPSCs were maintained under feeder-free conditions until colonies reached approximately 60% confluence. On day 0, cells were dissociated using accutase and seeded on hES-grade Matrigel coated plates at a density of 3,000 cells/ cm^2 and fed with mTeSR media supplemented with 10 μM ROCK inhibitor Y-27632. On day 1, media was replaced with priming media (DMEM/F12 media, 8 μM CHIR-99021, 25 ng/mL BMP4). On day 4, media was replaced with pericyte induction media (DMEM/F12 media, 100 ng/mL PDGF-B, 100 ng/mL activin A). On day 5, cells were passaged using accutase and replated on Matrigel coated plates at a density of 1e^5 cells/ well of a 6-well plate and fed with pericyte induction media supplemented with 10 μM ROCK inhibitor. Pericytes were maintained without passage for an additional 14 days, feeding with pericyte expansion media (DMEM/F12 media, 10 ng/ml PDGF-B) every two days. Positive staining for canonical markers NG2 and PDGFRβ was confirmed via immunohistochemistry (**Fig. S2C**). Expression of pericyte-specific marker PDGFRβ was assessed using flow cytometry prior to incorporation into miBrains (**Fig. S2Aii**).

Astrocytes were generated according to a previously published protocol[5], with modifications. Positive expression of astrocyte marker S100b and negative expression of neuronal progenitor marker SOX10 were validated using immunohistochemistry prior to incorporation into miBrain co-cultures (**Fig. S2D**).

Expression of CD44 was assessed using flow cytometry (**Fig. S2Aiii**). OPCs were generated according to the protocol described in[6]. Positive expression of OPC markers PDGFRα and O4 were validated using FACS (**Fig. S2Aiv**) and immunohistochemistry (**Fig. S2E**) prior to incorporation into miBrain co-cultures. iMG were generated according to a previously published protocol[7]. Positive expression of microglia marker CD45 was validated using FACS prior to incorporation into miBrain co-cultures (**Fig. S2Av**). Canonical marker detection via immunohistochemistry for Iba1, P2RY12, and TMEM119 was confirmed (**Fig. S2F**). Neurons were differentiated according to a previously published protocol[8]. In brief, iPSCs harboring tetON-NGN2 (NGN2-iPSC) were seeded on Matrigel-coated plates and treated with doxycycline (5μg/ml) for 5 days to induce NGN2 overexpression. Cells were treated with 5μM blasticidin for 3 days to eliminate populations lacking tetON-NGN2, then treated with 5μM puromycin for 2 days starting at day 3 to select for NGN2-positive cells. Neurons were dissociated and incorporated into miBrains on day 7 of this protocol. Expression of PSA NCAM was confirmed via flow cytometry (**Fig. S2Avi**) and positive marker expression of TUBB, MAP2, and NeuN was confirmed via immunohistochemistry (**Fig. S2G**).

Neuromatrix hydrogel encapsulation and miBrain formation. Validated iPSC-differentiated cells were co-cultured in miBrains at the following ratios: 5 M/mL BMECs, 0.2 M/mL pericytes, 1.5 M/mL astrocytes, 0.75 M/mL OPCs, 5 M/mL neurons, and 0.6 M/mL iMG, unless stated otherwise. miBrains were cultured in miBrain cell culture medium consisting of Human Endothelial Serum-Free Medium (ThermoFisher Scientific) with 1% pen/strep (Sigma-Aldrich), 1% NEAA (ThermoFisher Scientific), 1% CD lipids (ThermoFisher Scientific), 1% Astrocyte Growth Supplement (ScienCell), 2% B-27 (ThermoFisher Scientific), 10 µL/ mL Insulin (ThermoFisher Scientific), 1 µM db-cAMP (Sigma Aldrich), 50 µg/ mL Ascorbic Acid (Sigma Aldrich), 10 ng/ mL NT-3 (PeproTech), 10 ng/ mL IGF-I (PeproTech), 100 ng/ mL Biotin (Sigma Aldrich), 60 ng/mL T3 (Sigma Aldrich), 4 µg/ mL Folic Acid (Sigma Aldrich) supplemented, 1 nM SAG (Tocris), and 50 ng/ mL VEGF-A (PeproTech). Upon the incorporation of iMG, 25 ng/mL M-CSF (Sigma Aldrich) was added and maintained in the media and after week 2, VEGF-A concentration was reduced to 10 ng/mL.

The optimized Neuromatrix Hydrogel formulation is a dextran-based hydrogel[9], iteratively tuned for the co-culture of our six iPSC-derived brain cell types, and with the addition of brain extracellular matrix proteins. To simplify the formulation for maximal adoption and usability for biology labs, we identified commercial vendors for each of the components. A pre-reaction mix of dextran polymer for a final 3 mmol/L thiol-reactive groups (TRU-SGD, Sigma or TRUE7 Kit, Sigma, comprising polymer, buffer, and crosslinker components) was brought to neutral pH with concentrated buffer (TRU-B72, Sigma) and reacted with 3.0 mM CRGDS basement membrane-mimicking peptide (GenScript) and 50 µg/mL human recombinant versican (R&D Systems), unless otherwise stated, for 20 minutes at room temperature. Cells at the above concentrations were combined into a single pellet, resuspended in miBrain cell culture medium described above to complete the balance of the volume, and mixed well with the pre-reaction mix. Cell-degradable crosslinker (TRU-CD, Sigma) containing MMP-cleavable peptide sequence (Pro-Leu-Gly-Leu-Trp-Ala) for a final concentration of 3 mmol/L thiol groups was mixed well with the resuspended cells and pre-reaction mix and quickly dispensed into dishes and allowed to polymerize at 37°C for 25 minutes before being submerged in miBrain cell culture medium. The medium was exchanged after one hour to remove any reaction byproducts. miBrains were maintained in culture by feeding every other day. Experiments were performed between weeks 1 and 4 unless otherwise stated.

Immunohistochemistry and confocal imaging. For immunohistochemistry on cell monocultures, cells were passaged onto Matrigel-coated chamber slides (MatTek) and fixed for 20 minutes in 4% PFA, washed with PBS, permeabilized in PBST (PBS, 0.1% Tween-20) for 10 minutes, incubated in blocking solution (PBS, 3% BSA, 2% donkey serum) for 30 minutes, incubated with primary antibody (1:500 unless otherwise specified) for 1 hour at room temperature or overnight at 4°C, washed five times with PBS and incubated with secondary antibody (1:500) and Hoechst 33342 (1:1000) in blocking solution for one hour at room temperature, then washed another five times.

For Claudin-5 BMEC immunohistochemistry, this protocol was followed, with the exception that cells were instead fixed in methanol by incubating for 5 minutes at -20°C, washing three times for 10 minutes with PBS, and then directly blocked in 2% serum and 3% BSA in PBS for 1 hour.

For miBrain immunohistochemistry, miBrains were fixed for 20 minutes in 4% PFA, washed with PBS, permeabilized in PBST (PBS, 0.1% Tween-20) for 30 minutes, incubated in blocking solution (PBS, 3% BSA, 2% donkey serum) for 2 hours, incubated with primary antibody (1:500 unless otherwise specified) overnight at 4°C, washed five times with PBS for a minimum of 15 minutes total and incubated with secondary antibody (1:500) and Hoechst 33342 (1:1000) in blocking solution for one hour at room temperature, and then washed another five times for a minimum of 15 minutes total.

High magnification imaging was performed using Zeiss LSM 710 and LSM 900 confocal microscopes.

Reduced Culture Condition Comparisons. Comparative cultures in Matrigel were encapsulated in 100% hESC-Qualified, LDEV-free Matrigel (Corning) and allowed to gelate for 45 minutes at 37°C. Conditions incorporating extracellular matrix proteins into Matrigel-encapsulated 3D co-cultures were incorporated at 50 µg/mL protein.

Simplified co-cultures of neurons and astrocytes were co-cultured at 5 M/mL and 2.5 M/mL, respectively, in neuronal medium.

For optimizing cell ratios, miBrains were constructed with one given cell type of altered density, as specified (BMECs: 1 or 8 M/mL vs. 5 M/mL, pericytes: 0.02 or 2 M/mL vs. 0.25 M/mL, astrocytes: 0.75 or 3 M/mL vs. 1.5 M/mL, OPCs: 0.15 or 2.25 M/mL vs. 0.75 M/mL, neurons: 1 or 8 M/mL vs. 5 M/mL, and iMG: 0.2 or 1.8 M/mL vs. 0.6 M/mL), and otherwise preserving the concentrations for the other cell types. For miBrains with modified ratios of pericytes, astrocytes, OPCs, and iMG, the respective cell type of interest was pre-labeled prior to incorporation using a cell membrane dye (Vybrant Cell-Labeling Solutions, Life Technologies) following the manufactuer's instructions. For all miBrains, a constitutive mCherry-expressing iPSC cell line was used for BMECs and at day 6, cultures were pre-incubated with a tubulin live dye (siR-Tubulin, Cytoskeleton) overnight prior to imaging. To quantitate resultant changes, in ImageJ, the mean intensity per image was measured for tubulin and for vessels a plug in was automatically applied to threshold images with mCherry-BMEC signal, select positive signal, and measure signal area.

For elucidating the effects of hydrogel components, miBrains were constructed with or without RGD at 3.0 mM and with or without VCN at 50 µg/mL. A constitutive mCherry-expressing iPSC cell line was used for BMECs. miBrains were fixed at day 7 and immunohistochemistry performed for neurofilament and GFAP. To quantitate resultant changes, in ImageJ, the mean intensity per image was measured for neurofilament and GFAP and for vessels a plug in was automatically applied to threshold images with mCherry-BMEC signal, select positive signal, and measure signal area.

Rheological Analysis. A Discovery HR-2 Rheometer (TA Instruments) was used to measure the storage modulus (G') of dextran-based hydrogels. A plate-plate flat configuration was used (20 mm diameter) with a sand-blasted plate to prevent slipping. The top plate of the rheometer was slowly lowered until touching the top of the sample and measuring a maximum axial force of 0.01 N. To avoid dehydration, PBS was applied to the outer rim of the sample. A wait time of 180 s was set for the beginning of the test to allow the specimen to conform to the temperature controlled by the rheometer plates, maintained at 37 °C. For frequency sweep analysis, a shear strain of 1% was applied on the upper plate, varying the frequency from 0.01 to 25 Hz. For strain sweep analysis, an angular frequency of 0.1 Hz was applied on the upper plate, varying the strain from 0.1 to 1000%.

Whole-miBrain imaging. To image entire miBrains to visualize cell distribution and track miBrains over time, 9-by-9 tile scans were acquired over 264 µm z-height with a 20X objective in a Cytation 10 confocal

imager system (Agilent BioTek). Images were stitched via the imaging processing tools in the imager software and z-projections generated via maximum intensity (BioTek).

Monoculture quantification. Non-junctional markers were quantified in Imaris following background subtraction of signal in iPSC controls stained in parallel for each marker of interest. Surfaces for Hoechst and the marker of interest were created for each image and the total number of overlapping cells was quantified.

BMEC junctional markers were quantified in ImageJ using the Trainable Weka Segmentation function to identify marker expression localized to cell borders. Briefly, WEKA classifiers were trained and applied on maximum intensity z-projections of confocal images for each marker across the entire image set. Reference channels (phalloidin or PECAM) were used to define cell borders via the Analyze Particles function (ROI size 5 µm – infinity to minimize noise). Identified border regions were overlaid on the junctional marker image and area of overlap was measured across all identified regions. Thresholds were set at 20% (CLDN-5 and VE-cad) and 2% (ZO-1) to determine if cells were positive for the junctional marker of interest based on manual confirmation on a smaller image set. This workflow is automated and unbiased in selecting border expression. However, the protocol is limited by the border-to-cytoplasmic signal ratio caused by background that can lead to an underrepresentation of the border signal and <100% cell confluency in samples that likely decreases junctional protein expression.

TEER Assay. To assess trans-endothelial electrical resistance (TEER) across BMEC monolayers, 12 mm diameter Transwells with 0.4 µm Pore Polyester Membrane Inserts (CellQART) were coated with 400 µg/mL collagen I and 100 µg/mL fibronectin and seeded at 0.5 M cells/cm². When the cells reached 100% confluence, STX2 TEER Chopstick Electrodes (World Precision Instruments) were inserted with one electrode in the inner chamber of the Transwell and one electrode in the outer chamber of the Transwell and the measurements from a Millicell ERS2 Voltohmmeter (Millipore) were recorded. TEER was calculated by subtracting the resistance across a blank Transwell (no cells seeded on membrane, immersed in cell culture medium) and then multiplying by the membrane surface area.

Live/Dead assay. To assess cell viability in miBrains, Calcein AM and Ethidium homodimer-1 from the LIVE/DEAD® Viability/Cytotoxicity Kit for mammalian cells were applied following manufacturer instructions (Invitrogen). For a reference control, one set of miBrains was pre-treated in 70% methanol for 30 min. In brief, cells were washed with DPBS, incubated for 30 minutes at room temperature while protecting from light with live/dead reagents and Hoechst nuclear label, and then imaged on a Cytation 10 confocal imager system with 4X objective and epifluorescence imaging mode, maintaining the same experimental protocol and imaging settings for each group (Agilent BioTek). To co-visualize neurons, miBrains were pre-incubated with a tubulin live dye overnight (Cytoskeleton) and also imaged in the far-red channel. Cell viability was quantified based on image intensity with the live fraction determined by the mean intensity of the live signal divided by the sum of live and dead mean intensities, applied across methanol controls and timepoint groups.

3D reconstructions and side views. Imaris software was used to visualize 3D z-stack images. Slice views were used to visualize lumenized vessels, pericyte localization proximal to vessels, and myelination around neuronal projections. Surfaces were computationally constructed from each imaging channel to visualize the distribution of cells throughout whole miBrains, pericyte localization proximal to vessels, iMG interactions with vessels, and myelination around neuronal projections.

Antibodies. Primary antibodies for immunohistochemistry used are as follows unless otherwise specified:

Target	Host	Vendor Information	Dilution
Neurofilament (smi311)	mouse	BioLegend 837802	1:500
IBA1	guinea pig	Synaptic Systems 234 004	1:500

Tubulin (TUBB3, TUJ1)	rabbit, mouse	BioLegend 802001, 801213	1:500
NG2	rabbit	Abcam ab255811	1:500
Aquaporin4 (AQP4)	rabbit	Thermo Fisher PA5-53234	1:500
Myelin Basic Protein (MBP)	chicken	Millipore AB9348	1:500
PECAM	sheep	R&D Systems AF806	1:500
VE-Cadherin (VE-CAD)	goat	R&D Systems AF938	1:100
ZO-1	mouse	Thermo Fisher MA3-39100-A488	1:100
Claudin 5 (CLDN-5)	mouse	Invitrogen 4C3C2 352588	1:250
Von Willebrand Factor	rabbit	Abcam ab154193	1:100
GFAP	goat	Abcam ab53554	1:500
Connexin 43 (Cx43)	rabbit	Abcam ab11370	1:500
S100β	rabbit	Abcam ab41548	1:500
P2RY12	rat	BioLegend 848002	1:500
MAP2	chicken	BioLegend 822501	1:500
vGlut1	chicken	Synaptic Systems 135316	1:500
PSD95	rabbit	Abcam ab18258	1:500
Phospho-Tau (AT8)	mouse	Thermo Fisher MN1020	1:500
PDGFRβ	mouse	Abcam ab69506	1:500
SOX2	goat	R&D Systems AF2018	1:500
O4	mouse	Millipore MAB345	1:500
Synapsin	guinea pig	Synaptic Systems 106104	1:500
Oct4	rabbit	Cell Signaling Technology 2890	1:500
Amyloid 82E1	mouse	Immuno-Biological Laboratories 10323	1:500

Corresponding Alexa secondary antibodies (ThermoFisher) were used at 1:500. Alexa Fluor 647 phalloidin (1:400) was incubated along with secondary antibodies.

Flow cytometry. Cells were dissociated using accutase and washed with FACS buffer (DPBS, 0.5% BSA, 2 mM EDTA), filtered through a 40 µm cell strainer, then resuspended in blocking buffer (DPBS, 1% FBS) with 1:200 antibody and incubated for 25 minutes on ice protected from light. Cells were then washed with FACS buffer and resuspended in buffer with DAPI before filtering through a 40 µm cell strainer and proceeding with FACS. Flow cytometry was performed on a BD LSRFortessa cell analyzer equipped with 355 nm, 405 nm, 488 nm, 561 nm, and 640 nm lasers. Positive expression of cell type specific markers was assessed relative to isotype controls.

Antibodies used for flow cytometry were as follows:

**Target**	**Host / Isotype**	**Vendor Information**	**Dilution**
CD31-FITC	Mouse IgG1	BioLegend 303104	1:200
CD140b-PE	Mouse IgG2a	BD 558821	1:200
CD44-APC	Mouse IgG1	BioLegend 338806	1:200
PDGFRa-APC	Moue IgG1	R&D systems FAB1264A	1:200
PSA NCAM-APC	Mouse IgM	Miltenyi Biotec 130-120-437	1:200
CD45-APC/Cy7	Mouse IgG1	BD 561863	1:200
FITC mouse IgG1, k isotype control	Mouse IgG1	BioLegend 400107	1:200
PE mouse IgG2a, k isotype control	Mouse IgG2a	BD 555574	1:200
APC mouse IgG1, k isotyle control	Mouse IgG1	BioLegend 400119	1:200

APC mouse IgM isotype control	Mouse IgM	Miltenyi Biotec 130-122-931	1:200
APC/Cy7 mouse IgG1, k isotype control	Mouse IgG1	BD 557873	1:200
P2RY12-PE	Mouse IgG2a	BioLegend 392103	1:200
PE mouse IgG2a, k isotype control	Mouse IgG2a	BioLegend 400213	1:200

Isolation and RNAseq of cell types from miBrain. To isolate cells from miBrains for RNAseq, miBrains were matured for 2 weeks prior to cell isolation and RNA sequencing. Corresponding cell monocultures were maintained in parallel for 2 weeks and then sorted using the same parameters as the miBrains. To minimize batch-dependent effects, the same batch of differentiated cells was used for the monoculture condition and the miBrain condition in each RNAseq experiment.

Neurons were isolated by first virally labeling neurons from the same batch with pAAV-CAG-tdTomato for 24 hours (1:50 dilution, Addgene 59462-AAV1) prior to incorporation into miBrain or monoculture, a subset of which were incorporated into miBrains and a subset of which were maintained in monoculture. To ensure sufficient capture of target cell populations, 15 individual miBrains were pooled per replicate, and three replicates were isolated per experiment. After 2 weeks, tdTomato-neuron miBrains and monoculture samples were harvested. Astrocytes and oligodendroglia were isolated using a similar approach, but by utilizing endogenously mCherry-expressing astrocytes. iMG were incorporated into 2-week old miBrains, while a subset from the same batch were maintained in monoculture, and after an additional four days, sorted based on CD45-Cy7.

To dissociate miBrains, samples were washed with PBS, then were incubated for 5-10 minutes in a mixture of accutase, collagenase IV (1:20), and dextranase (1:20). These were triturated 15-20 times with a P1000 pipette to form a homogeneous suspension, then pelleted and washed with DPBS twice. Astrocytes, iMG, and neuron monocultures grown in parallel were dissociated using accutase, then washed with DPBS prior to proceeding with antibody labeling and sorting. Oligodendroglia were fixed with 4% PFA on ice for 20 minutes prior to dissociation to ensure stability. iMG were incubated with CD45-Cy7 alongside isotype controls at 1:200 on ice for 25 minutes before proceeding with sorting.

Dissociated miBrains and monocultures were resuspended in FACS buffer (DPBS, BSA, EDTA) with DAPI and filtered through a 40 µm strainer. Samples were sorted on a BD FACSAria equipped with a 70 µm nozzle. Neurons were FACS-isolated based on tdTomato expression, astrocytes and oligodendroglia based on mCherry, and iMG based on CD45 expression. RNA was harvested in triplicate and in parallel from 20K neurons, 50K astrocytes, 50K oligodendroglia, or 2K CD45-positive iMG per replicate. Cells were gated on FSC-A vs SSC-A to separate intact cells from debris, then FSC-H vs FSC-W and SSC-H vs SSC-W to separate single cells from doublets, then DAPI to exclude dead cells, and then the channel corresponding to the antibody used. Cells were sorted into 96-well plates containing 50 µl RNAprotect per well.

For live samples, RNA was isolated from sorted cells using the Qiagen RNeasy isolation kit according to the manufacturer's protocol. For fixed samples, sorted cells were spun down and resuspended in 200 µL digestion buffer (RecoverAll kit) supplemented with 1:50 Proteinase K, incubated at 50°C for 15 minutes, and then at 80°C for 15 minutes with no agitation to de-crosslink RNA from fixed tissue. 800 µL Trizol was added to the sample and incubated for 5 minutes, then 215 µl chloroform was added and the sample was vortexed vigorously for 30 seconds. Sample was transferred to a 5-prime phase lock heavy gel tube and centrifuged for 15 minutes at 12,000 g. The upper layer containing RNA was transferred to a fresh 1.5 ml microcentrifuge tube and an equal volume of 100% ethanol before proceeding with RNA isolation using Zymo Direct-zol RNA microprep kit according to the manufacturer's protocols. Standard RNA quality control was performed using AATI fragment analyzer. RNA sequencing libraries were prepared using NEB Ultra II PolyA stranded library and sequenced using Illumina NextSeq500.

RNA-sequencing data analysis. RNA-sequencing analysis was performed for each batch in a similar fashion. For preliminary QC, FastQC was used to look at quality. For each sample, gene reads were mapped to GRCh38.p13 and genes to GENCODE v43 using STAR 2.7.9a. These were then counted as reads per-sample using featureCounts from Subread version 1.6.2. Each sample was loaded into R (version 4.2.2) using a BioConductor (version 3.16) based workflow to perform QC and further analysis. Reads were normalized to trimmed mean of M-values (TMM) normalization using the R command "cpm(calcNormFactors(rawCounts, "TMM"))" in edgeR. Differential expression analysis was also performed using edgeR, keeping genes with a threshold of 100 counts per million (CPM) in at least 25% of the samples used in differential expression analysis. The DEGs were calculated in edgeR using a quasi-likelihood F-test with no covariates. Comparisons using a likelihood ratio test (LRT) in edgeR and DESeq2 were performed; but similar results were obtained. Gene ontology analysis was performed on significantly upregulated and downregulated DEGs using Panther DB, which identified GO terms with an FDR p-value less than 0.05. From these GO terms were selected the most biologically salient pathways, presented in order of fold-enrichment. iPSC data was downloaded and reprocessed using the same above RNA-sequencing workflow from GEO GSE102956. Single-cell RNAseq data for comparisons was downloaded from the Mathys, et al., 2023[10] aging brain atlas from the linked website https://compbio.mit.edu/ad_aging_brain/ , filtered for non-pathological samples, and summarized to major cell type per-donor by summing gene counts per sample. Microglia states were similarly processed from https://compbio.mit.edu/microglia_states/[11]. Comparisons between bulk RNAseq were performed by taking common genes between the two references above 100 counts per million in 25% of each of the groups across each RNAseq sample group in order to make the comparisons the same across case and control. Code utilized to analyze the data is included on GitHub at https://github.com/kellislab/miBrain. PCA gene loadings of BMECs to prior literature were performed by running PCA on the Z-scored TMM expression of samples and taking the top 100 gene loadings of the first principal component and the bottom 100 gene loadings of the first principal component.

Calcium imaging of neurons. To generate neurons with GCaMP expression, iPSCs containing tetON Ngn2 were transfected with a plasmid driving GCaMP3 expression under the CAG promoter (Addgene #62810), then differentiated into neurons and incorporated into miBrain cultures as described above. In order to facilitate live imaging, miBrains were cultured on 3-cm glass bottom dishes (MatTek). Two-photon microscopy was performed on an Olympus FVMPE-RS microscope equipped with SpectraPhysics InsightX3 DeepSee laser tuned to 920nm. Images were acquired using Fluoview acquisition software (Olympus), we simultaneously acquired images in the red channel (bandpass filter 575-645 nm) to visualize tdTomato-expressing neurons, and the green channel (bandpass filter 495-540 nm) for GCaMP signal. Time courses of spontaneous calcium activity were collected at various time points, starting at week 4. Regions of interest were defined as tdTomato-positive neuronal soma and were manually outlined using ImageJ software and then quantified in Matlab. Calcium spikes were defined as periods in which GCaMP signal exceeded two standard deviations above baseline.

Optogenetic evaluation of neuronal connectivity and neurovascular responsiveness. NGN2-neurons were differentiated from NGN2-iPSCs through the 7-day protocol and then transfected with either pAAV-CAG-hChR2(H134R)-mCherry (Addgene) or pAAV-CAG-tdTomato (Addgene). To evaluate the connectivity of miBrain-neurons, a second population of neurons were transfected with pAAV-CAG-GCaMP6f (Addgene). To evaluate vascular response to neuronal activity, BMECs were transfected with pAAV-CAG-GCaMP6f (Addgene). 48 hours later, positive transfection was confirmed, transfected cells were rinsed with PBS three times prior to dissociation, and miBrains were constructed. To evaluate neuronal connectivity, one set of miBrains was constructed with equal numbers of GCaMP6f- and ChR2-neurons, while a second control set was constructed with equal numbers of GCaMP6f- and tdTomato-neurons. To evaluate vascular response to neuronal activity, one set of miBrains was constructed with ChR2-neurons and GCaMP6f-BMECs, while a second set was constructed with tdTomato-neurons and GCaMP6f-BMECs. Calcium transients were recorded at day 10 using a Cytation 10 confocal imaging system (Agilent BioTek) with a 0.08 s sampling rate for neuronal recordings and 0.13 s sampling rate for BMEC recordings, imaging GFP with blue excitation light.

Calcium imaging of pericytes. To generate pericytes with GCaMP expression, iPSCs were transfected with a lentivirus driving GCaMP6 expression under the CAG promoter, then differentiated into pericytes and incorporated into miBrain cultures together with BMECs generated from an iPSC line with constitutive mCherry expression. Calcium imaging experiments were performed on 2-week old miBrains using the Olympus FVMPE-RS 2-photon microscope as described in the previous section. For vascular dynamics assay, spontaneous activity of pericytes was recorded for approximately 5 minutes, then 100 nM ET-1 was manually injected, followed by a recovery time of 5 minutes before imaging calcium activity for 5 minutes. Regions of interest were defined as pericyte cell bodies and manually outlined using ImageJ software. A calcium transient was defined as a period of time in which mean pericyte calcium signal intensity was greater than two standard deviations above the mean of baseline.

Calcium imaging of astrocytes. To characterize calcium activity in iPSC-derived astrocytes, astrocytes at the conclusion of the differentiation protocol were re-plated in an assay plate at 100 K/cm^2 in astrocyte medium and allowed to recover for 2 days. Astrocytes were rinsed with HBSS and 30 minutes later, Fluo-4, AM (ThermoFisher Scientific) was applied at 1:1000 in astrocyte medium and incubated for an additional 30 minutes. Recordings were collected using the Cytation 10 automated imager system (Agilent BioTek) with environmental control.

MEA recording. A MaestroPro MEA system (Axion Biosystems) was utilized to record electrical activity on miBrains. miBrains were seeded directly onto MEA plates (Axion Biosystems) and maintained in culture according to our standard protocol. 30 minute recordings were collected from miBrains and analyzed using Neural Metrix software (Axion Biosystems).

To assess the effect of neuromodulators on miBrain-neuron activity, activity was first recorded on the MEA system with 5 min. recordings. Tetrodotoxin (TTX; Tocris; 8, 80, 800 nM), NBQX (Tocris; 1, 10, 50, 200 µM), (+)-MK 801 maleate (MK801; R&D Systems; 50, 100, 500 µM), MNI-caged-L-Glutamate (R&D Systems; 0.1, 1, 10, 20 mM), and KCl (Sigma-Aldrich; 0.5, 1 mM) were applied and activity was recorded 2 minutes after dosing (n = 4 miBrains/ group). The fold change in mean firing rate from before versus after dosing was calculated for each treatment condition.

Transmission electron microscopy. Samples were fixed with 2.5% glutaraldehyde, 2% paraformaldehyde, in 100 mM sodium cacodylate buffer, pH 7.2, for 2-3 hours at 4 °C and then washed 3 times for 10 min. each with 100 mM sodium cacodylate buffer, pH 7.2. They were post-fixed in 1.25% potassium ferrocyanide with 1% osmium tetroxide in 100 mM sodium cacodylate buffer, pH 7.2, for 1 hour at 4 °C, and rinsed with 100 mM sodium cacodylate buffer 3 times for 10 min. each at room temperature. Then they were rinsed 3x with 50 mM maleate buffer, pH 5.2, for 10 min. each at room temperature and incubated in 2.0% uranyl acetate in 50mM sodium maleate overnight at room temp in the dark. The next day the samples were taken through a series of ethanol dehydrations starting at 30% through to 100% ethanol, then submerged in propylene oxide for 30 min. and propylene oxide : epon resin (1:1) overnight. The following day the samples were incubated in propylene oxide : resin (1:2) all day and then in straight resin overnight. They were placed in BEEM capsules and polymerized in the 60°C oven for 48 hours. Sections were cut on a Leica Ultracut UC7 Ultramicrotome using a Diatome diamond knife at a thickness setting of 60 nm and collected on 200 mesh nitrocellulose/carbon coated grids. The sections were examined using a FEI Tecnai T-12 Spirit at 120 kV and imaged using an AMT XR-16 Bottom mount CCD camera.

Assessment of BBB Modulators on Barrier Function. miBrains were constructed with endogenously mCherry-expressing BMECs. On day 4, thrombin from bovine plasma (Sigma-Aldrich; 100 nM), hydrocortisone (Sigma-Aldrich; 140 nM), lipopolysaccharides (LPS) from *Escherichia coli* O26:B6 (Sigma-Aldrich; 10, 100, and 1000 µg/mL), and recombinant TNF-α (Peptrotech;10, 100, and 1000 ng/mL) were applied. Whole-miBrain images were collected after 24 hours (Cytation 10, Agilent BioTek, 4x objective, z-stack tile-scans). In Fiji, vessels were analyzed via automated analysis, in which the analyze particles function was applied to maximum projection images of whole-miBrains to analyze vessels of 100 square

μm and above. The average vessel area and total vessel area were recorded and plotted in GraphPad Prism. Whole-miBrain areas were quantified via automated analysis from the vascular regions, increasing the threshold to include all area within the vessel networks as positive area. miBrains were fixed at 48 hours, immunolabeled for PECAM and ZO-1, and imaged via confocal microscopy (Cytation 10, Agilent BioTek, 20x objective, z-stack tile-scans). In Fiji, vessel regions were defined using the PECAM channel via automated analysis, ZO-1 signal intensity was assessed within PECAM-positive regions, and results were plotted in GraphPad Prism.

Assessment of Astrocyte Response to LPS treatment. miBrains were matured for 1 week before applying 100 μg/mL LPS for 48 hours versus no-treatment controls (n = 4 miBrains/ group). After 48 hours, miBrains were fixed in 4% PFA and analyzed via immunohistochemistry.

Amyloid treatment. Stock solutions of amyloid beta 1-42 peptide (Anaspec) were resuspended in PBS. Peptides were administered to miBrains at 20 nM for three days to induce pathology.

Real-time monitoring miBrain. Live imaging of miBrains were performed on a Zeiss LSM 900 confocal microscope using a 20x objective with environmental control of humidity, temperature, and CO2. Mitochondria were similarly assessed using BioTrack 633 Red Mito Dye (Sigma-Aldrich) and lysosomes using BioTrack NIR633 LysO Dye (Sigma-Aldrich). Tubulin was similarly visualized live with a small molecule dye (Cytoskeleton). Astrocytes were labeled for live assays prior to incorporation into miBrains using an AAV (pAAV-CAG-tdTomato, Addgene or AAV9-CAG-GFP, Applied Biological Materials). Microglia were labeled for live assays prior to incorporation using a cell membrane dye (Vybrant Cell-Labeling Solutions, Life Technologies). A stably expressing mCherry cell line was utilized for live imaging endothelial cells that was previously developed using pAAVS1-P-CAG-mCh (Addgene). FluoroMyelin dye was used for live labeling myelin in miBrains (Thermo Fisher Scientific).

Reactive Species. Reactive species were assessed by treating miBrains with biosensors for nitric oxide (siRNO Nitric Oxide Live Cell Dye, Sigma-Aldrich), peroxynitrite (BioTrack 515 Green ONOO-, Sigma-Aldrich), or hydrogen peroxide (Green H2O2, Sigma-Aldrich) following the manufacturer instructions. To quantify reactive species, in ImageJ, the mean intensity of reactive species per image was measured. A plug in was used to automatically threshold images on the Hoechst channel, select positive signal, and measure signal area, to normalize images by the nuclear area. Similarly, to assess the positive area, a plug in was used to automatically threshold images on the channel of interest, select positive area, and measure the signal area.

***APOE3* vs *APOE4* Marker quantification.** The mean intensities per marker were assessed in ImageJ. For co-localization analyses, a plug in was used to automatically threshold images on the channel defining the area, select positive area, and then measure the signal in the second channel. For normalizing by nuclei, a plug in was used to automatically threshold images on the Hoechst channel, select positive signal, and measure signal area.

Western blots. Whole tissue lysate protein fractions from miBrains were prepared by washing samples with cold PBS, then adding RIPA buffer with protease inhibitor (150 μL/sample) (Sigma-Aldrich cat: R0278) and incubating on ice for 20 minutes. Tissue was manually disrupted with a p1000 pipette, then centrifuged at 14,000g for 15 minutes at 4°C. Supernatant containing the protein fraction was then saved and quantified using a Pierce 660 assay (Thermo Fisher cat: 22662). For western analysis, 10 μg of total protein in 6X Laemmli SDS sample buffer (Thermo Fisher Scientific) was heated for 5 minutes at 95°C and subjected to SDS-PAGE. Electrophoresis and transfer onto PVDF membranes was performed using the Biorad Mini-Protean Gel Electrophoresis System and Trans-blot Turbo Transfer System, according to manufacturer recommendations. After transfer, membranes were blocked in TBST (Tris-buffered saline, 0.02% Tween-20) with 5% BSA for 1 hour, and incubated overnight with primary antibody at 4°C in a 1:1000 dilution. After subsequent washes with TBST, membrane was incubated in HRP-linked secondary antibody in a 1:500 dilution for 1 hour . Blots were washed again with TBST, developed with SuperSignal

West Pico Chemiluminescent Substrate (Thermo Fisher Scientific), and imaged using the Biorad ChemiDoc MP Imaging System. Protein band densitometry was measured using the FIJI Plot Lanes function as area under the curve, and normalized to the respective internal control (β-Actin) band. Graphs and statistical analysis were performed in GraphPad prism.

Antibodies used for Western blot were as follows:

**Target**	**Host**	**Catalogue Number**
Phospho-Tau (Ser396)	Rabbit	Invitrogen 44-752G
Tau (4R isoform RD4)	Mouse	Sigma Aldrich 05-804
Tau (HT7)	Mouse	Invitrogen MN1000
β-Actin	Mouse	Sigma Aldrich A5316
β-Actin	Rabbit	Sigma Aldrich ZRB1312
HRP-linked anti-mouse IgG	Sheep	Cytiva NXA931
HRP-linked anti-rabbit IgG	Donkey	Cytiva NA934

Recording of action potentials and whole-cell currents. For electrophysiological recordings, miBrains were removed from the incubator and culture media was replaced with an artificial cerebrospinal fluid (ACSF) solution containing 125 mM NaCl, 2.5 mM KCl, 1.2 mM NaH_2PO_4•H_2O, 2.4 mM $CaCl_2$•$2H_2O$, 1.2 mM $MgCl_2$•$6H_2O$, 26 mM $NaHCO_3$ and 11 mM D-Glucose. miBrains were placed in a recording chamber and perfused with oxygenated ACSF at a constant rate of 2 mL/min at ~32°C. Cells were visualized using infrared differential interference contrast imaging on an Olympus BX-50WI microscope.

Electrophysiological recordings were made using an Axon Multiclamp 700B patch-clamp amplifier (Molecular Devices) and Clampex software (version 11.2, Molecular Devices). Signals were filtered at 1 kHz using the amplifier's four-pole, low-pass Bessel filter, digitized at 10 kHz with an Axon Digidata 1550B interface (Molecular Devices) and stored on a computer. Action potentials were generated by injecting various steps of currents in the whole-cell current clamp configuration and episodic stimulation acquisition mode. Whole-cell currents were recorded from a holding potential of −80 mV by stepping to various voltages using the whole-cell voltage clamp configuration and episodic stimulation acquisition mode. Recording electrodes pulled from borosilicate glass pipettes (World Precision Instruments) when filled with the internal solution: 120 mM K gluconate, 5 mM KCl, 2 mM $MgCl_2$•$6H_2O$, 10 mM HEPES, 4 mM ATP, and 2 mM GTP. Data are presented as means ± standard errors of means.

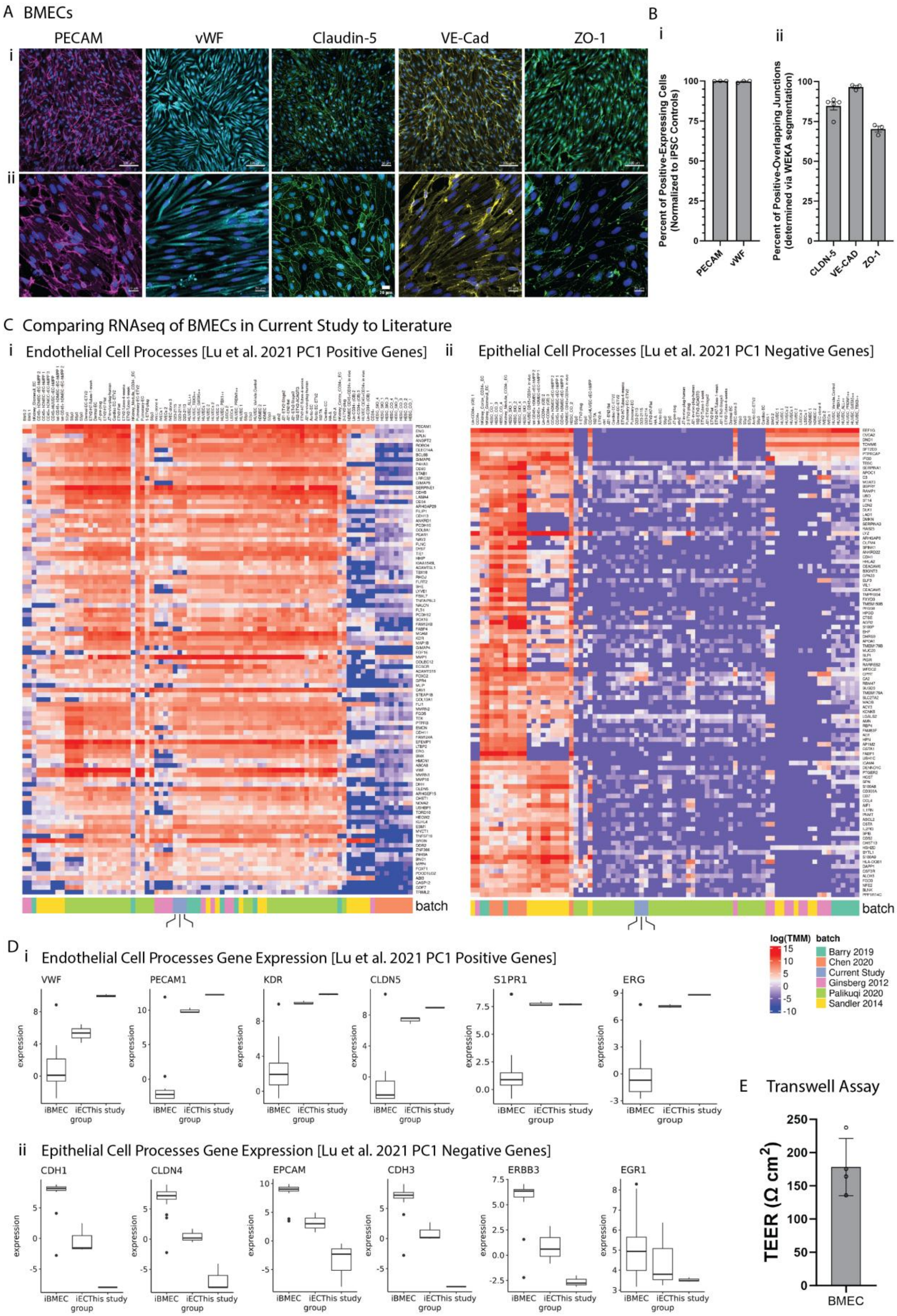
A BMECs
PECAM
vWF
Claudin-5
VE-Cad
ZO-1
i
ii
B
i
ii
Percent of Positive-Expressing Cells (Normalized to iPSC Controls)
PECAM
vWF
Percent of Positive-Overlapping Junctions (determined via WEKA segmentation)
CLDN-5
VE-CAD
ZO-1
C Comparing RNAseq of BMECs in Current Study to Literature
i Endothelial Cell Processes [Lu et al. 2021 PC1 Positive Genes]
ii Epithelial Cell Processes [Lu et al. 2021 PC1 Negative Genes]
batch
log(TMM)
batch
Barry 2019
Chen 2020
Current Study
Ginsberg 2012
Palikuqi 2020
Sandler 2014
D i Endothelial Cell Processes Gene Expression [Lu et al. 2021 PC1 Positive Genes]
VWF
PECAM1
KDR
CLDN5
S1PR1
ERG
expression
iBMEC
iEC
This study
group
ii Epithelial Cell Processes Gene Expression [Lu et al. 2021 PC1 Negative Genes]
CDH1
CLDN4
EPCAM
CDH3
ERBB3
EGR1
E Transwell Assay
TEER (Ω cm²)
BMEC

Fig. S1: Differentiation and Validation of BMECs. (**A**) iPSC-derived BMECs immunoreactivity to endothelial cell markers (**i**) lower magnification images (*left to right*): PECAM (magenta: PECAM, blue: Hoechst; scale, 100 µm), vWF (cyan: vWF, blue: Hoechst; scale, 100 µm), Claudin-5 (green: Claudin-5, blue: Hoechst; scale, 50 µm), VE-Cad (yellow: VE-Cad, blue: Hoechst; scale bar, 100 µm), ZO-1 (green: ZO-1, blue: Hoechst; scale, 100 µm), (**ii**) higher magnification images as in *(i)* (scale bars, 20 µm), (**B**) quantification of the percent of positive marker-expressing cells via immunohistochemistry for (**i**) PECAM and vWF expression above iPSC controls and (**ii**) junctional markers Claudin-5, VE-Cad, and ZO-1 expression at borders defined by PECAM (Claudin-5) or phalloidin (VE-Cad, ZO-1) reference channel via WEKA segmentation and border overlay (n ≥ 3 wells per group), (**C**) BMEC RNAseq integrated to literature datasets for (**i**) endothelial cell processes and (**ii**) epithelial cell processes determined by Lu, et al. 2021 *(33)* with the sequencing from this study (batch legend: blue; indicated with lines below) integrated with literature datasets *(33-38)* (scale: blue to red for log(TMM) of -10 to 15), (**D**) gene expression for key genes from (**i**) endothelial cell processes and (**ii**) epithelial cell processes as analyzed in Lu, et al. 2021 *(33)* and comparing literature iBMECs and iECs to the BMECs used in this study, and (**E**) TEER assay for BMEC monolayers on 0.4 µm pore size, 12 mm diameter Transwell at day 15 (n = 4).

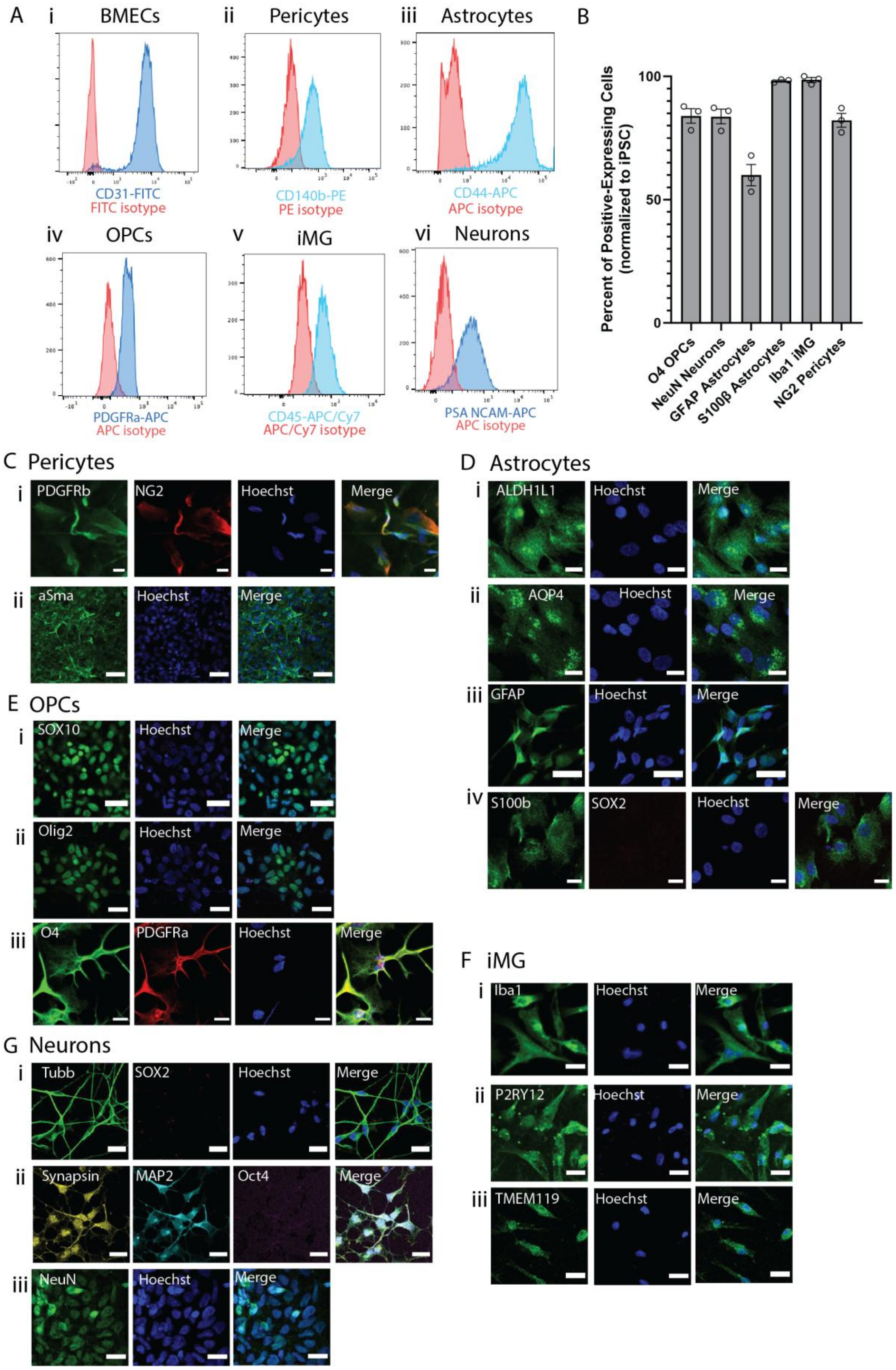

A
i BMECs
CD31-FITC
FITC isotype
ii Pericytes
CD140b-PE
PE isotype
iii Astrocytes
CD44-APC
APC isotype
iv OPCs
PDGFRa-APC
APC isotype
v iMG
CD45-APC/Cy7
APC/Cy7 isotype
vi Neurons
PSA NCAM-APC
APC isotype
B
Percent of Positive-Expressing Cells (normalized to iPSC)
100
50
0
O4 OPCs
NeuN Neurons
GFAP Astrocytes
S100β Astrocytes
Iba1 iMG
NG2 Pericytes
C Pericytes
i PDGFRb NG2 Hoechst Merge
ii aSma Hoechst Merge
D Astrocytes
i ALDH1L1 Hoechst Merge
ii AQP4 Hoechst Merge
iii GFAP Hoechst Merge
iv S100b SOX2 Hoechst Merge
E OPCs
i SOX10 Hoechst Merge
ii Olig2 Hoechst Merge
iii O4 PDGFRa Hoechst Merge
F iMG
i Iba1 Hoechst Merge
ii P2RY12 Hoechst Merge
iii TMEM119 Hoechst Merge
G Neurons
i Tubb SOX2 Hoechst Merge
ii Synapsin MAP2 Oct4 Merge
iii NeuN Hoechst Merge

Fig. S2: Differentiation and Validation of iPSC-Derived Brain Cell Types. (**A**) Flow cytometry marker analysis of (**i**) BMECs for canonical marker CD31 (blue: CD31, red: isotype control), (**ii**) iPSC-derived pericytes for CD140b (blue: CD140b, red: isotype control), (**iii**) iPSC-derived astrocytes for CD44 (blue: CD44, red: isotype control), (**iv**) iPSC-derived OPCs for PDGFRα (blue: PDGFRα, red: isotype control), (**v**) iPSC-derived iMG for CD45 (blue: CD45, red: isotype control), (**vi**) iPSC-derived neurons for excitatory cortical neuron marker PSA-NCAM (blue: PSA-NCAM, red: isotype control), (**B**) quantification of percent positive-expressing cells assessed via immunohistochemistry at the end of the differentiation protocols and two days after re-plating, compared to iPSCs stained in parallel, for key cell markers (n >= 3 wells/ marker), (**C**) representative immunofluorescence images of iPSC-derived pericytes expressing markers (**i**) PDGFRb and NG2 (green: PDGFRb, red: NG2, blue: Hoechst; scale bars, 20 μm) and (**ii**) αSMA (green: αSMA, blue: Hoechst; scale bars, 20 μm), (**D**) iPSC-derived astrocytes positive immunoreactivity to astrocyte markers (**i**) ALDH1L1 (green: ALDH1L1, blue: Hoechst; scale bar, 20 μm), (**ii**) AQP4 (green: AQP4, blue: Hoechst; scale bar, 20 μm), (**iii**) GFAP (green: GFAP, blue: Hoechst; scale bar, 20 μm), and (**iv**) S100b and absence of immunoreactivity to neuron progenitor marker SOX2 (green: S100b, red: SOX2, blue: Hoechst; scale bar, 20 μm), (**E**) iPSC-derived OPCs stained positive for markers (**i**) SOX10 (green: SOX10, blue: Hoechst; scale bar, 20 μm), (**ii**) Olig2 (green: Olig2, blue: Hoechst; scale bar, 20 μm), and (**iii**) O4 and PDGFRα (green: O4, red: PDGFRα, blue: Hoechst; scale bar, 20 μm), (**F**) iPSC-derived iMG immunoreactivity to canonical markers (**i**) Iba1 (green: Iba1, blue: Hoechst; scale bar, 20 μm), (**ii**) P2RY12 (green: P2RY12, blue: Hoechst; scale bar, 20 μm), and (**iii**) TMEM119 (green: TMEM119, blue: Hoechst; scale bar, 20 μm), and (**G**) iPSC-derived neurons immunoreactivity (**i**) to b-Tubulin and SOX2 (green: TUBB, red: SOX2, blue: Hoechst; scale bar, 20 μm), (**ii**) MAP2 and synapsin and absence of immunoreactivity to pluripotency marker OCT4 (yellow: synapsin, cyan: MAP2, magenta: OCT4, blue: Hoechst; scale bars, 20 μm), and (**iii**) NeuN (green: NeuN, blue: Hoechst; scale bar, 20 μm).

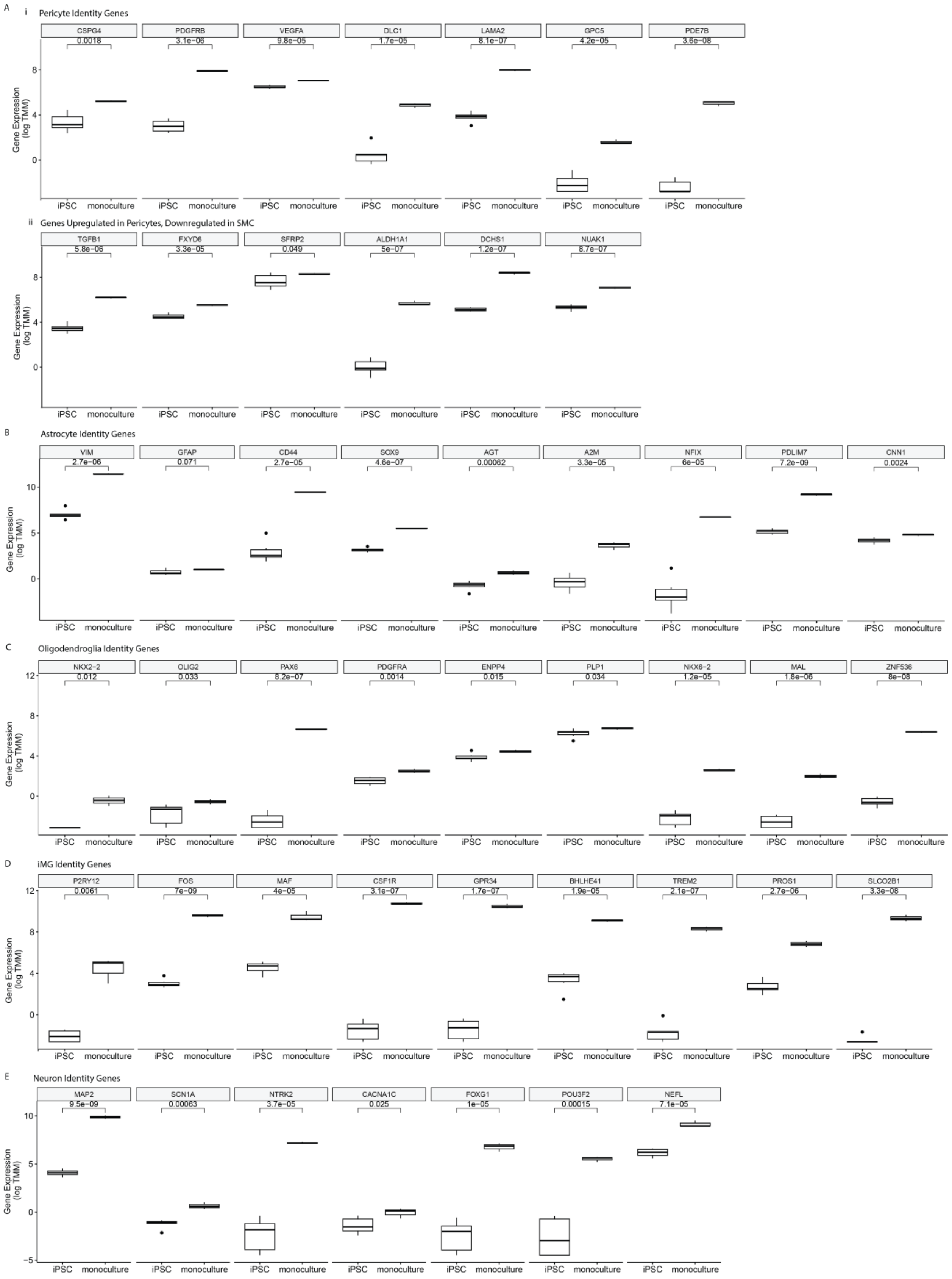
A
i Pericyte Identity Genes
CSPG4 0.0018
PDGFRB 3.1e−06
VEGFA 9.8e−05
DLC1 1.7e−05
LAMA2 8.1e−07
GPC5 4.2e−05
PDE7B 3.6e−08
ii Genes Upregulated in Pericytes, Downregulated in SMC
TGFB1 5.8e−06
FXYD6 3.3e−05
SFRP2 0.049
ALDH1A1 5e−07
DCHS1 1.2e−07
NUAK1 8.7e−07
B Astrocyte Identity Genes
VIM 2.7e−06
GFAP 0.071
CD44 2.7e−05
SOX9 4.6e−07
AGT 0.00062
A2M 3.3e−05
NFIX 6e−05
PDLIM7 7.2e−09
CNN1 0.0024
C Oligodendroglia Identity Genes
NKX2−2 0.012
OLIG2 0.033
PAX6 8.2e−07
PDGFRA 0.0014
ENPP4 0.015
PLP1 0.034
NKX6−2 1.2e−05
MAL 1.8e−06
ZNF536 8e−08
D iMG Identity Genes
P2RY12 0.0061
FOS 7e−09
MAF 4e−05
CSF1R 3.1e−07
GPR34 1.7e−07
BHLHE41 1.9e−05
TREM2 2.1e−07
PROS1 2.7e−06
SLCO2B1 3.3e−08
E Neuron Identity Genes
MAP2 9.5e−09
SCN1A 0.00063
NTRK2 3.7e−05
CACNA1C 0.025
FOXG1 1e−05
POU3F2 0.00015
NEFL 7.1e−05
Gene Expression (log TMM)
iPSC
monoculture

Fig. S3: Gene Expression Validation of iPSC-Derived Brain Cell Types. (**A**) Gene expression for iPSCs and pericyte monocultures for (**i**) key pericyte identity genes and (**ii**) genes that have been found to be upregulated in pericytes and downregulated in SMCs, (**B**) gene expression for iPSCs and astrocyte monocultures for astrocyte identity genes, (**C**) gene expression for iPSCs and OPC monocultures for OPC and oligodendrocyte identity genes, (**D**) gene expression for iPSCs and iMG monocultures for microglial identity genes, and (**E**) gene expression for iPSCs and neuron monocultures for neuron identity genes (expression plotted as log TMM; statistical significance determined via t test).

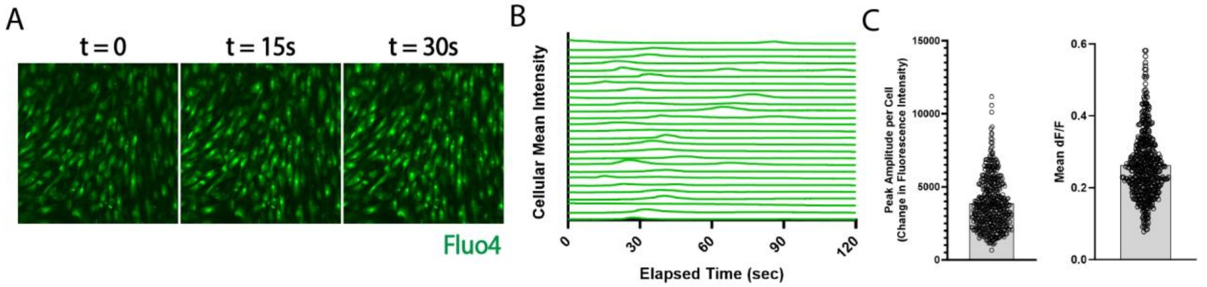

Fig. S4: Characterization of Astrocyte Calcium Transients. (**A**) Images of calcium transients recorded from astrocyte monocultures with calcium indicator Fluo4, (**B**) representative traces of calcium transients plotting mean intensity over time, and (**C**) quantification of (left) peak amplitude per cell in terms of fluorescence intensity and (right) mean dF/F for n = 485 cells.

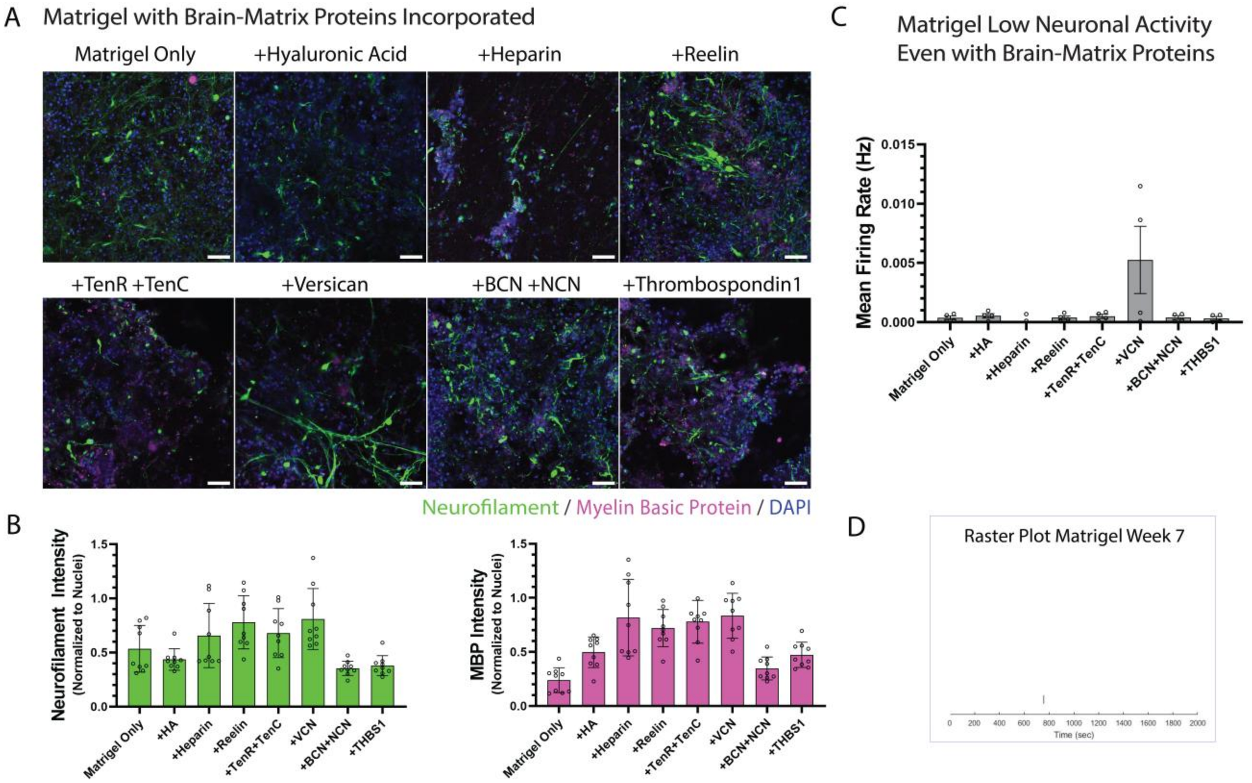

Fig. S5: Screening Brain Extracellular Matrix Components in Matrigel Co-Culture. (**A**) Neuronal morphology (green: neurofilament) and myelination (magenta: MBP) in miBrains encapsulated in Matrigel incorporated with various brain extracellular matrix components (scale bars, 50 µm), (**B**) average intensity of neuronal (top) and MBP immunoreactivity (bottom) across miBrains encapsulated in Matrigel (data from n = 3 replicates, n = 3 fields of view each; plotted as mean and S.D.), (**C**) neuronal activity as measured on an MEA at week 7 for mean firing rate (data from n = 4 replicates, averaged over recordings of at least 30 min.; plotted as mean and S.E.M.), and (**D**) raster plot for spikes of neuronal activity in a representative miBrain in Matrigel at week 7.

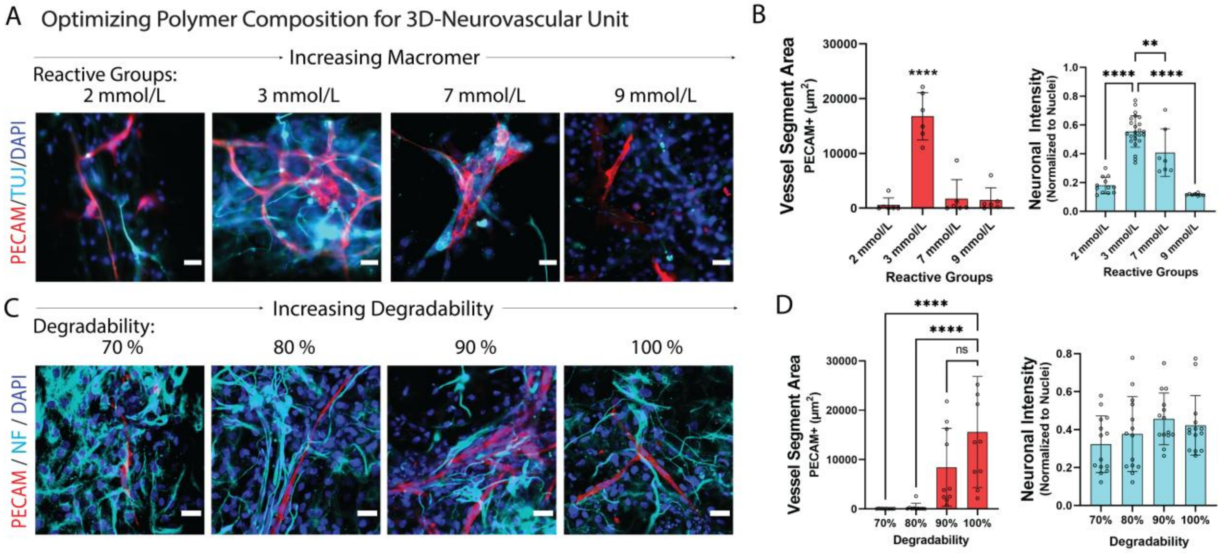

Fig. S6: Optimizing Polymer Scaffold for miBrain Culture. (**A**) Optimization of mechanical properties of dextran-based hydrogels across amounts of macromer and crosslinker reactive groups, screening across 3D, integral neurovascular network co-assembly for vessels (red: PECAM) and neurons (cyan: Tuj1) with single z-plane images (scale bars, 30 μm), (**B**) quantified in terms of area of the segments of vessels (PECAM+) captured in images (left; data from n = 3 replicates, n = 6 field of view, mean and S.D.; statistical analysis via one-way ANOVA, **** $p < 0.0001$) and the Tuj1 immunoreactivity (right; data from n = 3 replicates, n = 7 fields of view; statistical analysis via one-way ANOVA, mean and S.D., ** $p = 0.006$, **** $p < 0.0001$), (**C**) varying degradability in dextran-based hydrogels, screening across 3D, integral neurovascular network co-assembly for vessels (red: PECAM) and neurons (cyan: Tuj1) with single z-plane images (scale bars, 30 μm), and (**D**) quantified in terms of area of the segments of vessels (PECAM+) captured in images (left; data from n = 3 replicates, n = 10 fields of view, mean and S.D.; statistical analysis via one-way ANOVA, **** $p < 0.0001$) and the Tuj1 immunoreactivity (right; data from n = 3 replicates, n = 3 fields of view each, mean and S.D.).

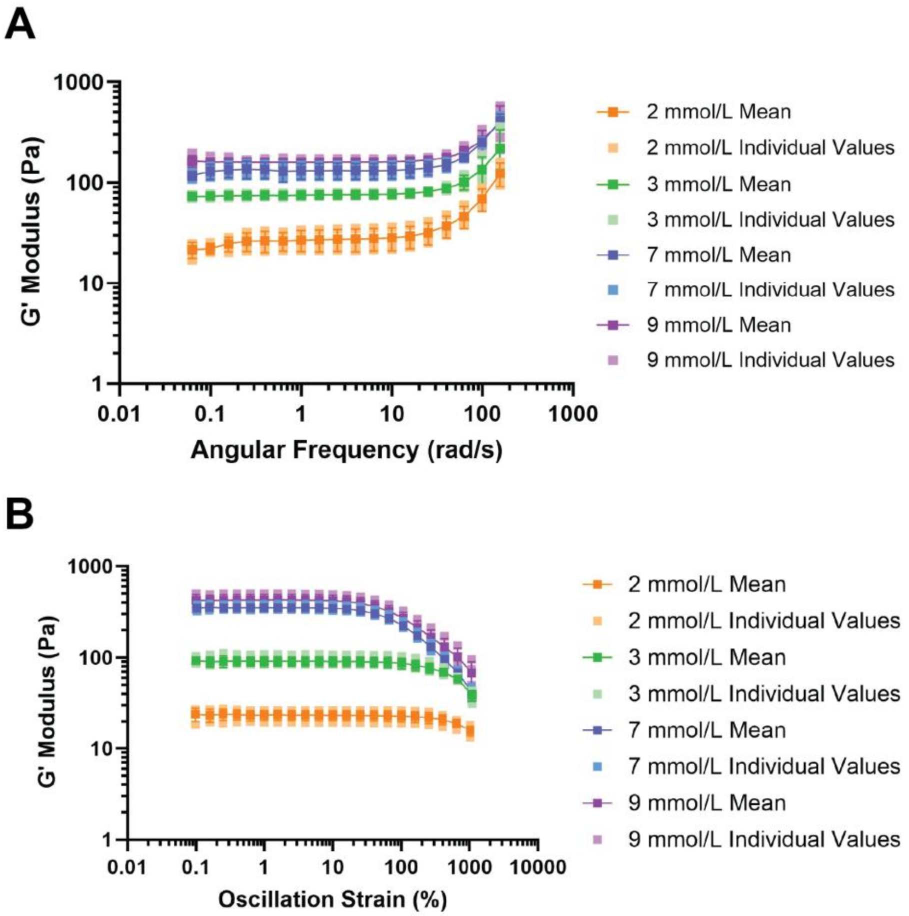

Fig. S7: Rheological Measurements of Dextran-Based Hydrogels. (**A**) Logarithmic frequency sweep applying 1% strain (n = 3) and (**B**) logarithmic strain sweep applying angular frequency of 0.1 Hz (n = 4) plotted as individual values (light) and mean with standard deviation (dark) for the storage modulus (G').

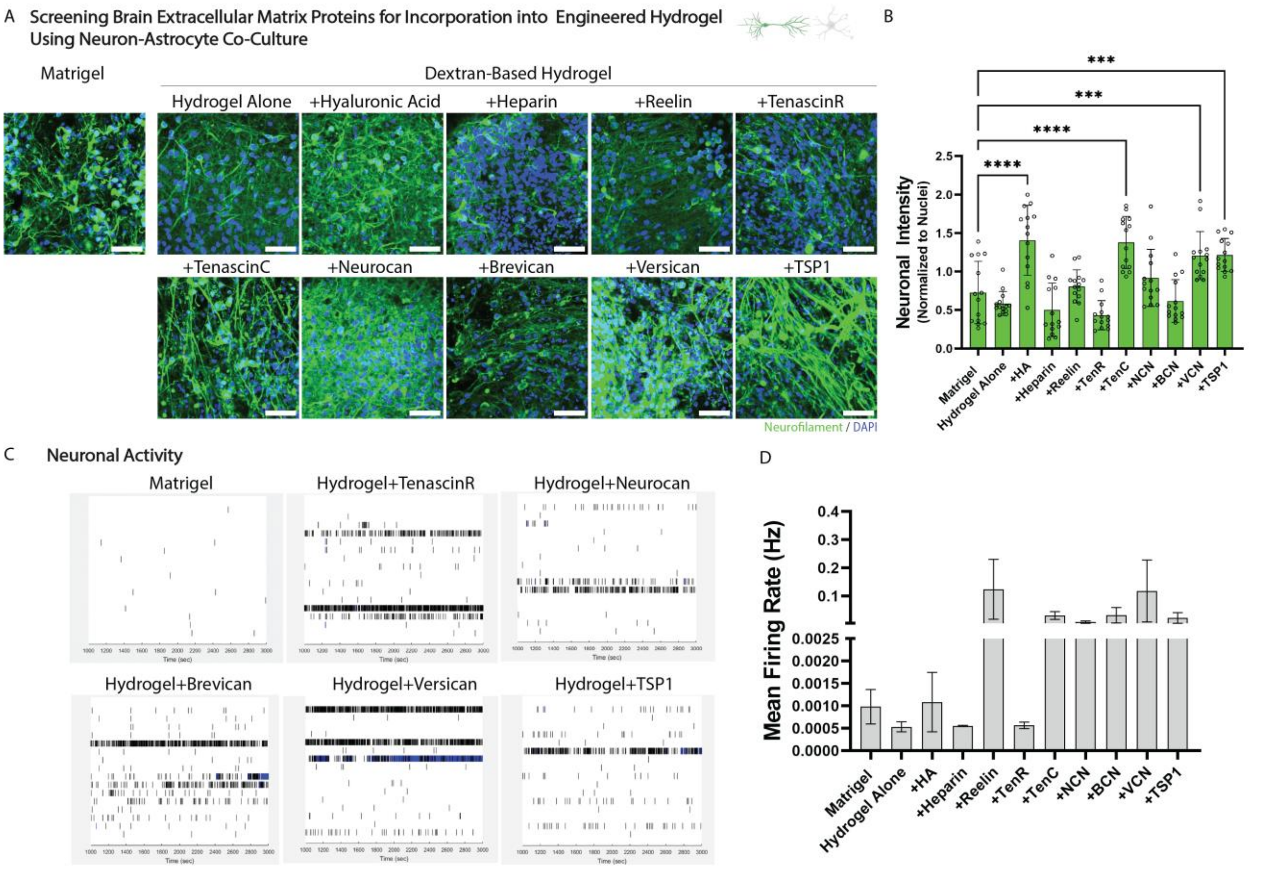

Fig. S8: Screening Brain Extracellular Matrix Components Incorporated into Engineered Hydrogel in Simplified Co-Culture. (**A**) Neuronal morphology (green: neurofilament) after 2 weeks in a neuron-astrocyte co-culture used to screen across dextran-based engineered hydrogels incorporated with various brain extracellular matrix components (scale bars, 50 μm), (**B**) average intensity of neuronal immunoreactivity across conditions (data from n = 3 replicates, n = 14 fields of view; statistical analysis via one-way ANOVA, *** p = 0.006, **** p < 0.0001), (**C**) example raster plots of neuronal activity assessed on an MEA system early in the culture at week 2, and (**D**) averaged mean firing rate at week 2 across extracellular matrix conditions (data are from n = 3 replicates, averaged over recordings of at least 30 min.; mean and S.E.M.).

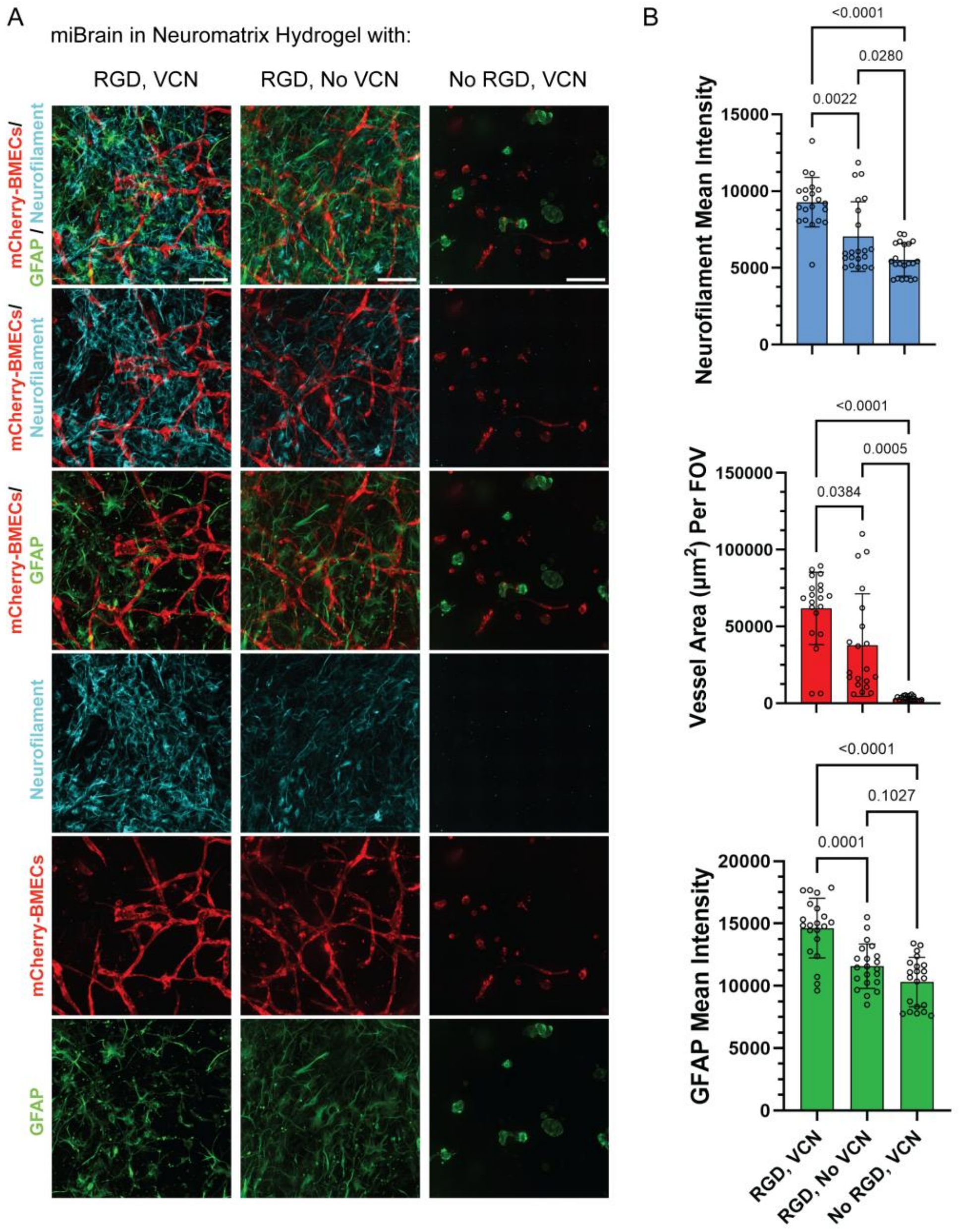

Fig. S9: Effects of Neuromatrix Hydrogel Components. (**A**) miBrains constructed (*left*) with all hydrogel components, inclusive of RGD and VCN, (*middle*) with RGD, but no VCN, and (*right*) with no RGD and no VCN (red: mCherry-BMECs, cyan: neurofilament, green: GFAP; scale bars, 100 μm) and (**B**) quantification of (*top*) neurofilament mean intensity (n = 21 fields of view), (*middle*) vessel area (n = 20 fields of view), and (*bottom*) GFAP mean intensity (n = 21 fields of view) (Brown-Forsythe and Welch ANOVA statistical test).

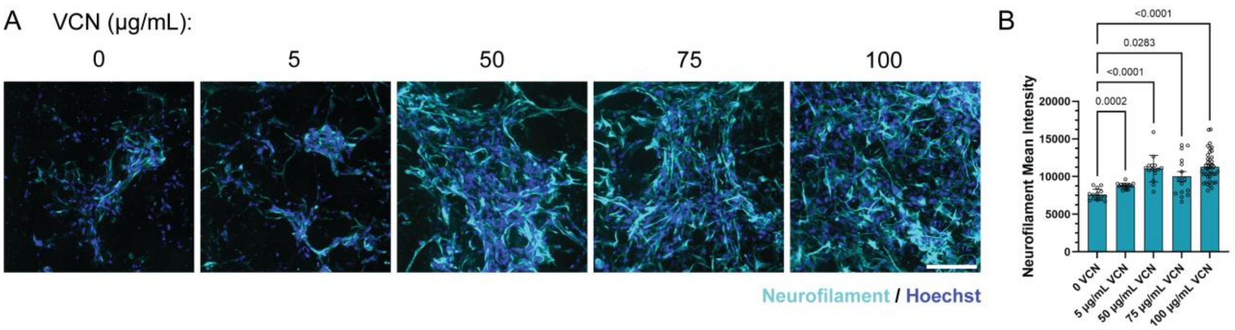

Fig. S10: Assessing Dose of VCN. (**A**) miBrains constructed with 0, 5, 50, 75, or 100 µg/mL VCN (cyan: neurofilament, blue: Hoechst; scale bar, 100 µm) and (**B**) quantification of neurofilament mean intensity (n = 15 fields of view, Brown-Forsythe and Welch ANOVA statistical test).

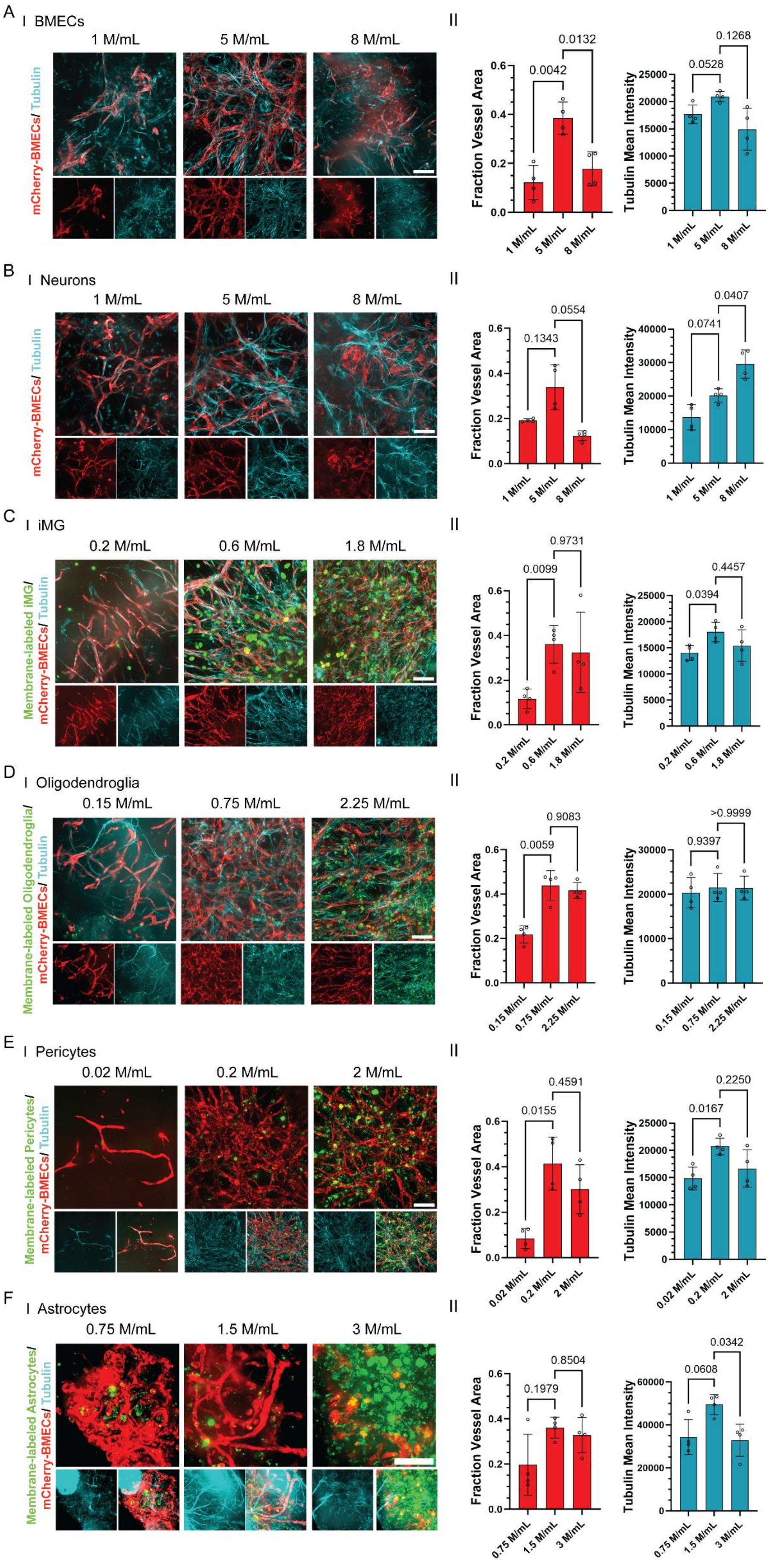
A
I BMECs
1 M/mL
5 M/mL
8 M/mL
mCherry-BMECs/ Tubulin
II
Fraction Vessel Area
0.0042
0.0132
Tubulin Mean Intensity
0.0528
0.1268
B
I Neurons
0.1343
0.0554
0.0741
0.0407
C
I iMG
0.2 M/mL
0.6 M/mL
1.8 M/mL
Membrane-labeled iMG/ mCherry-BMECs/ Tubulin
0.0099
0.9731
0.0394
0.4457
D
I Oligodendroglia
0.15 M/mL
0.75 M/mL
2.25 M/mL
Membrane-labeled Oligodendroglia/ mCherry-BMECs/ Tubulin
0.0059
0.9083
0.9397
>0.9999
E
I Pericytes
0.02 M/mL
0.2 M/mL
2 M/mL
Membrane-labeled Pericytes/ mCherry-BMECs/ Tubulin
0.0155
0.4591
0.0167
0.2250
F
I Astrocytes
0.75 M/mL
1.5 M/mL
3 M/mL
Membrane-labeled Astrocytes/ mCherry-BMECs/ Tubulin
0.1979
0.8504
0.0608
0.0342

Fig. S11: Example of Optimizing Cell Ratios. miBrains with each component cell of varied concentration: (i) representative images (red: mCherry-BMECs, cyan: tubulin; scale bar, 100 μm) and (ii) quantification of fraction of vessel area (*left*) and tubulin mean intensity (*right*) (n = 4 fields of view, Brown-Forsythe and Welch ANOVA statistical test) (**A**) BMECs at concentrations of 1, 5, or 8 M/mL, (**B**) neurons at concentrations of 1, 5, or 8 M/mL, (**C**) iMG at concentrations of 0.2, 0.6, or 1.8 M/mL (green: membrane-labeled iMG), (**D**) oligodendroglia at concentrations of 0.15, 0.75, or 2.25 M/mL (green: membrane-labeled oligodendroglia), (**E**) pericytes at concentrations of 0.02, 0.2, and 2 M/mL (green: membrane-labeled pericytes), and (**F**) astrocytes at concentrations of 0.75, 1.5, and 3 M/mL (green: membrane-labeled astrocytes).

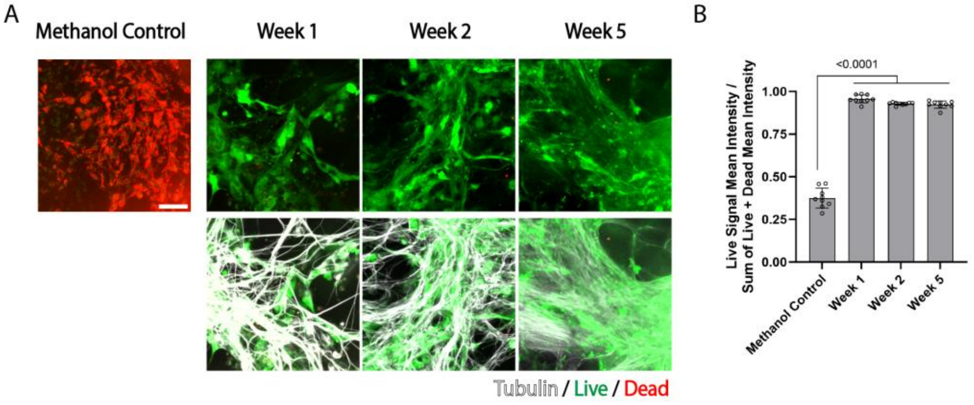

Fig. S12: miBrain Cell Composition. (**A**) live/dead assay for miBrains at weeks 1, 2, and 5 (*top*) compared to methanol control (green: live, red: dead; scale bar, 100 μm) and (*bottom*) co-labeled with tubulin live dye to visualize neurons (gray: tubulin, green: live, red: dead) and (**B**) live cell fraction in each condition (n = 3 fields of view from n = 3 miBrains/ group, repeated in 3 independent batches; statistical significance determined via t test).

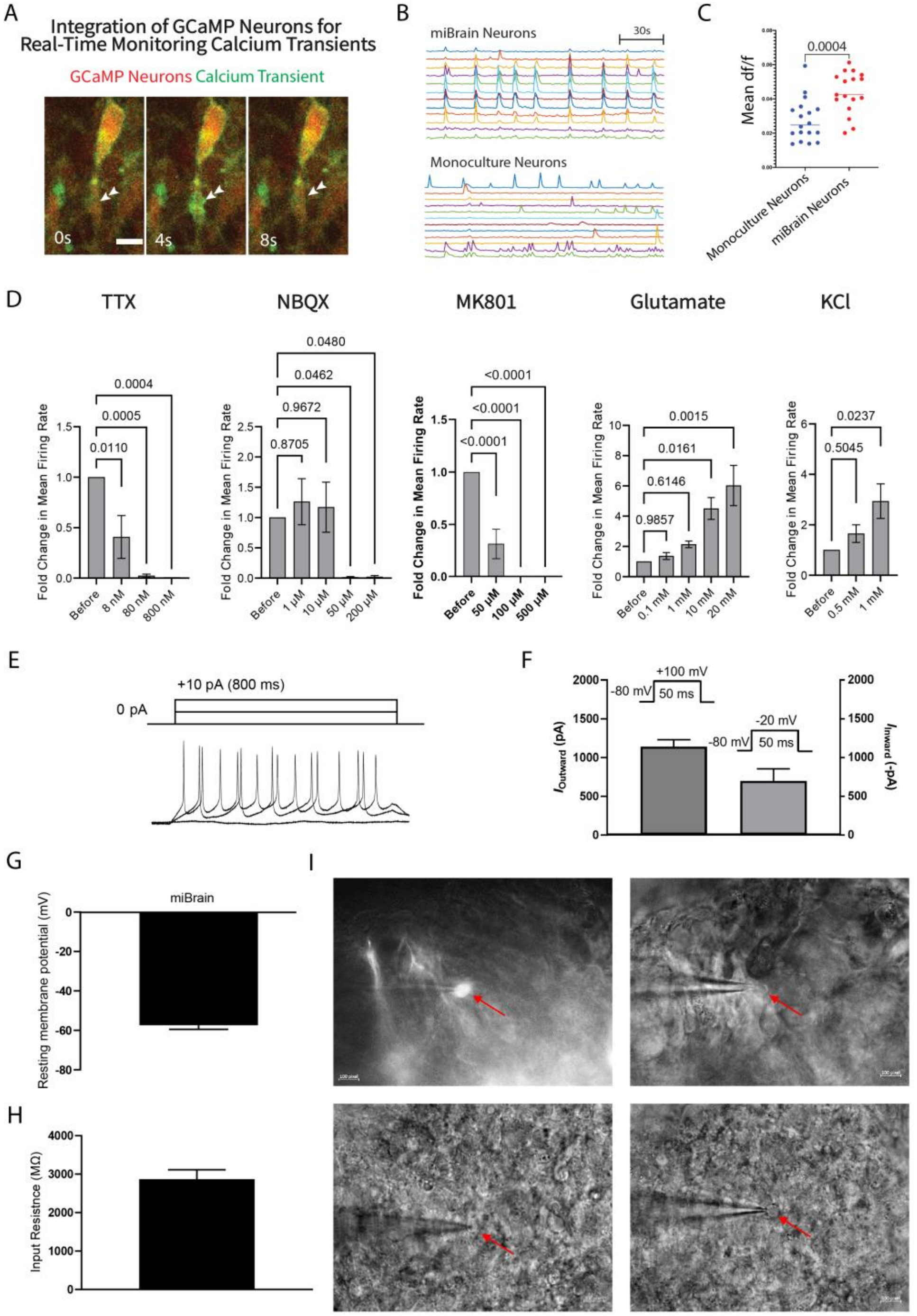

Fig. S13: Neuromodulator and Electrophysiological Analysis of miBrain Neurons. (**A**) example images of calcium dynamics in GCaMP neurons (red: tdTomato GCaMP neurons, green: calcium transient; scale bar, 25 µm), (**B**) example spike traces of neuronal calcium transients in miBrain (left) compared to monoculture (right) (scale bar, 30s), (**C**) quantification of calcium events plotted as max dF/F per calcium trace per neuron recorded (statistical analysis via student t test p = 0.0004), (**D**) Change in mean firing rate for miBrains in response to varied doses of neuromodulators assessed via MEA for (*from left to right*) TTX, NBQX, MK801, MNI-caged-L-Glutamate, and KCl (n = 4 miBrains/ group; plotted as mean and S.E.M.; statistical significance determined via one-way ANOVA), (**E**) representative whole-cell current clamp recording of action potentials from miBrain-neurons, (**F**) summary of whole-cell voltage clamp recording of inward and outward currents (n = 31 cells), (**G**) summary of resting membrane potentials (n = 31 cells), (**H**)

summary of input resistance (n = 31 cells), and (**I**) images of whole-cell patch clamp recording of miBrain-neurons.

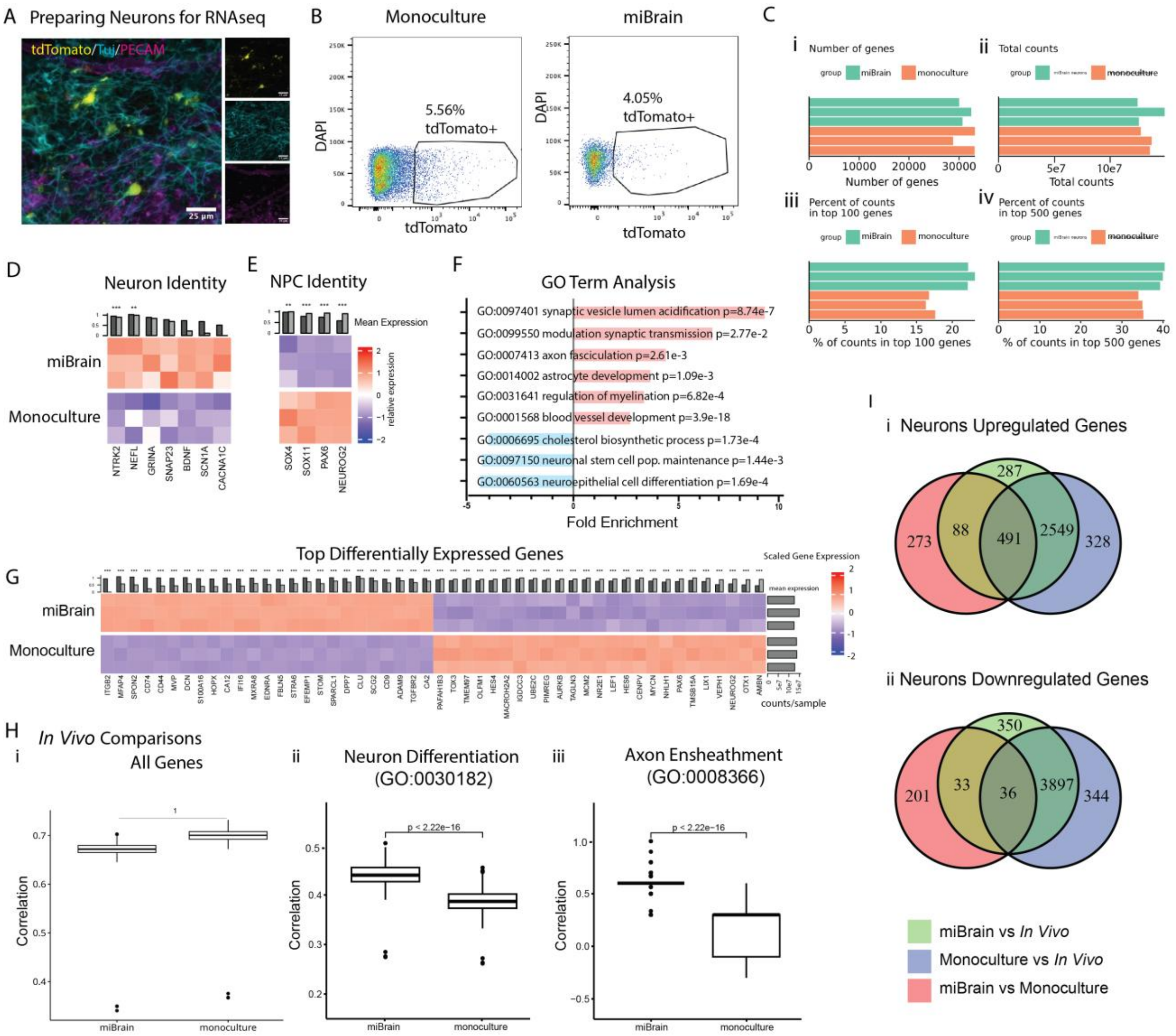

Fig. S14: Transcriptomic Analysis of miBrain-Neurons. (**A**) tdTomato-neurons incorporated into miBrains enabling tdTomato-based isolation of neurons via flow cytometry (yellow: tdTomato, magenta: PECAM, cyan: Tuj; scale bars, 25 μm), (**B**) gating of tdTomato-positive cells in flow cytometry (**C**) RNA-sequencing of neurons in miBrains and neurons monocultures: (**i**) genes per sample, (**ii**) total gene counts, (**iii**) percent of gene counts in the top 100 genes, and (**iv**) percent of gene counts in the top 500 genes, (**D**) upregulation in the miBrain-neurons of genes associated with neuron identity, (**E**) downregulation upregulation in the miBrain-neurons of genes associated with NPC identity, (**F**) top DEGs between neurons isolated from miBrains versus monocultures sorted by mean expression in miBrain, plotting TMM-normalized and scaled expression (FDR < 0.001), (**G**) gene ontology analysis of iMG RNAseq for biological pathways significantly altered in miBrain-cultured iMG based on significantly upregulated and downregulated DEGs and with an FDR p-value less than 0.05, (**H**) correlation of miBrain- and monoculture-neurons to human *in vivo* prefrontal cortex excitatory neurons identified via snRNAseq of non-AD decedent tissue *(47)* for the (**i**) whole genome, (**ii**) genes associated with neuron differentiation (GO:0030182), and (**iii**) genes associated with axon ensheathment (GO:0008366) (statistical significance determined via t test), and (**I**) Venn diagrams displaying DEGs in neurons between miBrain versus human *in vivo* (green), monoculture versus human *in vivo* (blue), and miBrain versus monoculture (red) for (i) upregulated and (ii) downregulated DEGs.

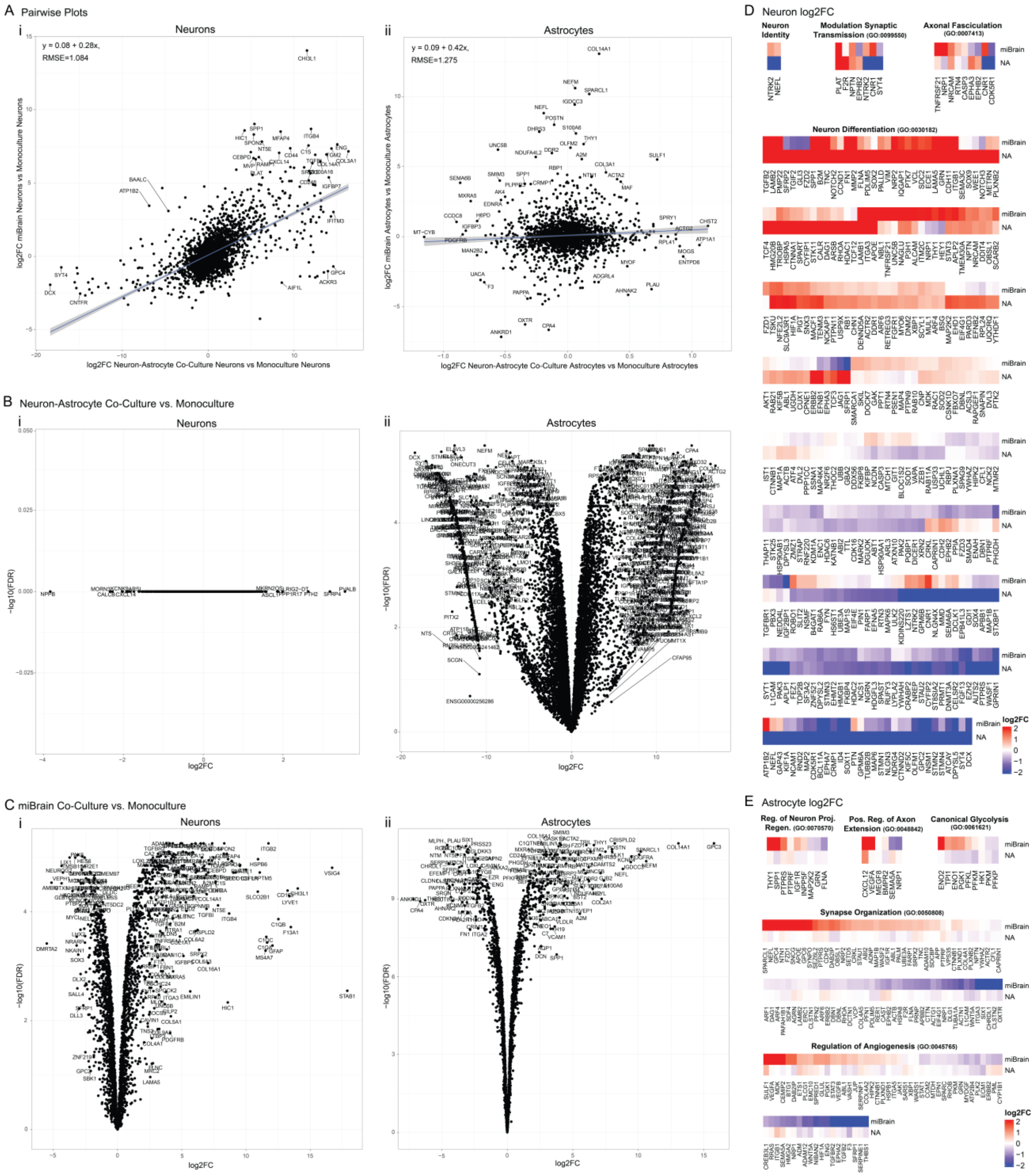

Fig. S15: Comparison of RNAseq Signatures from miBrain and Neuron-Astrocyte Co-Culture. (**A**) Pairwise plots of gene expression changes in co-culture conditions, miBrain (y-axis) in this study or neuron-astrocyte co-cultures (x-axis) in Das, et al.,[12] compared to monocultured cells for the respective datasets for (**i**) neurons and (**ii**) astrocytes, expressed as log2FC, (**B**) volcano plots of differences in gene expression for neuron-astrocyte co-culture versus monocultured cells in Das, et al., for (**i**) neurons and (**ii**) astrocytes, expressed as log2FC with significance calculated as -log10(FDR), (**C**) volcano plots of differences in gene expression in miBrain co-culture versus monocultured cells in this study for (**i**) neurons and (**ii**) astrocytes, expressed as log2FC with significance calculated as -log10(FDR), (**D**) heatmaps of gene expression changes as log2FC in co-culture conditions, miBrain in this study or neuron-astrocyte co-cultures (NA) in Das, et al., versus monocultured cells for neurons in genes associated with neuron identity, modulation of synaptic transmission (GO:0099550), axonal fasciculation

(GO:0007413), neuron differentiation (GO:0030182), and (**E**) astrocytes for regulation of neuronal projection regeneration (GO:0070570), positive regulation of axon extension (GO:0048842), canonical glycolysis (GO:0061621), synapse organization (GO:0050808), and regulation of angiogenesis (GO:0045765).

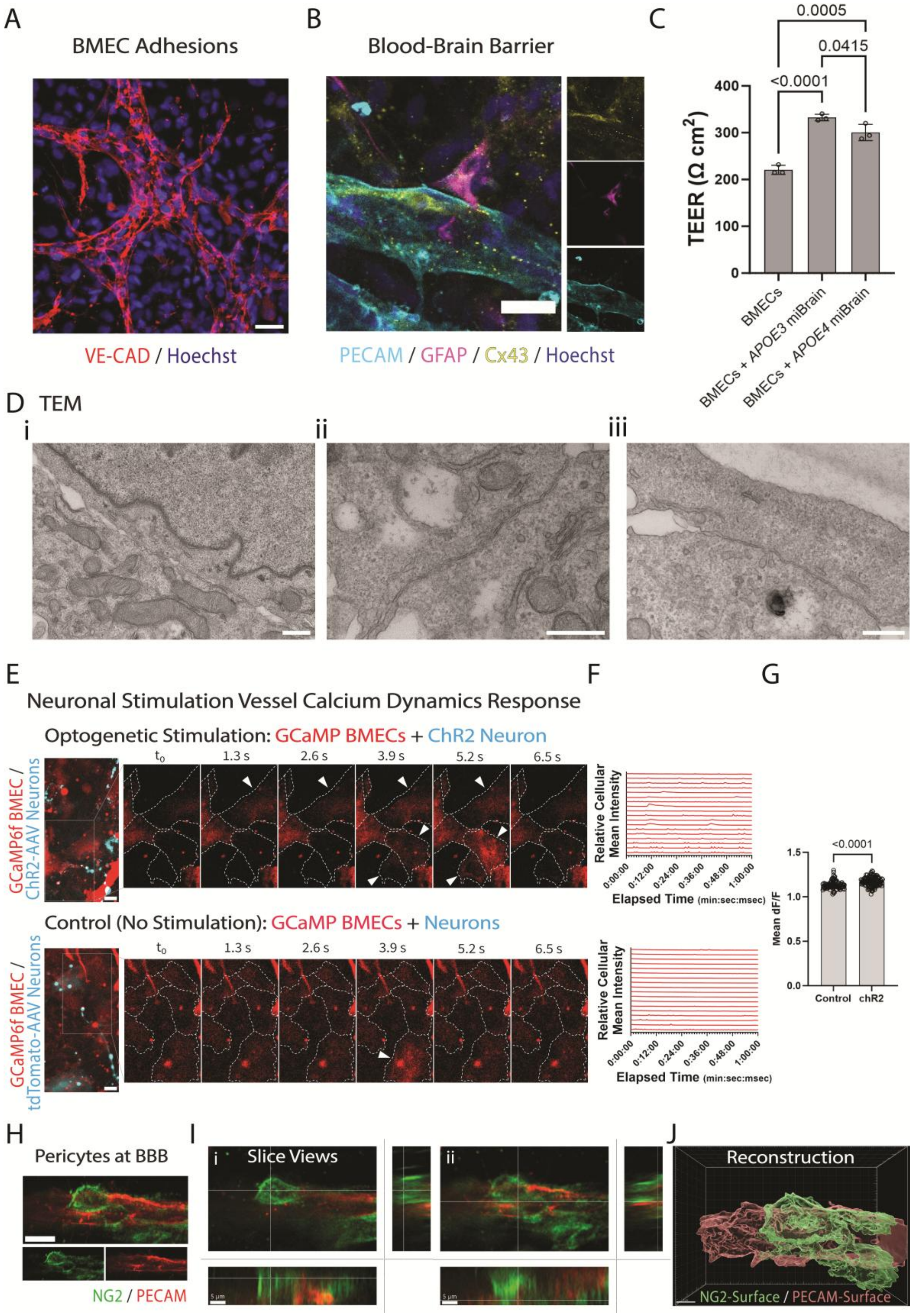

Fig. S16: BBB Characterization. (**A**) VE-CAD adhesions at vessels (red: VE-CAD, blue: Hoechst; scale bar, 30 μm), (**B**) astrocytes at microvasculature and BBB receptor immunohistochemistry at vessels

(yellow: Cx43, cyan: PECAM, magenta: GFAP, blue: Hoechst; scale bar, 25 µm), (**C**) characterization of TEER for BMEC monocultures versus BMEC monocultures seeded with *APOE3* miBrains versus BMEC monocultures seeded with *APOE4* miBrains, measured 6 days following miBrain seeding (day 10 in culture overall; n = 3/ group; statistical significance determined via one-way ANOVA), (**D**) TEM example images (i-iii) of tight junctions (Scale bars, 500 nm), (**E**) assessing neurovascular coupling via BMEC calcium transients in response to neuronal activity (top) optogenetically-driven (cyan: ChR2-mCherry-neurons) versus (bottom) baseline (cyan: tdTomato-neurons) and (right) sequential images of calcium transients in BMECs imaged under blue light (red: GCaMP6f-BMECs; scale bars, 10 µm), (**F**) corresponding example spike traces, (**G**) quantification of calcium events (mean dF/F per calcium trace per BMEC recorded; n = 3 miBrains/ group, repeated in 3 independent trials; statistical analysis via student t test $p < 0.0001$), (**H**) pericytes in miBrain at the microvasculature (green: NG2, red: PECAM; scale bar, 10 µm), (**I**) slice and side views for z-planes at the (i) top and (ii) middle (green: NG2, red: PECAM; scale bars, 5 µm), and (**J**) corresponding 3D reconstruction (green: NG2-Surface, red: PECAM-Surface; scale bar, 4 µm).

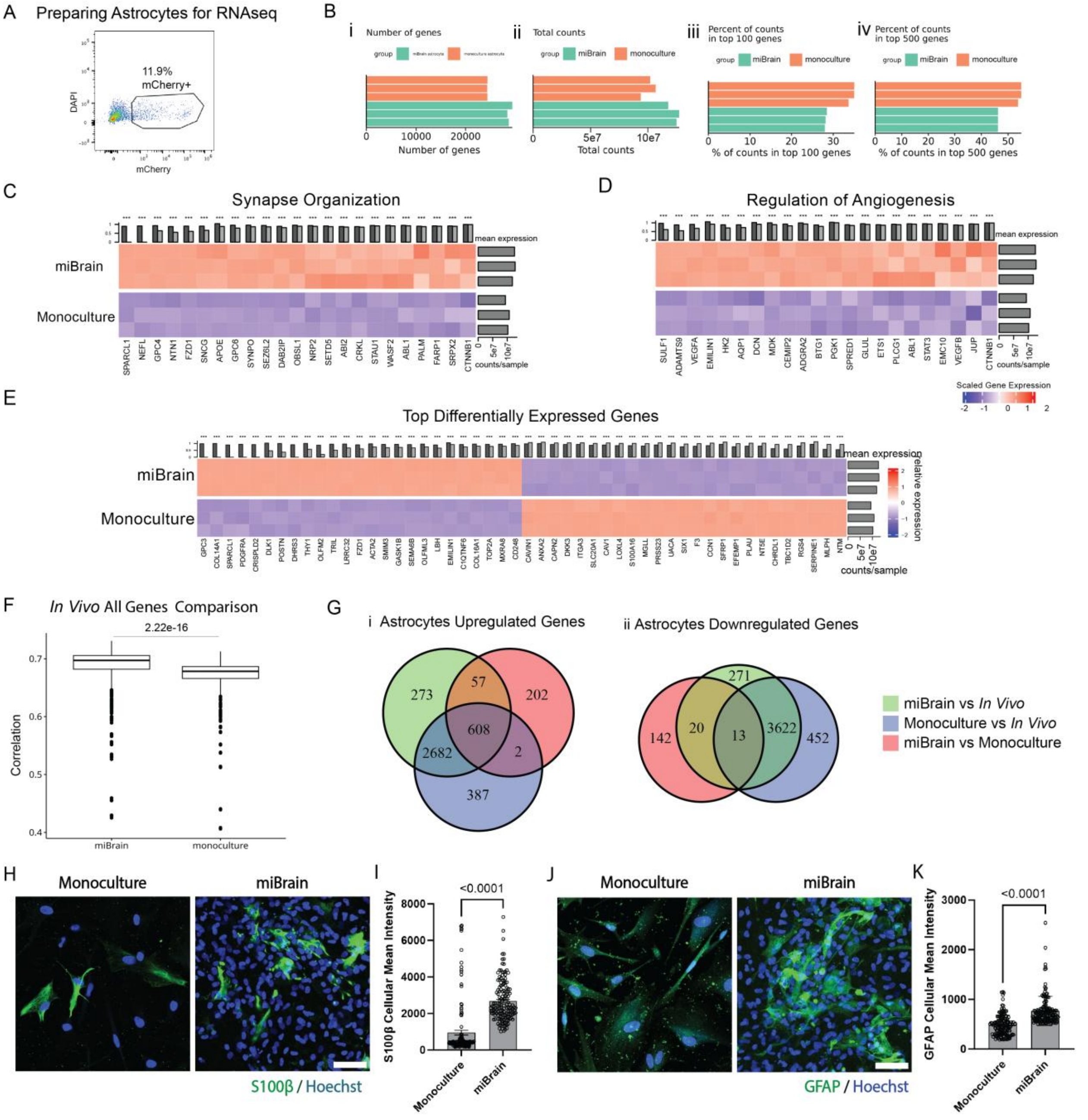

Fig. S17: Transcriptomic Analysis of miBrain-Astrocytes. (**A**) Gating of mCherry-positive cells in flow cytometry to identify astrocytes, (**B**) RNA-sequencing of astrocytes in miBrains and astrocyte monocultures: (**i**) genes per sample, (**ii**) total gene counts, (**iii**) percent of gene counts in the top 100 genes, and (**iv**) percent of gene counts in the top 500 genes, (**C**) upregulation in the miBrain-astrocytes of genes associated with synapse organization (GO:0050808; * $p < 0.05$, ** $p < 0.01$, *** $p < 0.001$), (**D**) upregulation in the miBrain-astrocytes of genes associated with regulation of angiogenesis (GO:0045765; * $p < 0.05$, ** $p < 0.01$, *** $p < 0.001$), (**E**) top DEGs between astrocytes isolated from miBrains versus monocultures sorted by mean expression in miBrain, plotting TMM-normalized and scaled expression (FDR < 0.001), and (**F**) correlation of miBrain- and monoculture-astrocytes to human *in vivo* prefrontal cortex astrocytes identified via snRNAseq of non-AD decedent tissue referencing the whole genome *(47)* (statistical significance determined via t test), (**G**) Venn diagrams displaying DEGs in astrocytes between miBrain versus human *in vivo* (green), monoculture versus human *in vivo* (blue), and miBrain versus monoculture (red) for (i) upregulated and (ii) downregulated DEGs, (**H**) immunoreactivity to S100b in astrocyte monocultures compared to miBrains (green: S100b, blue: Hoechst; scale bar, 50 µm), (**I**)

quantification of mean intensity of S100b-positive cells (n = 150 cells across n = 3 wells/ group; reported as mean and S.E.M.; statistical significance determined via t test), (**J**) immunoreactivity to GFAP in astrocyte monocultures compared to miBrains (green: GFAP, blue: Hoechst; scale bar, 50 μm), and (**K**) quantification of mean intensity of GFAP-positive cells (n = 150 cells across n = 3 wells/ group; reported as mean and standard deviation; statistical significance determined via t test).

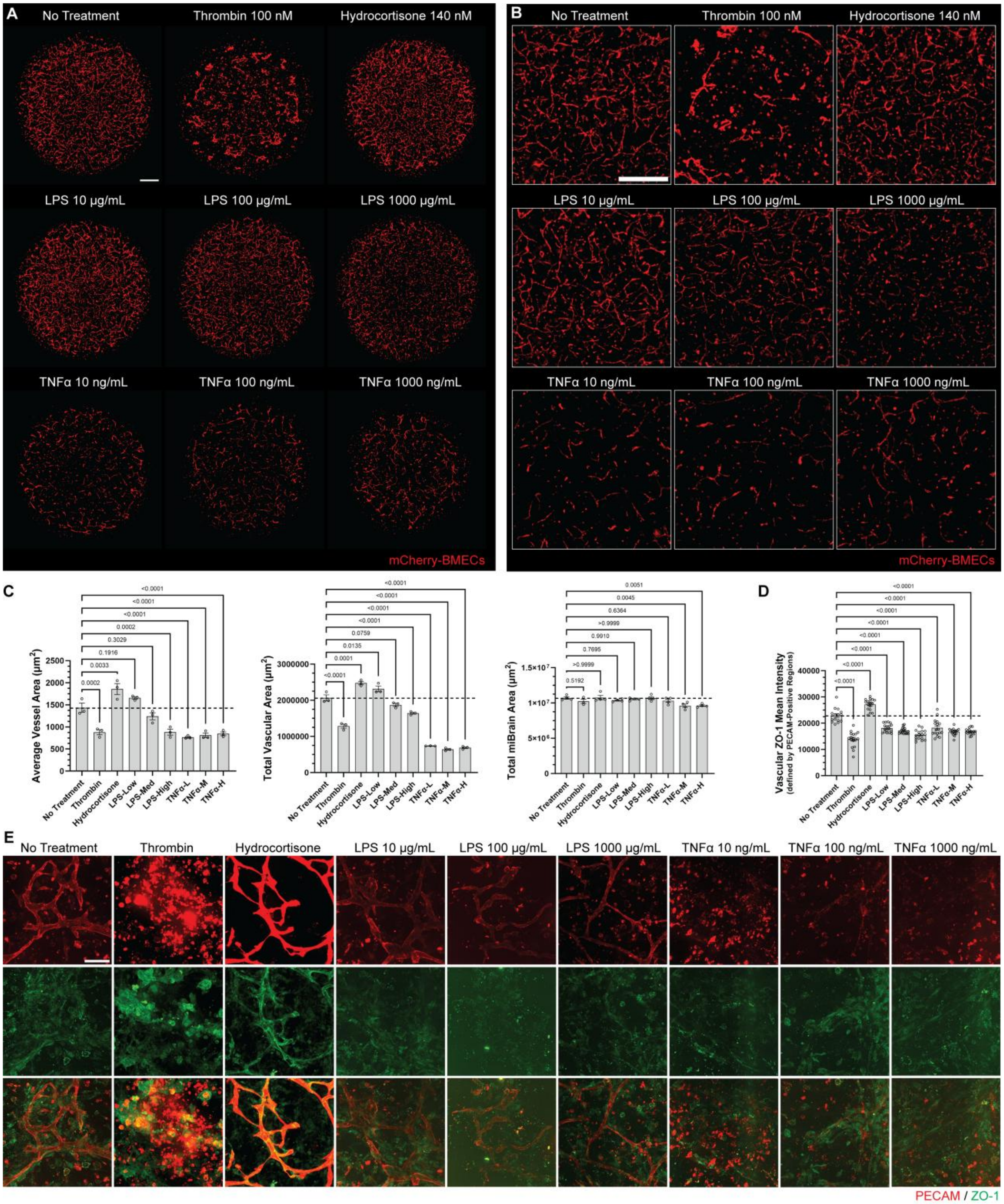

Fig. S18: Functional Barrier Assessment with BBB Modulators. (**A**) Whole-miBrain images and (**B**) magnified views of miBrain vasculature following 24 hour treatment with 100 nM thrombin, 140 nM hydrocortisone, 10, 100, or 1000 μg/mL LPS, and 10, 100, or 1000 ng/mL TNFα (red: mCherry-BMECs; scale bars, 500 μm), (**C**) quantification of (left) average vessel area, (middle) total vascular area, and (right) total miBrain area (n = 3/ group; statistical significance determined via one-way ANOVA), (**D**) quantification of ZO-1 mean signal intensity within vessel regions across treatment groups at 48 hours (n >= 15 ROIs/ group; statistical significance determined via one-way ANOVA), and (**E**) corresponding

representative images across treatment groups at 48 hours (red: PECAM, green: ZO-1; scale bar, 200 μm).

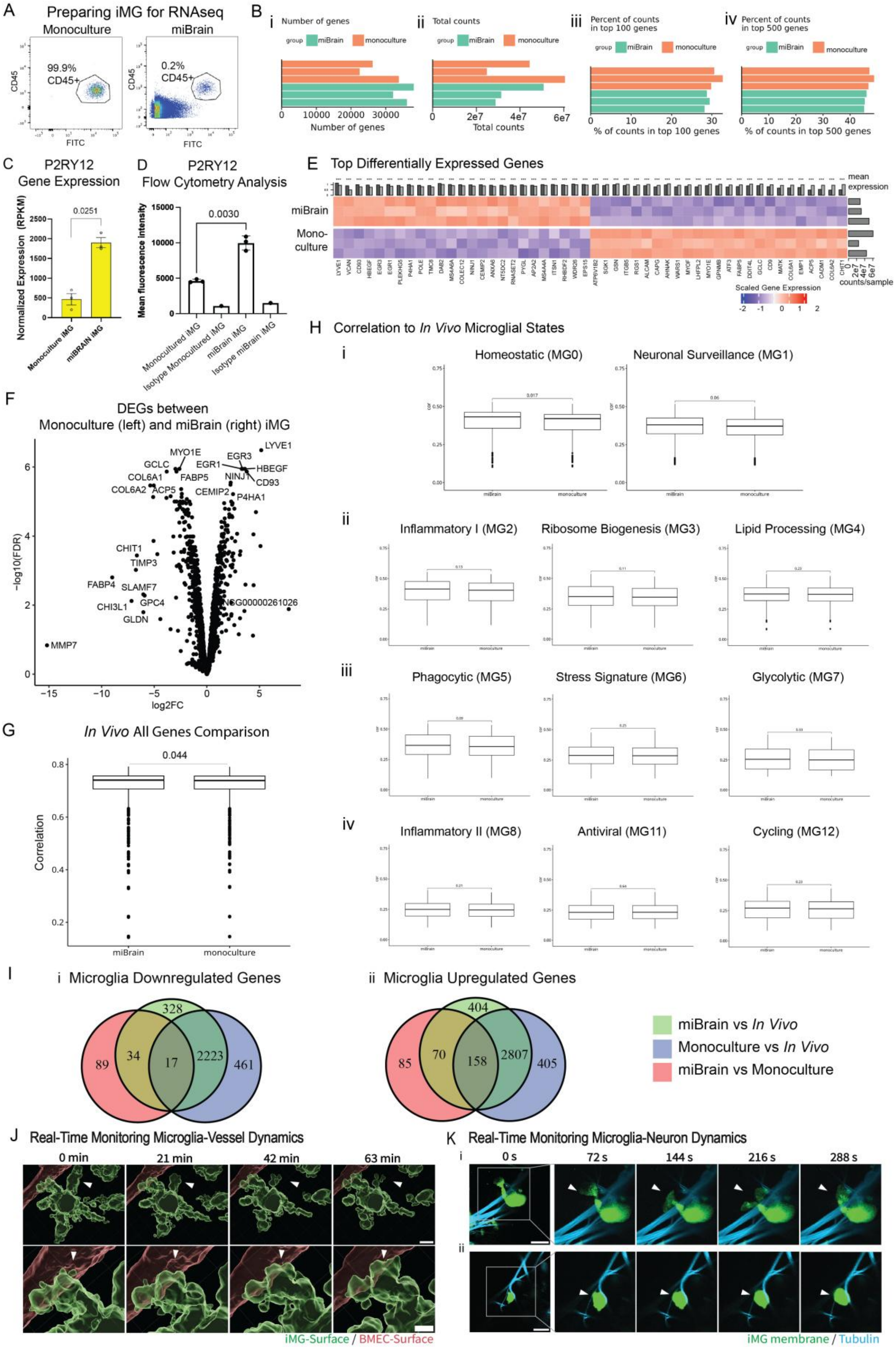
A
Preparing iMG for RNAseq
Monoculture
miBrain
99.9% CD45+
0.2% CD45+
CD45
FITC
B
i Number of genes
ii Total counts
iii Percent of counts in top 100 genes
iv Percent of counts in top 500 genes
group miBrain monoculture
Number of genes
Total counts
% of counts in top 100 genes
% of counts in top 500 genes
C
P2RY12 Gene Expression
Normalized Expression (RPKM)
0.0251
Monoculture iMG
miBRAIN iMG
D
P2RY12 Flow Cytometry Analysis
Mean fluorescence intensity
0.0030
Monocultured iMG
Isotype Monocultured iMG
miBrain iMG
Isotype miBrain iMG
E
Top Differentially Expressed Genes
mean expression
miBrain
Mono-culture
counts/sample
Scaled Gene Expression
F
DEGs between Monoculture (left) and miBrain (right) iMG
-log10(FDR)
log2FC
LYVE1
MYO1E
EGR3
GCLC
EGR1
HBEGF
COL6A1
FABP5
NINJ1
CD93
COL6A2
ACP5
CEMIP2
P4HA1
CHIT1
TIMP3
FABP4
SLAMF7
CHI3L1
GPC4
GLDN
ENSG00000261026
MMP7
G
In Vivo All Genes Comparison
0.044
Correlation
miBrain
monoculture
H
Correlation to In Vivo Microglial States
i
Homeostatic (MG0)
Neuronal Surveillance (MG1)
ii
Inflammatory I (MG2)
Ribosome Biogenesis (MG3)
Lipid Processing (MG4)
iii
Phagocytic (MG5)
Stress Signature (MG6)
Glycolytic (MG7)
iv
Inflammatory II (MG8)
Antiviral (MG11)
Cycling (MG12)
I
i Microglia Downregulated Genes
328
89
34
17
2223
461
ii Microglia Upregulated Genes
404
85
70
158
2807
405
miBrain vs In Vivo
Monoculture vs In Vivo
miBrain vs Monoculture
J
Real-Time Monitoring Microglia-Vessel Dynamics
0 min
21 min
42 min
63 min
iMG-Surface / BMEC-Surface
K
Real-Time Monitoring Microglia-Neuron Dynamics
i
0 s
72 s
144 s
216 s
288 s
ii
iMG membrane / Tubulin

Fig. S19: Transcriptomic Analysis of miBrain-iMG. (**A**) Pre-labeled (FITC) iMG were isolated from miBrains using flow cytometry-based sorting of CD45-positive cells, (**B**) RNA-sequencing of iMG in miBrains and iMG monocultures: (**i**) genes per sample, (**ii**) total gene counts, (**iii**) percent of gene counts in the top 100 genes, and (**iv**) percent of gene counts in the top 500 genes, (**C**) gene expression of *P2RY12* in iMG for monocultures compared to miBrain, and (**D**) validation of P2RY12 differences in iMG between monoculture and miBrain via FACS analysis at the protein level, (**E**) top DEGs between iMG isolated from miBrains versus monocultures sorted by mean expression in miBrain, plotting TMM-normalized and scaled expression (FDR < 0.001), (**F**) volcano plot displaying these top DEGs between monocultured and miBrain iMG (left: upregulated in monocultured iMG, right: upregulated in miBrain-iMG), (**G**) correlation of miBrain- and monoculture-iMG to human *in vivo* prefrontal cortex microglia identified via snRNAseq of non-AD decedent tissue referencing the whole genome *(47)* (statistical significance determined via t test), and (**H**) correlation of miBrain- and monoculture-iMG to various microglial states for homeostatic and AD-pathological processes determined via snRNAseq across brain regions *(48)* (**i**) homeostatic and neuronal surveillance, (**ii**) inflammatory **I**, ribosome biogenesis, lipid processing, (**iii**) phagocytic, stress signature, glycolytic, (**iv**) inflammatory **II**, antiviral, and cycling states (statistical significance determined via t test), (**I**) Venn diagrams displaying DEGs in microglia between miBrain versus human *in vivo* (green), monoculture versus human *in vivo* (blue), and miBrain versus monoculture (red) for (i) downregulated and (ii) upregulated DEGs, (**J**) 3D, real-time monitoring of (top) iMG dynamics with vasculature in miBrain over 63 minutes (red: mCherry-BMECs-Surface, green: membrane pre-labeled iMG-Surface; scale bar, 10 μm) and (bottom) at higher magnification (scale bar, 5 μm), and (**K**) real-time monitoring of iMG dynamics with neurons in miBrains over 5 minute recordings of (i) example 1 and (ii) example 2 (cyan: tubulin, green: GFP-membrane-labeled iMG; scale bar, 30 μm).

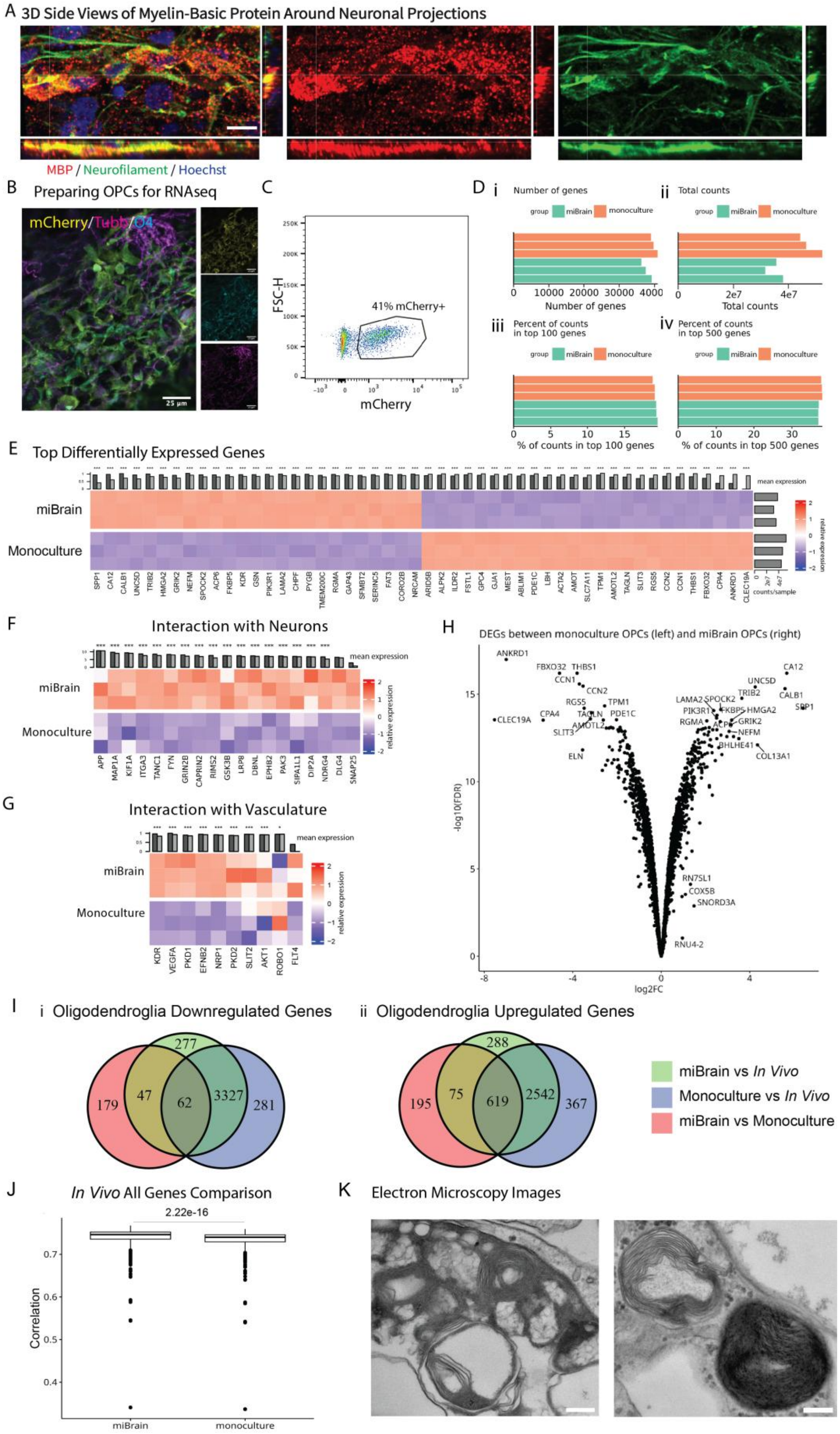
A 3D Side Views of Myelin-Basic Protein Around Neuronal Projections
MBP / Neurofilament / Hoechst
B Preparing OPCs for RNAseq
mCherry/Tubb/O4
25 μm
C
FSC-H
41% mCherry+
mCherry
D i Number of genes
ii Total counts
group miBrain monoculture
Number of genes
Total counts
iii Percent of counts in top 100 genes
iv Percent of counts in top 500 genes
% of counts in top 100 genes
% of counts in top 500 genes
E Top Differentially Expressed Genes
mean expression
miBrain
Monoculture
relative expression
counts/sample
SPP1 CA12 CALB1 UNC5D TRIB2 HMGA2 GRIK2 NEFM SPOCK2 ACP6 FKBP5 KDR GSN PIK3R1 LAMA2 CHPF PYGB TMEM200C RGMA GAP43 SFMBT2 SERINC5 FAT3 CORO2B NRCAM ARID5B ALPK2 ILDR2 FSTL1 GPC4 GJA1 MEST ABLIM1 PDE1C LBH ACTA2 AMOT SLC7A11 TPM1 AMOTL2 TAGLN SLIT3 RGS5 CCN2 CCN1 THBS1 FBXO32 CPA4 ANKRD1 CLEC19A
F Interaction with Neurons
mean expression
miBrain
Monoculture
relative expression
APP MAP1A KIF1A ITGA3 TANC1 FYN GRIN2B CAPRIN2 RIMS2 GSK3B LRP8 DBNL EPHB2 PAK3 SIPA1L1 DIP2A NDRG4 DLG4 SNAP25
G Interaction with Vasculature
mean expression
miBrain
Monoculture
relative expression
KDR VEGFA PKD1 EFNB2 NRP1 PKD2 SLIT2 AKT1 ROBO1 FLT4
H DEGs between monoculture OPCs (left) and miBrain OPCs (right)
-log10(FDR)
log2FC
ANKRD1 FBXO32 THBS1 CCN1 CCN2 TPM1 RGS5 TAGLN PDE1C CLEC19A CPA4 SLIT3 AMOTL2 ELN
CA12 UNC5D CALB1 SPP1 TRIB2 LAMA2 SPOCK2 PIK3R1 FKBP5 HMGA2 RGMA ACP6 GRIK2 NEFM BHLHE41 COL13A1
RN7SL1 COX5B SNORD3A RNU4-2
I i Oligodendroglia Downregulated Genes
ii Oligodendroglia Upregulated Genes
277 179 47 62 3327 281
288 195 75 619 2542 367
miBrain vs In Vivo
Monoculture vs In Vivo
miBrain vs Monoculture
J In Vivo All Genes Comparison
2.22e-16
Correlation
miBrain
monoculture
K Electron Microscopy Images

Fig. S20: Transcriptomic Analysis of miBrain-Oligodendroglia. (**A**) Side views of MBP-positive regions around neuronal projections (red: MBP, green: neurofilament, blue: Hoechst; scale bar, 10 μm), (**B**) mCherry-oligodendroglia incorporated into miBrains enabling mCherry-based isolation of oligodendroglia via flow cytometry (yellow: mCherry, magenta: TUBB, cyan: O4; scale bars, 25 μm), (**C**) gating of mCherry-positive cells in flow cytometry (**D**) RNA-sequencing of oligodendroglia in miBrains and oligodendroglia monocultures: (**i**) genes per sample, (**ii**) total gene counts, (**iii**) percent of gene counts in the top 100 genes, and (**iv**) percent of gene counts in the top 500 genes, (**E**) top DEGs between oligodendroglia isolated from miBrains versus monocultures sorted by mean expression in miBrain, plotting TMM-normalized and scaled expression (FDR < 0.001), (**F**) expression of key *in vivo*-like genes in RNAseq of oligodendroglia isolated from miBrain compared to monocultured oligodendroglia displayed as TMM-normalized and scaled expression for interactions with neurons and (**G**) interactions with vasculature, (**H**) volcano plot displaying these top DEGs between monocultured and miBrain oligodendroglia (left: upregulated in monocultured oligodendroglia, right: upregulated in miBrain-oligodendroglia), (**I**) Venn diagrams displaying DEGs in oligodendroglia between miBrain versus human *in vivo* (green), monoculture versus human *in vivo* (blue), and miBrain versus monoculture (red) for (i) downregulated and (ii) upregulated DEGs, (**J**) correlation of miBrain- and monoculture-oligodendroglia to human *in vivo* prefrontal cortex OPCs identified via snRNAseq of non-AD decedent tissue referencing the whole genome *(47)* (statistical significance determined via t test), and (**K**) electron microscopy images of miBrain (scale bar, 200 nm).

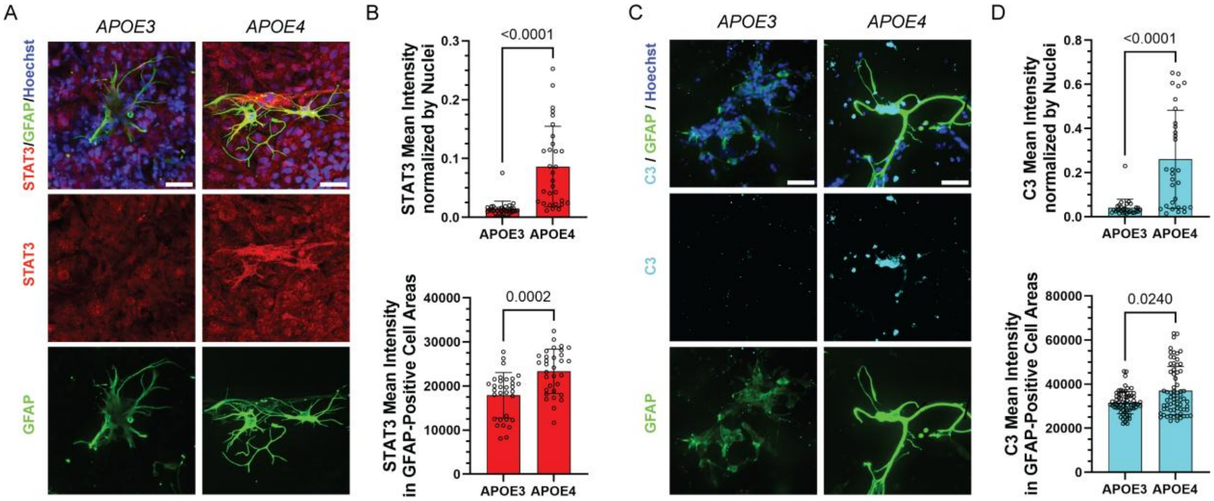

Fig. S21: Astrocyte-Associated Markers in *APOE3* and *APOE4* miBrains. (**A**) STAT3 immunoreactivity in *APOE3* and *APOE4* miBrains (red: STAT3, green: GFAP, blue: Hoechst; scale bars, 50 μm), (**B**) quantification for (*top*) STAT3 mean intensity normalized by nuclear area (n = 84 fields of view, Mann-Whitney t-test statistical test) and (*bottom*) STAT3 mean intensity in GFAP-positive regions (n = 32 fields of view, Mann-Whitney t-test statistical test), (**C**) C3 immunoreactivity in *APOE3* and *APOE4* miBrains (cyan: C3, green: GFAP, blue: Hoechst; scale bars, 50 μm), and (**D**) quantification for (*top*) C3 mean intensity normalized by nuclear area (n = 30 fields of view, Mann-Whitney t-test statistical test) and (*bottom*) C3 mean intensity in GFAP-positive regions (n = 68 fields of view, Mann-Whitney t-test statistical test).

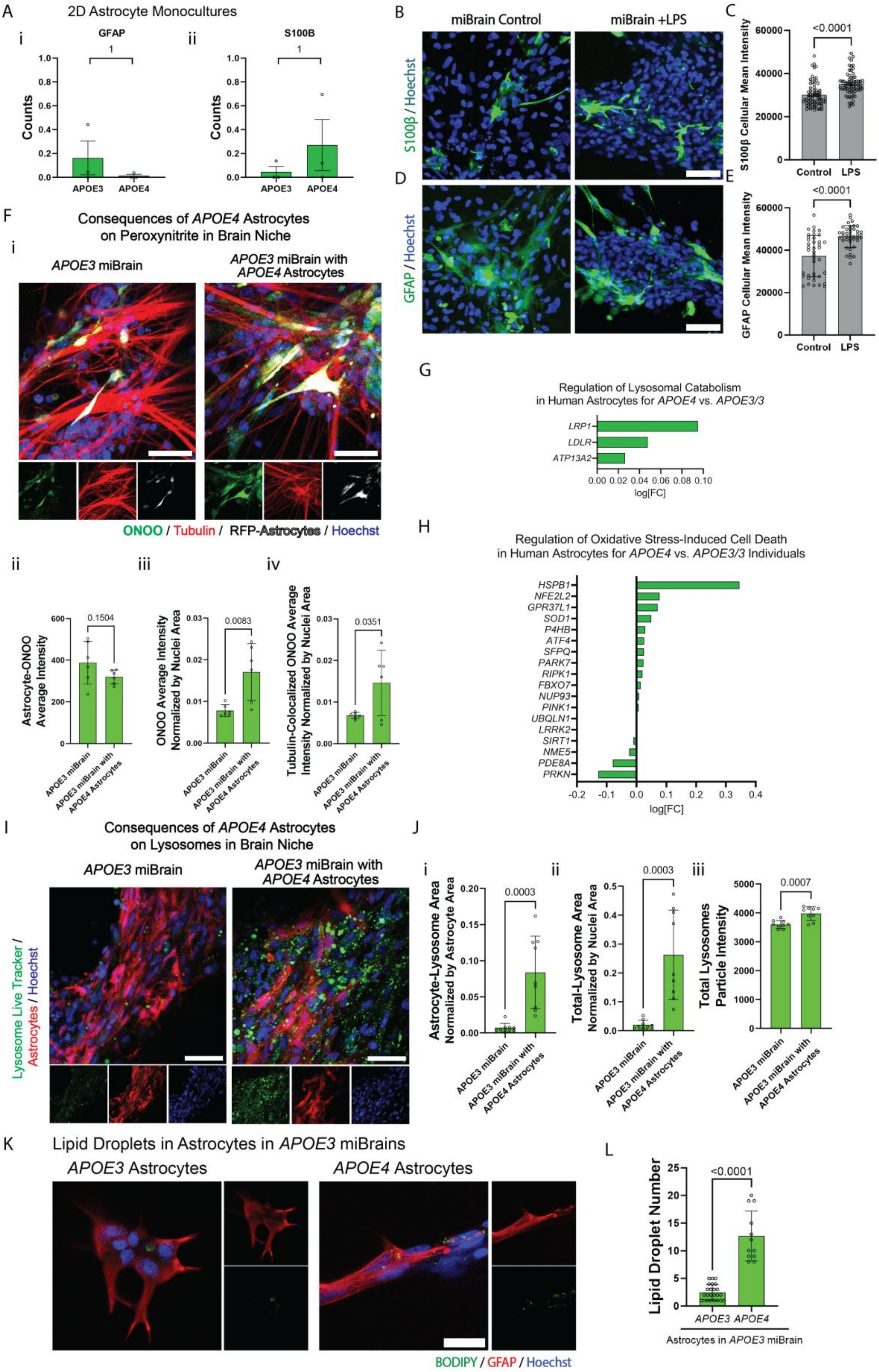
A
2D Astrocyte Monocultures
i
GFAP
1
Counts
APOE3 APOE4
ii
S100B
1
Counts
APOE3 APOE4
B
miBrain Control
miBrain +LPS
S100β / Hoechst
C
<0.0001
S100β Cellular Mean Intensity
Control LPS
D
GFAP / Hoechst
E
<0.0001
GFAP Cellular Mean Intensity
Control LPS
F
Consequences of APOE4 Astrocytes on Peroxynitrite in Brain Niche
i
APOE3 miBrain
APOE3 miBrain with APOE4 Astrocytes
ONOO / Tubulin / RFP-Astrocytes / Hoechst
ii
0.1504
Astrocyte-ONOO Average Intensity
iii
0.0083
ONOO Average Intensity Normalized by Nuclei Area
iv
0.0351
Tubulin-Colocalized ONOO Average Intensity Normalized by Nuclei Area
APOE3 miBrain
APOE3 miBrain with APOE4 Astrocytes
G
Regulation of Lysosomal Catabolism in Human Astrocytes for APOE4 vs. APOE3/3
LRP1
LDLR
ATP13A2
log[FC]
H
Regulation of Oxidative Stress-Induced Cell Death in Human Astrocytes for APOE4 vs. APOE3/3 Individuals
HSPB1
NFE2L2
GPR37L1
SOD1
P4HB
ATF4
SFPQ
PARK7
RIPK1
FBXO7
NUP93
PINK1
UBQLN1
LRRK2
SIRT1
NME5
PDE8A
PRKN
log[FC]
I
Consequences of APOE4 Astrocytes on Lysosomes in Brain Niche
APOE3 miBrain
APOE3 miBrain with APOE4 Astrocytes
Lysosome Live Tracker / Astrocytes / Hoechst
J
i
0.0003
Astrocyte-Lysosome Area Normalized by Astrocyte Area
ii
0.0003
Total-Lysosome Area Normalized by Nuclei Area
iii
0.0007
Total Lysosomes Particle Intensity
APOE3 miBrain
APOE3 miBrain with APOE4 Astrocytes
K
Lipid Droplets in Astrocytes in APOE3 miBrains
APOE3 Astrocytes
APOE4 Astrocytes
BODIPY / GFAP / Hoechst
L
<0.0001
Lipid Droplet Number
APOE3 APOE4
Astrocytes in APOE3 miBrain

Fig. S22: *APOE4* Astrocyte Dysregulation in miBrain. (**A**) Gene expression counts from 2D astrocyte monocultures from previously published RNA-seq dataset [13] for (i) *GFAP* and (ii) S100β, (**B**) S100b immunoreactivity in miBrains treated with 100 µg/mL LPS for 48 hours compared to untreated miBrains (green: S100b, blue: Hoechst; scale bar, 50 µm), (**C**) quantification of cellular mean intensity of S100b-positive cells in untreated and 100 µg/mL LPS treatment for 48 hours (n = 70 cells/ group; reported as mean and S.E.M., statistical significance via t test), (**D**) GFAP immunoreactivity in miBrains treated with LPS compared to untreated miBrains (green: GFAP, blue: Hoechst; scale bar, 50 µm), (E) quantification of cellular mean intensity of GFAP-positive cells in untreated and LPS-treated conditions (n = 40 cells/ group; reported as mean and standard deviation, statistical significance via t test), (**F**) peroxynitrite (ONOO) in miBrains with (**i**) *APOE3* (left) versus *APOE4* (right) astrocytes incorporated into otherwise-*APOE3* miBrains (green: ONOO, red: live tubulin label, gray: RFP-astrocytes, blue: Hoechst; scale bars, 50 µm), and (**ii-iv**) quantification of peroxynitrite in terms of (**ii**) mean intensity of peroxynitrite in astrocyte-colocalized regions, (**iii**) overall mean intensity of peroxynitrite normalized by nuclei area, and (**iv**) mean intensity of peroxynitrite in tubulin-colocalized region normalized by nuclei area (data from n = 3 replicates and images from n = 9 max projections from confocal z-stacks; statistical analysis via t test; experiment was conducted in 3 independent trials), (**G,H**) DEGs in human astrocytes from snRNAseq dataset [14] in *APOE3/4* and *APOE4/4* versus *APOE3/3* individuals (positive log[FC] is upregulated in *APOE4*) for pathway genes in (**G**) regulation of lysosomal protein catabolic process (GO:1905165) and (**H**) regulation (GO:1903201), positive regulation (GO:1903209), and negative regulation (GO:1903202) of oxidative stress-induced cell death, (**I**) lysosomes in miBrains with *APOE3* (left) versus *APOE4* (right) astrocytes incorporated into otherwise-*APOE3* miBrains (green: lysosome live tracker, red: RFP-astrocytes, blue: Hoechst; scale bars, 50 µm), and (**J**) quantification of lysosomes in terms of (**i**) area of lysosomes co-localized with astrocytes normalized by astrocyte area, (**ii**) total segmented lysosome area normalized by nuclei area, and (**iii**) intensity of segmented lysosome "particles" (data from n = 3 replicates and images from n = 9 fields of view; statistical analysis via t test; experiment was conducted in 3 independent trials), (**K**) lipid droplets in *APOE3* versus *APOE4* astrocytes in otherwise *APOE3* miBrains (green: BODIPY, red: GFAP, blue: Hoechst; scale bar, 30 µm), and (**L**) quantification of lipid droplet number for *APOE3* versus *APOE4* astrocytes in *APOE3* miBrains (n = 3 miBrains/ group; results shown on a per-cell basis; statistical significance via t test).

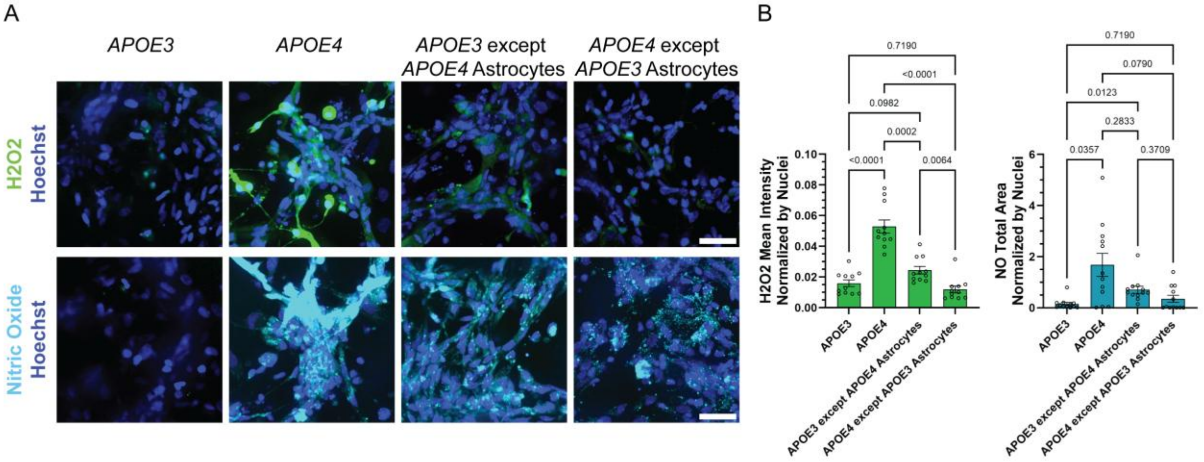

Fig. S23. Reactive Species Across miBrains with *APOE3* or *APOE4* Astrocytes. (**A**) (*Top*) Hydrogen peroxide and (*bottom*) nitric oxide in all-*APOE3* miBrain*,* all-*APOE4* miBrains*, APOE3* miBrains with *APOE4* astrocytes, and *APOE4* miBrains with *APOE3* astrocytes (green: hydrogen peroxide, cyan: nitric oxide, blue: Hoechst; scale bars, 50 µm) and (**B**) quantification for (*left*) hydrogen peroxide mean intensity normalized by nuclei (n = 11 fields of view) and (*right*) nitric oxide total positive area normalized by nuclei (n = 12 fields of view) (data presented as mean and S.E.M., Brown-Forsythe and Welch ANOVA statistical test).

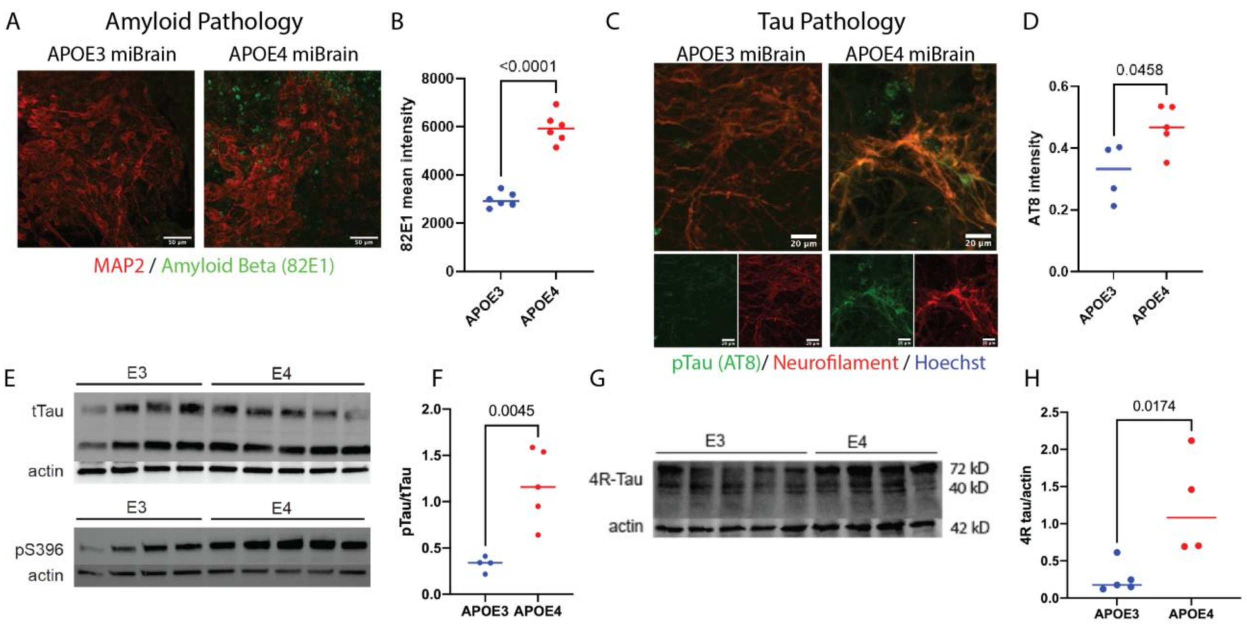

Fig. S24: Endogenous amyloid and tau pathologies in *APOE4* miBrain. (**A**) Amyloid aggregate accumulation in *APOE4* (right) compared to *APOE3* (left) miBrains at week 14 (green: amyloid 82E1, red: MAP2; scale bars, 50 µm), (**B**) quantification of mean intensity of 82E1-positive aggregates (n = 6; student's unpaired t test, p < 0.0001), (**C**) immunoreactivity to hyperphosphorylated tau in neurons in *APOE3* (left) versus *APOE4* miBrains (right) at 14 week (green: AT8, red: neurofilament, blue: Hoechst; scale bars, 50 µm), (**D**) quantification of hyperphosphorylated tau as intensity of AT8 signal co-localized with neurofilament-positive cells (n = 4-5; student's t test, p = 0.0458), (**E**) western blot analysis for total tau (tTau) and pS396 for *APOE3* and *APOE4* miBrains, using actin as a housekeeping probe for normalization, (**F**) quantification of pS396 between *APOE3* and *APOE4* miBrains, plotted as pTau normalized by tTau (n = 4-5; student's t test, p = 0.0458), (**G**) western blot analysis for 4R-Tau for *APOE3* and *APOE4* miBrains, using actin as a housekeeping probe for normalization, and (**H**) quantification of 4R-Tau bands (n = 4-5; student's t test, p = 0.0174).

Movie S1.

Visualization of neurovascular units and microglial integration throughout the miBrain with 3D reconstructions (cyan: neurons via tubulin label, red: BMECs, green: iMG via membrane label).

Movie S2.

Visualization of astrocytes integrated with neurons and with the blood-brain barrier throughout the miBrain with 3D reconstructions (green: mCherry-astrocytes, cyan: neurons via tubulin label, blue: Hoechst, red: ZO1-BMECs,).

Movie S3.

Visualization of oligodendroglia integrated with neurons and Fluoromyelin throughout the miBrain neurovascular units and microglial integration with 3D reconstructions (green: mCherry-oligodendroglia, red: FluoroMyelin, cyan: neurons via tubulin label).

SI References

[1] Y. T. Lin *et al.*, 'APOE4 Causes Widespread Molecular and Cellular Alterations Associated with Alzheimer's Disease Phenotypes in Human iPSC-Derived Brain Cell Types', *Neuron*, vol. 98, no. 6, pp. 1141–1154.e7, 2018, doi: 10.1016/j.neuron.2018.05.008.

[2] J. W. Blanchard *et al.*, 'Reconstruction of the human blood–brain barrier in vitro reveals a pathogenic mechanism of APOE4 in pericytes', *Nat Med*, vol. 26, no. 6, pp. 952–963, 2020, doi: 10.1038/s41591-020-0886-4.

[3] C. Patsch *et al.*, 'Generation of vascular endothelial and smooth muscle cells from human pluripotent stem cells', *Nat Cell Biol*, vol. 17, no. 8, pp. 994–1006, 2015, doi: 10.1038/ncb3205.

[4] H. Nishihara, B. D. Gastfriend, P. Kasap, S. P. Palecek, E. V. Shusta, and B. Engelhardt, 'Differentiation of human pluripotent stem cells to brain microvascular endothelial cell-like cells suitable to study immune cell interactions', *STAR Protoc*, vol. 2, no. 2, p. 100563, Jun. 2021, doi: 10.1016/j.xpro.2021.100563.

[5] J. TCW *et al.*, 'An Efficient Platform for Astrocyte Differentiation from Human Induced Pluripotent Stem Cells', *Stem Cell Reports*, vol. 9, no. 2, pp. 600–614, 2017, doi: 10.1016/j.stemcr.2017.06.018.

[6] P. Douvaras *et al.*, 'Efficient generation of myelinating oligodendrocytes from primary progressive multiple scelrosis patients by induced pluripotent stem cells', *Stem Cell Reports*, vol. 3, pp. 250–259, 2014, doi: http://dx.doi.org/10.1016/j.stemcr.2014.06.012.

[7] E. M. Abud *et al.*, 'iPSC-Derived Human Microglia-like Cells to Study Neurological Diseases', *Neuron*, vol. 94, no. 2, pp. 278-293.e9, 2017, doi: 10.1016/j.neuron.2017.03.042.

[8] Y. Zhang *et al.*, 'Rapid single-step induction of functional neurons from human pluripotent stem cells', *Neuron*, vol. 78, no. 5, pp. 785–798, 2013, doi: 10.1016/j.neuron.2013.05.029.

[9] Y. Yu and Y. Chau, 'One-Step "Click" Method for Generating Vinyl Sulfone Groups on Hydroxyl-Containing Water-Soluble Polymers', *Biomacromolecules*, vol. 13, no. 3, pp. 937–942, Mar. 2012, doi: 10.1021/bm2014476.

[10] H. Mathys *et al.*, 'Single-cell atlas reveals correlates of high cognitive function, dementia, and resilience to Alzheimer's disease pathology', *Cell*, vol. 186, no. 20, pp. 4365-4385.e27, Sep. 2023, doi: 10.1016/j.cell.2023.08.039.

[11] N. Sun *et al.*, 'Human microglial state dynamics in Alzheimer's disease progression', *Cell*, vol. 186, no. 20, pp. 4386-4403.e29, Sep. 2023, doi: 10.1016/j.cell.2023.08.037.

[12] D. Das *et al.*, 'Insights for disease modeling from single-cell transcriptomics of iPSC-derived Ngn2-induced neurons and astrocytes across differentiation time and co-culture', *BMC Biol*, vol. 22, no. 1, pp. 1–19, Dec. 2024, doi: 10.1186/S12915-024-01867-4/FIGURES/4.

[13] Y. T. Lin *et al.*, 'APOE4 Causes Widespread Molecular and Cellular Alterations Associated with Alzheimer's Disease Phenotypes in Human iPSC-Derived Brain Cell Types', *Neuron*, vol. 98, no. 6, pp. 1141-1154.e7, 2018, doi: 10.1016/j.neuron.2018.05.008.

[14] J. W. Blanchard *et al.*, 'APOE4 impairs myelination via cholesterol dysregulation in oligodendrocytes', *Nature*, vol. 611, no. 7937, pp. 769–779, Nov. 2022, doi: 10.1038/s41586-022-05439-w.
